# Metabolic Reprogramming in Bladder Cancer: Molecular Drivers, Immune Regulation and Therapeutic Opportunities

**DOI:** 10.3390/cancers18193202

**Published:** 2026-10-03

**Authors:** Shaolin Li, Minghui Li, Yinglang Zhang, Jie Pu, Jiaao Sun, Chunsheng Li, Changgang Guo

**Affiliations:** 1 Department of Urology, Affiliated Hospital of Chifeng University, Chifeng 024005, China; lishaolin2727@163.com (S.L.); lomo_hui@163.com (M.L.); zyl369337@sina.com (Y.Z.); 2 General Office, Affiliated Hospital of Chifeng University, Chifeng 024005, China; 15502813821@163.com; 3 Department of Urology, The First Affiliated Hospital of Dalian Medical University, Dalian 116011, China; sja466645259@163.com

**Keywords:** bladder cancer, metabolic reprogramming, pathogenesis, chemotherapy, immunotherapy, biomarker

## Abstract

Metabolic reprogramming is increasingly recognised as a central determinant of bladder cancer heterogeneity, immune regulation and treatment response. This review integrates genetic, epigenetic, microenvironmental and therapeutic evidence to explain how bladder cancer redistributes carbon and nitrogen across glycolysis, lipid synthesis and oxidation, amino-acid metabolism and mitochondrial respiration. Alterations in *FGFR3*, *PIK3CA*, *MYC*, *TP53*/*RB1* and *KDM6A* establish distinct, context-dependent metabolic dependencies, while hypoxia, extracellular-matrix mechanics and treatment selection reinforce metabolic plasticity. These programmes influence not only energy production and biosynthesis but also ferroptosis, genome stability and chromatin or RNA regulation. Lactate, adenosine, kynurenine and asparagine further connect tumour metabolism to regulatory T cells, myeloid cells, innate immune sensing and CD8-positive T cell function, thereby shaping resistance to chemotherapy and immune-checkpoint blockade. Therapeutic opportunities include targeting glycolysis, fatty-acid and cholesterol metabolism, glutamine and proline metabolism, arginine auxotrophy and metabolite-mediated immune suppression. However, pathway redundancy, cellular heterogeneity and shared metabolic requirements between tumour and immune cells constrain unselected treatment. Urine, tissue, blood and imaging metabolomics offer complementary routes to diagnosis, prognostication and real-time response monitoring, but require harmonised sampling, functional flux measurements and prospective validation. A clinically useful strategy will therefore depend on defining metabolic vulnerabilities by genotype, cell type, spatial context and treatment stage, and on pairing pharmacodynamic biomarkers with rational combinations and local delivery where appropriate.

## Simple Summary

Bladder cancer remains difficult to treat because tumours often stop responding to therapy. This review explains that cancer cells change the way they use nutrients—such as sugars, fats and proteins—to fuel their growth, resist treatment and evade the immune system. The authors bring together recent evidence to show how genetic changes, the tumour environment and treatment itself drive these metabolic shifts. Understanding these processes may help doctors identify which patients are most likely to benefit from specific therapies and design new treatment strategies that target the cancer’s altered metabolism. By linking metabolism to treatment response, this work aims to guide more precise, personalised approaches for managing bladder cancer.

## 1. Introduction

Bladder cancer (BC) accounts for a substantial and growing proportion of the global cancer burden. In this review, BC refers primarily to urothelial carcinoma of the urinary bladder. Evidence involving variant urothelial histologies or non-urothelial models is identified explicitly where relevant, whereas primary non-urothelial bladder malignancies are not systematically reviewed. The latest estimates from the Global Cancer Observatory (GLOBOCAN) indicate that approximately 635,000 new cases and 228,000 deaths occurred worldwide in 2024, with men accounting for the large majority [1]. Clinically, BC comprises two closely related disease states with markedly different biological behaviour and therapeutic priorities. Non-muscle-invasive bladder cancer (NMIBC) is generally controllable but has a high risk of recurrence, necessitating repeated cystoscopy, transurethral resection and risk-adapted intravesical therapy [2]. Muscle-invasive bladder cancer (MIBC) is less readily detected and carries substantially greater risks of dissemination and death [3]. Beyond surgery, avelumab maintenance after disease control with platinum-based chemotherapy prolongs overall survival [4], while enfortumab vedotin plus pembrolizumab has outperformed platinum-based chemotherapy in previously untreated patients [5]. Nevertheless, these therapeutic advances have not resolved the central problem of recurrence. In the SWOG-8710 randomised trial, neoadjuvant methotrexate, vinblastine, doxorubicin and cisplatin followed by radical cystectomy produced a pathological complete response (pT0) in 38% of patients, compared with 15% after cystectomy alone [3]. Resistance and relapse are frequent, subsequent progression is common, durable benefit is confined to a subset of patients, and clinically actionable biomarkers of adaptive response remain scarce [3,4,5]. Unlike resistance that depends on the selection and expansion of genetically altered clones, metabolic adaptation can arise through reversible changes in enzyme activity, substrate use and mitochondrial function, allowing a resistant phenotype to emerge within hours of treatment exposure [6].

The aetiological relationship between the bladder and metabolism is more direct than that of many other solid organs. Tobacco smoke, occupational aromatic amines, arsenic and other environmental carcinogens enter the circulation and, after enzymatic activation or detoxification, may become concentrated in urine, repeatedly exposing the urothelium to pathogenic compounds and their reactive metabolites [7]. This distinctive anatomy and physiology make bladder carcinogenesis an evolving process that links environmental exposure, biotransformation and tissue response. Genomic studies provide a molecular explanation for this disease context. *FGFR3* alterations and luminal differentiation programmes are enriched in papillary and luminal tumours, whereas *TP53*/*RB1* inactivation, basal or squamous differentiation, stromal remodelling and inflammatory programmes are common in aggressive MIBC. Recurrent alterations in chromatin regulators, phosphoinositide 3-kinase (PI3K) signalling, cell-cycle control and lineage transcription factors further diversify the routes by which urothelial cells acquire malignant fitness [8,9,10]. Metabolism is not merely a prerequisite for tumour-cell proliferation but an active determinant of transformation and progression. Metabolically reprogrammed tumour cells redistribute glucose, amino acids, fatty acids and mitochondrial substrates to meet biosynthetic and redox demands; metabolites, in turn, reshape tumour behaviour by regulating chromatin, RNA modification, protein stability, cell death and immune-cell function [11]. In BC, chronic urinary exposure, repeated invasive treatment and a spatially heterogeneous microenvironment impose persistent metabolic stress. This stress may foster metabolic plasticity, allowing tumour cells to switch among energy sources and adaptive programmes as nutrient availability, oxygenation and treatment pressure change. Defining the drivers and downstream consequences of this reprogramming may therefore clarify biological heterogeneity and provide a rationale for overcoming resistance and developing precision interventions.

This review integrates recent advances in BC metabolism into a framework linking genetic alterations, cell states, metabolic networks and treatment response. We first examine how driver genes, canonical signalling pathways, epigenetic regulation and the mechanical microenvironment shape metabolic phenotypes across molecular subtypes. We then consider the reconfiguration of glucose, lipid, amino-acid and mitochondrial metabolism, together with metabolite-mediated immune regulation. Next, we summarise the metabolic adaptations selected by chemotherapy, targeted therapy and immunotherapy and assess strategies directed at key metabolic nodes and metabolic–immune interaction axes. Finally, we discuss the opportunities and limitations of tissue-, blood-, urine- and imaging-based biomarkers and integrated multi-omics approaches. This synthesis emphasises that metabolic reprogramming in BC is not the isolated activation of a single pathway, but a dynamic network jointly determined by genetic background, cell lineage, microenvironmental pressure and therapeutic selection. A deeper understanding of this network may enable more precise patient stratification and metabolically guided treatment.

This narrative review was informed by searches of PubMed/MEDLINE, Web of Science Core Collection and Scopus from database inception to 31 August 2026. Search terms combined “bladder cancer” or “urothelial carcinoma” with terms related to metabolic reprogramming, glycolysis, lipid metabolism, amino-acid metabolism, mitochondrial metabolism, metabolomics, treatment resistance, immunotherapy and biomarkers. Peer-reviewed original studies, clinical trials and relevant reviews were considered, with priority given to recent BC-specific evidence. Studies in other tumour types were included only when they provided mechanistic or clinical context not available in BC. Duplicate publications, reports without direct relevance to cancer metabolism and studies lacking sufficient methodological or outcome information were excluded. Reference lists of eligible articles were screened to identify additional relevant studies. Evidence was classified according to its source, including BC cell lines, organoids, animal models, patient samples, clinical studies and computational analyses. Throughout this review, studies performed in non-urothelial tumour types are explicitly identified as cross-cancer evidence and are used only to provide biological rationale; they should not be interpreted as direct evidence of efficacy in bladder cancer.

## 2. Molecular Basis of Metabolic Reprogramming in Bladder Cancer

### 2.1. Genetically Driven Metabolic Dependencies

#### 2.1.1. FGFR3 and Lipid Metabolism

Fibroblast growth factor receptor 3 (FGFR3) is a cell-surface receptor tyrosine kinase that activates downstream pathways, including mitogen-activated protein kinase (MAPK) and PI3K/protein kinase B (AKT), after ligand binding. *FGFR3* mutations are markedly enriched in low-grade papillary NMIBC and luminal-papillary MIBC [8,9,10]. Proteogenomic studies further show that *FGFR3* alterations define tumours spanning NMIBC and MIBC, but these tumours differ in apoptotic sensitivity, membrane-lipid homeostasis and cell state [12,13,14]. *FGFR3* genotype is therefore a starting point, rather than a sufficient criterion, for metabolic stratification. The strongest mechanistic evidence supports an FGFR3-PI3K-mechanistic target of rapamycin complex 1 (mTORC1)-sterol regulatory element-binding protein 1 (SREBP1)-stearoyl-CoA desaturase 1 (SCD1) axis. Activated FGFR3 promotes SREBP1 maturation and nuclear translocation through PI3K-mTORC1, thereby inducing *SCD1* and other lipogenic genes. SCD1 converts saturated fatty acyl-CoA species into monounsaturated fatty acids, maintaining membrane fluidity and alleviating saturated-fatty-acid-induced endoplasmic reticulum (ER) stress. SCD1 inhibition induces cell-cycle arrest and apoptosis and suppresses the growth of FGFR3-positive xenografts [15]. In gemcitabine-resistant models, FGFR inhibition also reduces malonyl-CoA and fatty acid synthase (FASN)-associated lipogenesis and synergises with phosphoglycerate dehydrogenase (PHGDH) inhibition [16]. These findings connect FGFR3-driven lipogenesis with cholesterol homeostasis, lysosomal recycling and treatment tolerance. However, evidence for effects of FGFR3 on glucose metabolism and mitochondrial carbon flux still derives largely from a small number of cell lines and resistance models [15,16,17]. Moreover, some *FGFR3*-mutant tumours are highly apoptosis-prone and do not share the same lipid dependency [12]. Metabolic therapies guided by *FGFR3* should therefore be combined with tumour lipid profiling and functional metabolic assessment.

#### 2.1.2. MYC-Driven Metabolic Reprogramming

*MYC* is a canonical oncogene encoding a multifunctional transcription factor. *MYC* amplification or overexpression occurs in several urothelial cancer contexts, and high MYC activity is generally associated with strong proliferative signalling [8,10,18]. Binding of the MYC–MAX complex to E-box elements simultaneously induces genes involved in glucose transport, glycolysis, lactate production, nucleotide synthesis, and glutamine uptake and catabolism. This programme diverts glucose into the pentose phosphate, serine/one-carbon and lipid-synthesis pathways, while glutamine replenishes the tricarboxylic acid (TCA) cycle and supports glutathione (GSH) and nicotinamide adenine dinucleotide phosphate (NADPH) homeostasis. In BC, MYC activity is also associated with hexokinase 2 (HK2) expression, hybrid energy metabolism and intratumour heterogeneity [13,19,20,21,22]. Because MYC itself is difficult to target directly, attention has shifted to mechanisms that maintain its stability. Ubiquitin-specific peptidase 43 (USP43) removes K48-linked ubiquitin chains from c-MYC and prevents F-box and WD repeat domain-containing 7 (FBXW7)-mediated proteasomal degradation. Stabilised c-MYC then binds the *USP43* promoter and promotes its transcription, establishing a positive feedback loop that increases glucose consumption [23], lactate production and metastasis through lactate dehydrogenase A (LDHA) upregulation [24]. Cell division cycle-associated protein 3 (CDCA3) recruits tripartite motif-containing 28 (TRIM28) to reduce MYC ubiquitination; MYC subsequently induces *CDCA3* and sustains glycolysis and tumour growth through enolase 1 (ENO1) [25]. Discs large-associated protein 5 (DLGAP5) likewise promotes glycolysis and gemcitabine resistance by stabilising MYC [19]. Under high-glucose conditions, HK2 can also enter the nucleus and form a transcriptional complex with MYC, further amplifying glycolysis and tumour stemness [20]. Thus, MYC dependency reflects not only increased transcriptional activity but also multilayered control through deubiquitination, protein degradation and subcellular localisation.

#### 2.1.3. KDM6A Loss Suppresses Glycolysis

Lysine demethylase 6A (KDM6A) is a frequently altered chromatin regulator in BC and a component of COMPASS/MLL3/4-associated complexes. In addition to catalysing H3K27me3 demethylation, KDM6A participates in enhancer occupancy and differentiation-related transcription; its binding landscape and interacting proteins vary with mutational status and bladder-cell context [8,10,26]. As an α-ketoglutarate (alpha-KG)-dependent dioxygenase, KDM6A is jointly constrained by oxygen availability, alpha-KG and competitive metabolites, allowing hypoxia or altered cofactor supply to transmit metabolic state to chromatin [27,28,29,30]. Recent CRISPR-engineered human and murine models revealed two mechanistically distinct consequences of *KDM6A* loss. At the tumour-cell-intrinsic level, *KDM6A* deficiency impairs double-strand-break and mismatch repair and increases extrachromosomal circular DNA carrying resistance loci, thereby promoting cisplatin resistance. The same models show reduced activity of glycolytic genes, including *HK2*, with lower glycolytic ATP production, extracellular acidification and lactate release [31].

At the cell-extrinsic level, reduced tumour-derived lactate decreases H3K9/H3K18 lactylation and *FOXP3* and *TGFB1* expression in Treg cells, while limiting the expansion of PD-1-high Treg cells. This remodelling of the immune microenvironment is associated with improved responses to anti-PD-1 therapy [31]. Thus, genomic instability and impaired DNA repair explain the chemotherapy phenotype, whereas reduced lactate-mediated immunosuppression explains the immunotherapy phenotype. These effects remain contingent on molecular subtype, sex-chromosome dosage, co-mutations and the redox environment [8,9,27,28,29,30,31,32]. A fixed metabolic or therapeutic phenotype cannot therefore be inferred from *KDM6A* status alone.

Taken together, *FGFR3*, *MYC* and *KDM6A* shape metabolic phenotypes through receptor signalling, transcriptional control and chromatin state, respectively. Although they alter carbon flux in distinct ways, each changes how tumour cells respond to nutrient limitation, oxidative stress and treatment pressure. This genetically determined metabolic plasticity constitutes a first layer of metabolic heterogeneity in BC.

### 2.2. Oncogenic Signalling Drives Metabolic Adaptation

Oncogenic signals in BC do not operate as parallel switches. They form an interconnected network through shared transcription factors, metabolic enzymes and substrate pools. Wnt–β-catenin signalling can expand glucose and glutamine utilisation through MYC; PI3K–AKT-mammalian target of rapamycin (mTOR) enhances glycolysis and lipogenesis through hypoxia-inducible factor 1 alpha (HIF-1α) and SREBP, respectively; and RAS-MAPK regulation of pyruvate kinase M2 (PKM2) conformation and nuclear function links glucose metabolism to angiogenesis. SRC, the mevalonate pathway and protein degradation systems that stabilise MYC may preserve part of the metabolic output when a dominant pathway is inhibited [14,17,19,21,33,34,35]. These intersections help explain why single-pathway inhibitors often fail to produce durable responses.

#### 2.2.1. Wnt–β-Catenin Signalling

Canonical Wnt signalling first alters beta-catenin stability and nuclear translocation. In the absence of Wnt ligands, a destruction complex comprising adenomatous polyposis coli (APC), axis inhibition protein (AXIN), casein kinase 1 (CK1) and glycogen synthase kinase 3 beta (GSK3beta) sequentially phosphorylates beta-catenin. Beta-transducin repeat-containing protein (beta-TrCP) recognises the phosphorylated degron and directs beta-catenin to the ubiquitin–proteasome pathway. When Wnt binds Frizzled and low-density lipoprotein receptor-related protein 5/6 (LRP5/6), phosphorylation of the LRP co-receptor recruits AXIN and inhibits the destruction complex, allowing beta-catenin to accumulate, enter the nucleus and cooperate with T-cell factor/lymphoid enhancer-binding factor (TCF/LEF) to activate targets such as *MYC* [36,37]. In BC, ligand-independent inputs can reinforce this process. The androgen receptor promotes beta-catenin activation and nuclear translocation, enhancing *MYC* transactivation by an androgen receptor-TCF complex [38], while the ER-stress transcription factor X-box-binding protein 1 (XBP1) increases beta-catenin expression to sustain stemness, invasion and chemoresistance [39]. *MYC* induction downstream of Wnt–β-catenin simultaneously increases glucose uptake, glycolysis, glutaminolysis and nucleotide synthesis, extending Wnt output from lineage maintenance to intermediary metabolism. The LRP8–Wnt–β-catenin–SCD1 axis provides more direct evidence for lipid regulation. LRP8 activates beta-catenin and induces *SCD1*, which desaturates saturated fatty acids to monounsaturated fatty acids. Incorporation of these products into membrane phospholipids dilutes readily oxidised polyunsaturated fatty acids and thereby reduces lipid peroxidation and ferroptosis. *LRP8* knockdown increases ferrous iron, malondialdehyde and lipid reactive oxygen species (ROS); Wnt or *SCD1* reconstitution reverses these phenotypes and restores xenograft growth [40]. The mechanical microenvironment regulates the same transcriptional node. Integrin tension generated by matrix stiffening enlarges nuclear pores and increases lamin A/C, facilitating beta-catenin entry and prolonging its nuclear residence, with consequent maintenance of stemness, invasion and cisplatin tolerance [41,42,43]. Although the mevalonate pathway and lipid uptake may also alter cytoskeletal organisation and signal transduction through Rho-integrin signalling or membrane composition [33,44,45], direct attribution of these effects to beta-catenin activity in BC remains insufficiently supported.

#### 2.2.2. USP43–c-MYC Signalling

USP43 provides a direct mechanism by which BC cells sustain glycolysis through c-MYC stabilisation [24]. Under normal conditions, FBXW7 recognises phosphorylated c-MYC and promotes K48-linked polyubiquitination, followed by proteasomal degradation. USP43 removes these chains, prolonging the c-MYC half-life. Accumulated c-MYC then binds the *USP43* promoter and stimulates its transcription, creating a self-reinforcing feedback loop [24]. This loop upregulates *LDHA*, diverts more pyruvate to lactate and maintains glycolytic continuity by regenerating nicotinamide adenine dinucleotide (NAD+). The resulting increase in lactate and proton export creates an acidic environment that favours invasion and immunosuppression. USP43 is not the only determinant of MYC stability. CDCA3 recruits TRIM28 to reduce MYC ubiquitination; stabilised MYC then induces *CDCA3* and increases glycolysis through ENO1 [25]. DLGAP5 likewise increases glucose consumption and lactate production by stabilising MYC and promotes gemcitabine resistance [19]. These parallel mechanisms indicate that USP43 inhibition is more likely to weaken one node in the MYC-stability network than to abolish MYC output.

#### 2.2.3. TP53–RB1 Signalling

Tumour protein p53 (*TP53*) and RB transcriptional corepressor 1 (*RB1*) constitute the most commonly co-inactivated tumour-suppressor pathways in MIBC, particularly in basal/squamous, neuroendocrine-like and highly proliferative tumours [8,9,10]. Wild-type p53 induces the synthesis of cytochrome c oxidase 2 (SCO2), supporting cytochrome c oxidase complex assembly and mitochondrial respiration. In parallel, the p53 target TP53-induced glycolysis and apoptosis regulator (TIGAR) lowers fructose-2,6-bisphosphate, reduces phosphofructokinase 1 (PFK1) activity and diverts part of the glucose-6-phosphate pool into the pentose phosphate pathway (PPP) to generate NADPH [46,47]. RB1 restrains the cell cycle and anabolism by binding E2F transcription factors; loss of *RB1* releases E2F, increasing DNA replication, nucleotide synthesis and the associated demand for energy and reducing equivalents. Co-inactivation of *TP53* and *RB1* removes both proliferative restraints and metabolic-stress checkpoints, but current evidence does not justify assigning every double-null tumour a uniformly glycolytic or oxidative phenotype. The E2F1-responsive long non-coding RNA (lncRNA) *SLC16A1-AS1* provides a testable mechanism of multi-substrate utilisation. It is both an E2F1 target and an E2F1-binding partner and upregulates *SLC16A1*/monocarboxylate transporter 1 (MCT1) and peroxisome proliferator-activated receptor alpha (PPARalpha)-related genes. *SLC16A1-AS1* knockdown simultaneously reduces glycolysis, oxidative phosphorylation (OXPHOS) and fatty-acid oxidation and suppresses tumour growth and invasion [48]. Aberrant p53 is also associated with an immunoregulatory macrophage population expressing *SPP1*, *MARCO*, *SLC2A1* and *INHBA*. These cells display a pronounced lipolytic programme and correlate with poor outcomes after platinum or PD-L1-directed therapy [49]. Thus, the metabolic consequences of *TP53*/*RB1* abnormalities arise from tumour-cell-intrinsic E2F programmes and may be further amplified by myeloid-cell states.

#### 2.2.4. PI3K–AKT–mTOR Signalling

The PI3K–AKT–mTOR pathway converts growth-factor, nutrient and energy signals into anabolic output. Persistent activation of receptor tyrosine kinases such as FGFR3 and ERBB, *PIK3CA* hotspot mutations, or phosphatase and tensin homologue (*PTEN*) loss can activate this pathway. Downstream AKT promotes glucose transport and glycolytic-enzyme activity, while mTORC1 induces glycolytic genes through HIF-1α and activates SREBP1/2 through ribosomal protein S6 kinase beta-1 (S6K1), thereby promoting fatty-acid and cholesterol synthesis [8,9,10,50]. The pathway therefore coordinates glucose uptake, the PPP and membrane-lipid production rather than controlling a single substrate. A *PIK3CA* E545K knock-in model directly linked signalling activity to altered carbon flux. Mutant PI3Kalpha upregulated glutaminase 1 (GLS1) and FASN, enabling GLS1 to convert glutamine into glutamate and alpha-KG. One fraction of alpha-KG entered the oxidative TCA cycle to support complex I and OXPHOS, whereas another underwent isocitrate dehydrogenase (IDH)-dependent reductive carboxylation to isocitrate and citrate, supplying acetyl-CoA for FASN. Isotope tracing confirmed transfer of glutamine carbon into citrate and fatty acids; inhibition of PI3Kalpha, GLS1 or complex I attenuated this phenotype and reduced orthotopic tumour growth [51]. FGFR3-positive and gemcitabine-resistant models likewise show that FGFR–PI3K–mTOR signalling links lipid synthesis and serine metabolism through SREBP1–SCD1, FASN and PHGDH [15,16]. When mTOR is inhibited, autophagy transiently replenishes amino-acid and lipid substrates. At the same time, membrane-lipid stress caused by reduced cholesterol synthesis increases vulnerability to lysosomal inhibition, explaining the synergy between mTOR inhibition and chloroquine in BC cells [17].

### 2.3. Bidirectional Regulation Between Metabolism and Epigenetics

Cellular metabolism continuously supplies substrates and cofactors for chromatin and RNA modification. Alpha-KG supports Jumonji C domain-containing (JmjC) demethylases and ten-eleven translocation (TET) dioxygenases; acetyl-CoA supports histone acetylation; S-adenosylmethionine (SAM) donates methyl groups; NAD+ regulates sirtuin activity; and lactate can drive lysine lactylation [27,29,30,52,53]. Metabolite availability consequently changes enzyme activity and chromatin accessibility, while chromatin and RNA modifications reciprocally control metabolic enzymes and transporters. The JMJD1A–HIF-1α axis provides a direct example in BC. Hypoxia activates HIF-1α and recruits JMJD1A to target promoters, where it reduces repressive histone methylation and increases the expression of glucose transporter 1 (*GLUT1*), *HK2*, phosphoglycerate kinase 1 (*PGK1*), phosphoglucomutase (PGM), *LDHA* and monocarboxylate transporter 4 (*MCT4*), sustaining glucose uptake, glycolysis and lactate export [54]. RNA 5-methylcytosine (m5C) modification stabilises metabolic-enzyme transcripts post-transcriptionally. HIF-1α induces Aly/REF export factor (ALYREF), which recognises m5C sites in *PKM2* messenger RNA (mRNA), increases transcript stability and thereby maintains *PKM2* expression and glycolysis [55]. Nucleolar protein 2 (NOP2) similarly catalyses the m5C modification of *SCD* mRNA; Y-box binding protein 1 (YBX1) recognises the mark and stabilises the transcript, increasing *SCD* and monounsaturated fatty acids, reducing the proportion of readily oxidised fatty acids in membrane phospholipids and ultimately limiting lipid peroxidation and ferroptosis [56]. Lactate accumulation also promotes histone lysine lactylation. In BC models, LDHA-driven lactate elevation parallels increased histone H3 lysine 18 lactylation (H3K18la); inhibiting glycolysis or silencing *LDHA* reduces this modification and suppresses tumour growth [52]. Cleavage under targets and tagmentation (CUT&Tag) combined with RNA sequencing (RNA-seq) showed H3K18la enrichment at *AHNAK2*, *PVR*, *SLC7A11* and *SREBF1* loci, linking lactate production to invasion, immune escape, cysteine uptake and lipid synthesis, respectively [57]. Hypoxia-induced pyrroline-5-carboxylate reductase 1 (PYCR1) further increases lactate and H3K18la, inducing *SLC6A14* transcription and glutamine uptake. The resulting PYCR1–lactate–H3K18la–SLC6A14 feedback loop promotes tumour growth and metastasis [58]. Metabolic–epigenetic coupling is therefore not a unidirectional supply of substrates but a set of feedback circuits that stabilise specific metabolic states.

### 2.4. Metabolic Heterogeneity Across Disease Stages and Molecular Subtypes

The metabolic distinction between NMIBC and MIBC is not a simple gradient of pathway intensity. Low-grade papillary NMIBC commonly harbours *FGFR3* alterations and can enhance fatty-acid desaturation through SREBP1-SCD1. MIBC more often combines *TP53*/*RB1* loss, hypoxia, stromal remodelling and treatment selection, allowing glycolysis, glutamine anaplerosis, OXPHOS and lipid metabolism to operate in parallel [8,9,15,16]. Cross-stage proteogenomic studies have identified molecular groups spanning NMIBC and MIBC and have shown that *FGFR3*-altered, papillary and carcinoma-in situ tumours differ in protein expression and apoptotic sensitivity [12,14]. Deep proteomic profiling further indicates that, relative to NMIBC, MIBC is enriched in extracellular-matrix and immune-related proteins and displays altered lipid metabolism, although both disease classes contain metabolic, stromal and immune subgroups [12,59]. Tissue metabolomics likewise reveals both differences and overlap in amino-acid, organic-acid and lipid profiles [60]. Thus, even within the same clinical stage, luminal, basal/squamous and stroma-rich tumours may differ in nutrient use, stromal composition and immune infiltration [9,12,13,14]. Computational classification based on glycolytic and cholesterol synthesis genes separates tumours into quiescent, glycolytic, cholesterogenic and mixed subtypes. The mixed subtype more often displays basal features, M0/M2 macrophage infiltration and poor prognosis [61]. Peroxisome proliferator-activated receptor gamma (PPARγ) links lineage, lipid metabolism and immune state. PPARγ–RXRα activation maintains luminal differentiation and lipid metabolism while suppressing NF-κB-related inflammation and chemokine expression, thereby reducing effector-cell infiltration and promoting an immune-excluded phenotype [62]. Clinical stage therefore cannot substitute for metabolic classification, and transcriptomic metabolic labels should be interpreted alongside protein, metabolite and spatial immune information.

### 2.5. Mechanical Microenvironment and Metabolic Regulation

**Figure 1:**
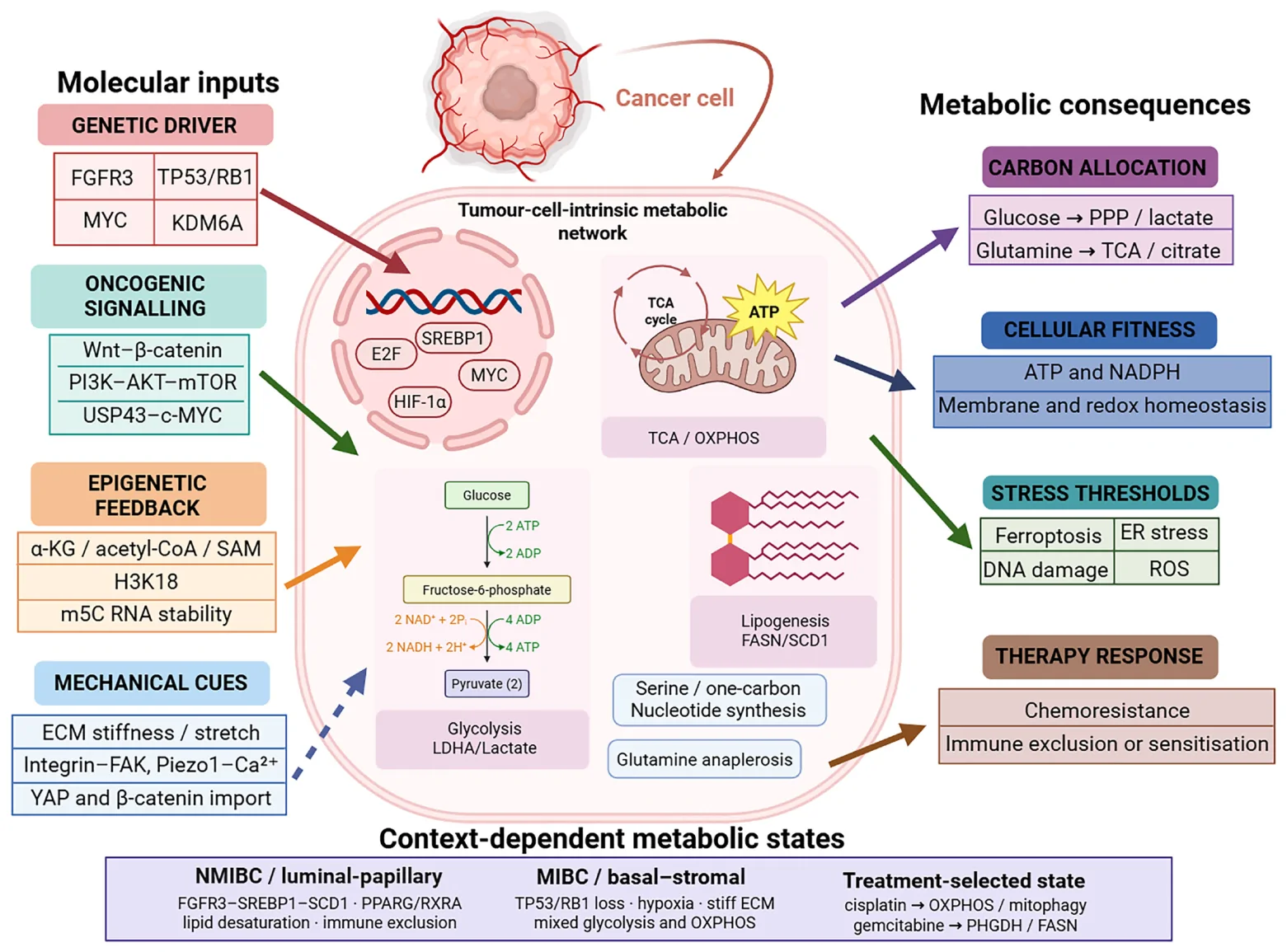
Molecular architecture of metabolic reprogramming in bladder cancer. Genetic drivers, oncogenic signalling, metabolite-sensitive epigenetic regulation and mechanical cues converge on transcriptional effectors that redistribute carbon use. FGFR3 and PI3K–AKT–mTOR signalling promote SREBP1-SCD1-dependent lipid desaturation, whereas Wnt–β-catenin and MYC expand glucose and glutamine utilisation. *TP53*/*RB1* loss releases E2F-associated anabolic programmes. *KDM6A* loss can simultaneously alter glycolysis, lactate availability, oxidative phosphorylation and genome stability, precluding a unidirectional interpretation of its therapeutic effects. A-ketoglutarate, acetyl-CoA, S-adenosylmethionine and lactate couple nutrient availability to chromatin and RNA regulation, which in turn controls metabolic enzymes and transporters. Extracellular-matrix stiffness and stretch signal through integrin-FAK, Piezo1-calcium, YAP and beta-catenin, although some mechanical-to-flux connections remain to be demonstrated directly in bladder cancer. These inputs alter glycolysis, serine and one-carbon metabolism, glutamine anaplerosis, the TCA cycle and oxidative phosphorylation, and lipogenesis, thereby changing ATP and NADPH supply, membrane and redox homeostasis, stress thresholds and treatment response. Solid arrows indicate mechanistic links supported in bladder cancer models. The single dashed arrow, connecting mechanical cues with central carbon-flux reprogramming, indicates that extracellular-matrix stiffness and stretch can activate YAP and beta-catenin signalling, whereas direct validation of their effects on net metabolic flux in bladder cancer remains incomplete. Disease context resolves the network into distinct metabolic states. *FGFR3*/*PPARG*-enriched non-muscle-invasive, luminal-papillary tumours favour SREBP1–SCD1-dependent lipid desaturation and immune exclusion. *TP53*/*RB1* loss, hypoxia and a stiff stroma jointly support a mixed glycolytic and oxidative state in muscle-invasive, basal-stromal tumours. Treatment selection can further favour oxidative phosphorylation and mitophagy after cisplatin exposure or PHGDH- and FASN-dependent biosynthesis after gemcitabine exposure.

Cyclical filling and emptying of the bladder continuously expose urothelial and tumour cells to stretch, fluid shear and changes in urinary osmolarity, while basement-membrane disruption and collagen deposition progressively alter tissue stiffness. Individual malignant bladder cells may consequently be softer than normal cells, whereas the extracellular matrix of recurrent tumours may become stiffer because of increased collagen [63,64]. At the tissue scale, matrix stiffening promotes nuclear translocation of Yes-associated protein (YAP) through integrin-focal adhesion kinase (FAK) signalling, Piezo1-mediated calcium influx and cytoskeletal tension. Nuclear YAP induces the connective tissue growth factor (CTGF), alpha-smooth muscle actin (alpha-SMA) and collagen type I alpha 1 chain (*COL1A1*), further increasing collagen deposition and establishing a self-amplifying mechanical feedback loop [41,64,65]. Matrix stiffening also enlarges nuclear pores and increases lamin A/C, facilitating beta-catenin entry and retention in the nucleus and thereby sustaining stemness, invasion and cisplatin tolerance [43]. Conversely, GA-binding protein transcription factor subunit alpha (GABPA) suppresses prolyl 4-hydroxylase subunit alpha 2 (*P4HA2*) through DICER1/microRNA-30e (miR-30e), reducing collagen hydroxylation and YAP1 activation and thereby limiting matrix stiffening and metastasis [66]. Recent studies using collagen–hyaluronan hydrogels with tunable stiffness have provided direct evidence that matrix mechanics modulate metabolic gene expression in bladder cancer spheroids. Metwally et al. demonstrated that HT1376 and T24 spheroids exhibit elevated HK2 expression and glycolytic activity in response to matrix stiffness, with T24 spheroids integrating high glycolytic activity with adhesion remodelling to drive invasion [67]. Dong et al. further reported that mechanical stress in bladder cancer activates YAP/TAZ and c-Myc-associated signalling, upregulating GLUT1, HK2, PFKFB3, and LDHA, and promoting MCT4-mediated lactate export [68]. These findings establish that mechanical cues can directly alter the expression of key metabolic transporters and enzymes in bladder cancer models. Calcium–YAP, Wnt–MYC and SRC signalling can increase proliferative and biosynthetic demand, and collagen hydroxylation consumes proline, alpha-KG, oxygen and ascorbate [34,41,42]. These observations show that mechanical signalling has the capacity to reshape metabolism at the level of transporter and enzyme expression. However, direct measurements of net glucose, glutamine or fatty-acid flux using stable-isotope tracing in controlled-stiffness bladder cancer models remain limited, and some mechanistic connections, particularly for glutamine and fatty-acid metabolism, are extrapolated from other tumour types. Future studies should combine stable-isotope tracing, metabolic imaging and cell-type-resolved omics in models with controlled stiffness and cyclic strain, while incorporating urinary exposure and an intact immune environment, to distinguish mechanical regulation of metabolism from metabolic remodelling of the matrix [41,42,43,63,64,65,66,69]. Such studies are now feasible given the availability of tunable hydrogel systems that recapitulate bladder tumour stiffness and support three-dimensional spheroid culture. The molecular architecture through which driver genes, oncogenic signals, epigenetic modifications and the mechanical microenvironment jointly shape metabolic reprogramming in BC is summarised here (Figure 1).

## 3. Core Metabolic Pathways and Their Interactions

Evidence is interpreted according to the measurement used. Transcript and protein abundance indicate regulatory potential but do not by themselves establish enzyme activity, pathway flux or metabolic dependency. Steady-state metabolite abundance reflects the balance among production, consumption, transport and compartmentation. Stable-isotope tracing can resolve substrate contribution and, when combined with appropriate modelling, estimate pathway flux. Genetic or pharmacological perturbation supports causality within the tested model, while therapeutic dependency requires confirmation in physiologically relevant in vivo systems [70]. A pathway-centred summary of the four major metabolic programmes discussed in this section is provided in Supplementary Figure S1.

### 3.1. Glucose Metabolic Reprogramming

**Figure 2:**
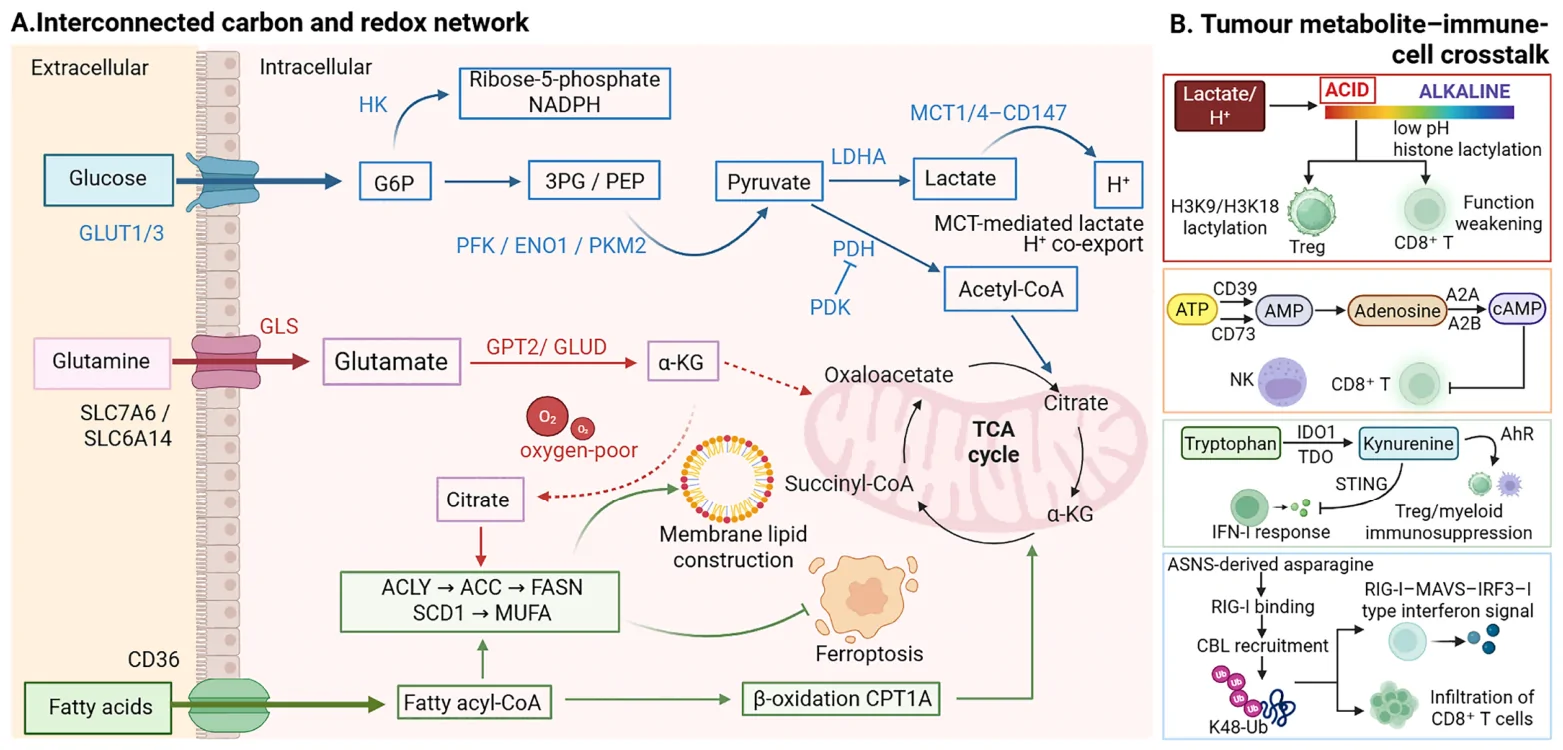
Interconnected metabolic and immunoregulatory networks in bladder cancer. (**A**) Glucose is imported through GLUT1/3 and phosphorylated by hexokinase to glucose-6-phosphate, which is directed either to the pentose phosphate pathway to generate ribose-5-phosphate and NADPH or through PFK, ENO1 and PKM2 to pyruvate. LDHA reduces pyruvate to lactate while regenerating NAD+, and the MCT1/4–CD147 complex exports lactate together with protons. PDK inhibits pyruvate dehydrogenase and restricts pyruvate entry into acetyl-CoA and the TCA cycle. Glutamine imported through SLC7A6/SLC6A14 is converted by GLS to glutamate and then by GPT2 or GLUD to α-ketoglutarate for anaplerosis. Under hypoxia or electron-transport-chain limitation, glutamine-derived α-ketoglutarate can also undergo reductive carboxylation to citrate (dashed arrow). Exogenous fatty acids are imported through CD36, activated and oxidised through CPT1A-dependent β-oxidation to support the TCA cycle. Citrate supplies ACLY–ACC–FASN-dependent de novo fatty-acid synthesis, after which SCD1 generates monounsaturated fatty acids for membrane assembly and protection from ferroptosis. The network emphasises that biomass, reducing power, energy and membrane lipids are sustained by interchangeable carbon sources rather than independent pathways. (**B**) Exported metabolites transmit the tumour metabolic state to the immune microenvironment. Lactate and low pH promote H3K9/H3K18 lactylation in regulatory T cells and restrain CD8+ T-cell function. Extracellular ATP is sequentially converted by CD39 and CD73 to adenosine, which activates A2A/A2B receptors, increases cAMP and suppresses CD8+ T cells and natural killer cells. IDO1/TDO converts tryptophan to kynurenine; kynurenine–AhR signalling supports regulatory T-cell and myeloid immunosuppression and can weaken type I interferon responses by promoting CUL4B/RBX1-dependent STING degradation. ASNS-derived asparagine binds RIG-I and facilitates CBL-mediated K48-linked ubiquitination and proteasomal degradation, thereby limiting RIG-I–MAVS–IRF3–type I interferon signalling and CD8+ T-cell recruitment.

After entering BC cells, glucose can generate ATP and supply carbon for nucleotide, amino-acid and lipid synthesis. GLUT1/3 mediate glucose transport across the plasma membrane, and hexokinase 1/2 (HK1/2) phosphorylate glucose to glucose-6-phosphate. This intermediate can enter the PPP to generate ribose-5-phosphate and NADPH or proceed through phosphofructokinase (PFK), ENO1 and PKM2 to form pyruvate. Partitioning of pyruvate between LDHA and pyruvate dehydrogenase (PDH) determines its principal fate: LDHA reduces pyruvate to lactate while regenerating NAD+, whereas PDH directs it into mitochondria as acetyl-CoA. Pyruvate dehydrogenase kinase (PDK) inhibits PDH by phosphorylation and is therefore a major control point between glycolysis and mitochondrial oxidation. At the distal end of glycolysis, PKM2 conformation feeds back on upstream carbon flow. The highly active tetramer promotes phosphoenolpyruvate conversion to pyruvate, whereas less active conformations permit upstream intermediates to accumulate and enter the PPP, serine synthesis and glycerolipid synthesis. PKM2 can also enter the nucleus and participate in STAT3- and HIF-1α-associated transcription [71,72]. *PDK4* is elevated in high-grade BC and resistant cells; acute *PDK4* silencing or dichloroacetate (DCA) restores PDH activity, suppresses xenograft growth and increases cisplatin sensitivity [73]. In xenograft models, combination treatment with DCA and cisplatin dramatically reduced tumour volumes compared with either agent alone, which was attributed to substantial intratumoural necrosis. By contrast, chronic genetic *PDK4* deficiency in the BBN-induced orthotopic model unexpectedly increased tumour volume and advanced tumour stage [74]. This apparent paradox is likely attributable to several non-mutually exclusive mechanisms. First, chronic loss of *PDK4* from the earliest stages of development may trigger compensatory upregulation of other PDK isoforms or alternative metabolic nodes, which are not engaged during acute pharmacological inhibition in adult tissues. Second, the constitutive knockout model removes *PDK4* in all tissues, potentially altering the metabolic and stromal microenvironment in ways that promote tumour progression. Third, *PDK4* expression is largely lost during tumour formation in both humans and mice, suggesting that *PDK4* may function as a tumour suppressor specifically in later-stage tumours [72]. These findings indicate that acute inhibition and long-term genetic loss are not equivalent. The clinical implication is that continuous, systemic *PDK4* inhibition may not be advisable, whereas intermittent or acute-on-chronic dosing strategies may preserve the therapeutic benefits of acute *PDK4* inhibition while avoiding compensatory adaptations. Clinical precedent supports intermittent DCA dosing (e.g., 15–20 mg/kg orally for 14 days followed by a 7-day break, or 20 mg/kg intravenously twice weekly), and prospective trials are needed to determine the optimal schedule for *PDK4*-targeted therapies in bladder cancer. Hypoxia further diverts pyruvate towards lactate. HIF-1α and JMJD1A jointly induce *GLUT1*, *HK2*, *PGK1*, *LDHA* and *MCT4* [54], and steroid receptor coactivator 3 (SRC-3) is recruited by HIF-1α to promoters including *GLUT1* and *PGK1*. HK2 or LDHA inhibition markedly attenuates SRC-3-driven tumour growth [75]. Circular RNA ST6GALNAC6 (circST6GALNAC6) reduces HK1 stability and glycolysis through the parkin RBR E3 ubiquitin protein ligase (PRKN)-HK1 axis [76], whereas HK2 entering the nucleus under high-glucose conditions forms a transcriptional complex with MYC [20,22]. Glycolytic enzymes therefore govern both carbon flux and transcription through regulated protein stability and nuclear functions (Figure 2A).

Glucose reprogramming in BC extends beyond PKM2. Proteasome 26S subunit, non-ATPase 11 (PSMD11) binds phosphoglucomutase 3 (PGM3) and competitively blocks Parkin-mediated ubiquitination, leading to PGM3 accumulation and simultaneous enhancement of glycolysis and mitochondrial respiration [77]. DLGAP5 stabilises MYC to increase glycolysis and promote gemcitabine resistance [19], while the E2F1-*SLC16A1-AS1* axis maintains glycolysis, OXPHOS and fatty-acid oxidation in parallel [48]. These mechanisms show that proteostasis and transcriptional complexes sustain multiple energy pathways rather than fixing BC cells in a purely glycolytic state. Diversion of glucose intermediates also contributes to resistance. *PHGDH* upregulation in gemcitabine-resistant cells channels more 3-phosphoglycerate into serine and one-carbon metabolism, supplying nucleotide synthesis, reducing power and methyl donors. Increased IDH2 activity and fumarate accumulation inhibit prolyl hydroxylase domain-containing protein 2 (PHD2) and stabilise HIF-1α, while the PHGDH inhibitor NCT-503 combined with erdafitinib enhances tumour suppression in vitro and in vivo [16]. A separate resistant model relies predominantly on FASN-mediated lipogenesis [35], and cisplatin-resistant cells may simultaneously increase OXPHOS, fatty-acid metabolism and antioxidant defence [21].

MCT1 and MCT4 mediate transmembrane transport of lactate and protons. CD147 associates with both transporters and promotes their localisation at the plasma membrane, enabling efficient lactate-proton symport [78]. This process prevents sustained intracellular acid loading and permits the LDHA reaction to maintain glycolysis through NAD+ regeneration. In BC tissue, high MCT1 expression is associated with high-grade, advanced stage and lymphatic invasion, whereas MCT4 is associated with recurrence [79]. Exported lactate and protons jointly create a low-glucose, low-pH microenvironment, and lactate can further alter transcriptional programmes in tumour and immune cells through H3K18la [57,58]. High lactate output does not, however, imply the loss of mitochondrial function. E2F1–*SLC16A1-AS1*, PSMD11–PGM3 and the cisplatin-induced switch towards OXPHOS all support hybrid energy metabolism in BC [6,21,48,77]. Deuterated-glucose metabolic imaging tracked labelled lactate and water in vitro and in subcutaneous and orthotopic models, detecting a chemotherapy-induced reduction in glycolysis before morphological tumour changes became apparent [80]. The principal drivers, core pathways, functional outputs and candidate targets of glucose metabolism are summarised in Supplementary Figure S1A.

### 3.2. Lipid Metabolic Reprogramming

Lipids provide BC cells with membrane structure, lipid-raft signalling and energy reserves, and their fatty-acid composition directly influences lipotoxicity and ferroptosis sensitivity. De novo fatty-acid synthesis begins when ATP citrate lyase (ACLY) converts mitochondria-derived citrate to acetyl-CoA; acetyl-CoA carboxylase (ACC) generates malonyl-CoA; FASN synthesises palmitate; and SCD1 desaturates saturated fatty acids to monounsaturated fatty acids. FGFR3 activates SREBP1 and induces *SCD1* through PI3K–mTORC1 [15], PKM2 increases FASN through the AKT–mTOR–SREBP-1c axis [81], and *PIK3CA* E545K uses glutamine reductive carboxylation to generate citrate, showing that lipogenic carbon can derive from either glucose or glutamine [51]. Incorporation of SCD1-derived monounsaturated fatty acids into membrane phospholipids lowers the proportion of readily oxidised polyunsaturated fatty acids and limits ferroptosis. The LRP8-Wnt–β-catenin-SCD1 axis maintains this phenotype at the receptor-signalling level [40], whereas NOP2-mediated m5C modification and YBX1 recognition increase *SCD* expression through RNA stabilisation. NOP2 inhibition reduces monounsaturated fatty acids, increases lipid ROS and induces ferroptosis [56]. H3K18la can additionally increase *SREBF1* and *SLC7A11*; the former promotes lipogenesis, while the latter maintains cysteine uptake and GSH/glutathione peroxidase 4 (GPX4)-dependent antioxidant defence [57]. Ferroptosis sensitivity therefore depends on the balance of polyunsaturated and monounsaturated fatty acids in membrane phospholipids, phospholipid remodelling, iron load and reducing systems rather than total lipid abundance [44,45,57,82]. BC cells can also take up circulating lipoproteins and mobilise adjacent adipose tissue. Hypercholesterolaemia induced by diet or *Ldlr* loss enhances tumour stemness, with mechanistic studies identifying oxidised low-density lipoprotein (ox-LDL) as the relevant mediator. Ox-LDL binds CD36, recruits Janus kinase 2 (JAK2) and activates signal transducer and activator of transcription 3 (STAT3); *CD36* deletion, a binding-deficient mutation or a neutralising antibody reverses the stem-like phenotype [83]. Intracellular cholesterol homeostasis is likewise important. UbiA prenyltransferase domain-containing protein 1 (*UBIAD1*/*TERE1*) is reduced in some invasive BCs, and restoring its expression lowers cellular cholesterol and suppresses J82 xenograft growth [84]. When tumours invade perivesical fat, BC cells secrete parathyroid hormone-related protein (PTHrP) and activate adipocyte PTH receptor-protein kinase A (PTHR–PKA) signalling, inducing the browning of white adipose tissue and hormone-sensitive lipase (HSL)-associated lipolysis. Tumour uptake of the released fatty acids upregulates *CD36*, carnitine palmitoyltransferase 1A (*CPT1A*) and PPAR-related programmes, whereas PKA inhibition reverses adipose browning and slows orthotopic tumour progression [85]. This tumour–adipocyte circuit provides a mechanistic explanation for the association between perivesical-fat invasion at pT3 and poor prognosis and suggests that exogenous lipid uptake may weaken FASN monotherapy. Fatty-acid oxidation is not uniformly tumour-promoting. DNA methyltransferase 1 (DNMT1)-associated methylation reduces acyl-CoA synthetase long-chain family member 5 (*ACSL5*) expression in BC. Restoring *ACSL5* increases long-chain-fatty-acid activation, fatty-acid oxidation and acetyl-CoA, which promotes p53-binding protein 1 (53BP1) K1360 acetylation and recruits p53–p21, ultimately inducing senescence and tumour suppression [86]. Fatty-acid-derived acetyl-CoA can therefore have divergent outcomes depending on whether it enters protein acetylation or the TCA cycle. In another mitochondrial branch, YTH N6-methyladenosine RNA binding protein 2 (YTHDF2) promotes m6A-dependent degradation of lipoyltransferase 1 (*LIPT1*) mRNA. *LIPT1* restoration increases lipoylation and aggregation of TCA-cycle proteins, triggering cuproptosis while reducing lipid droplets and increasing ER stress, thereby suppressing BC growth [87]. The corresponding features of lipid and cholesterol metabolism are summarised in Supplementary Figure S1B.

### 3.3. Amino-Acid Metabolic Reprogramming

Amino acids supply anaplerotic carbon and biosynthetic nitrogen, support GSH synthesis and regulate immunity in BC; glutamine links these functions. After uptake through solute carrier (SLC) transporters, glutamine is converted by glutaminase (GLS) to glutamate and then to alpha-KG through glutamic-pyruvic transaminase 2 (GPT2)-mediated transamination or glutamate dehydrogenase (GLUD)-mediated oxidative deamination. When mitochondrial function is intact, alpha-KG enters the oxidative TCA cycle. Under hypoxia or electron-transport-chain limitation, glutamine-derived alpha-KG can instead undergo IDH1/2-dependent reductive carboxylation to citrate and supply carbon for lipid synthesis [51,88]. BC cells reinforce this pathway through uptake, transamination and signalling feedback. ROS generated during glutaminolysis can activate STAT3 and promote proliferation; ROS scavenging or STAT3 inhibition attenuates this effect [89]. The lncRNA urothelial cancer associated 1 (*UCA1*) binds heterogeneous nuclear ribonucleoproteins I/L (hnRNP I/L) and increases *GPT2* expression, thereby converting glutamate more efficiently to alpha-KG and replenishing the TCA cycle [90]. Runt-related transcription factor 2 (RUNX2) binds directly to the *SLC7A6* promoter and increases transcription, enhancing glutamine supply at the uptake step; *SLC7A6* knockdown weakens RUNX2-driven proliferation and invasion [91]. The hypoxia-induced PYCR1–lactate–H3K18la–SLC6A14 axis further increases glutamine uptake through epigenetic feedback [58]. Glutamine dependency is therefore determined collectively by transport, transamination, oxidative catabolism and reductive carboxylation rather than by GLS activity alone; inhibiting one node may also provoke compensatory use of serine, one-carbon metabolism or alternative mitochondrial substrates [92].

Arginine and proline metabolism illustrate distinct nutrient dependencies. BC cells lacking argininosuccinate synthase 1 (*ASS1*) cannot efficiently resynthesise arginine from citrulline and therefore depend more strongly on circulating arginine. Pegylated arginine deiminase (ADI-PEG20) degrades circulating arginine and preferentially suppresses *ASS1*-low BC [93]. Evidence that arginine deprivation induces compensatory glutamine, serine and mitochondrial metabolism comes mainly from studies across tumour types [92] and requires confirmation in longitudinal BC samples. In the proline branch, PYCR1 reduces pyrroline-5-carboxylate (P5C) to proline while consuming NAD(P)H, thereby linking the proline cycle, redox balance and collagen synthesis. In BC, PYCR1 promotes tumour growth through AKT-Wnt–β-catenin signalling, and SUMO-specific peptidase 3 (SENP3)-mediated deSUMOylation of STAT3 increases *PYCR1* transcription [94,95]. Hypoxia-induced PYCR1 also raises lactate and H3K18la, upregulates *SLC6A14* and further couples proline, glucose and glutamine metabolism. Three PYCR isoforms exist (PYCR1, PYCR2, and PYCR3/PYCRL), with PYCR1 and PYCR2 being mitochondrial and PYCR3 cytosolic. Direct evidence for compensatory upregulation of PYCR2 or PYCR3 upon PYCR1 inhibition in bladder cancer is lacking, although such compensation has been observed in other systems. PYCR1 is also highly expressed in cancer-associated fibroblasts (CAFs), where it supports proline synthesis for collagen deposition and extracellular matrix remodelling [96]. The therapeutic selectivity of PYCR1 therefore requires validation in models that distinguish tumour cells from stromal and normal cells [58,94,95].

Asparagine and tryptophan metabolism influence treatment responses in BC by altering innate immune sensing and T-cell function. Asparagine synthetase (ASNS)-derived asparagine binds directly to retinoic acid-inducible gene I (RIG-I). This interaction promotes Cbl proto-oncogene (CBL) E3 ligase-mediated K48 ubiquitination and proteasomal degradation of RIG-I, thereby weakening the RIG-I–mitochondrial antiviral-signalling protein (MAVS)–interferon regulatory factor 3 (IRF3) axis and type I interferon output [97]. This mechanism primarily affects the immune ecosystem rather than basal proliferation: *ASNS* knockdown has little effect on proliferation in vitro but suppresses tumours in immunocompetent models through a CD8-positive (CD8+) T-cell-dependent mechanism. L-asparaginase synergises with anti-PD-1 therapy in subcutaneous and orthotopic models, and high *ASNS* expression is associated with poor responses to immune-checkpoint inhibitors (ICIs) [97]. Patients with BC have lower plasma tryptophan and a higher circulating kynurenine-to-tryptophan ratio, which correlates with tumour burden [98]. This concentration-based association reflects systemic tryptophan catabolism rather than a direct measurement of intratumoural metabolic flux. The circulating ratio is influenced by hepatic TDO activity, immune activation, renal handling and substrate availability; inference of tumour-local IDO1/TDO activity therefore requires tissue-based or functional validation [99]. Paired plasma and tissue or urine samples are needed to distinguish tumour-derived kynurenine from systemically produced kynurenine and to determine whether blood metabolite changes originate from the tumour microenvironment. Mechanistically, within the tumour microenvironment, conversion of tryptophan to kynurenine by IDO1 or TDO can deplete local tryptophan and activate GCN2 nutrient stress, whereas kynurenine activates AhR signalling to promote Treg and myeloid immunosuppression [92,98]. In tumour cells, AhR also recruits STING to the CUL4B/RBX1 E3 ligase complex and promotes its degradation, weakening chemotherapy-induced type I interferon responses [100]. The corresponding features of amino-acid metabolism are summarised in Supplementary Figure S1C.

### 3.4. Mitochondrial Metabolism

The Warburg effect describes increased lactate production under aerobic conditions, but it does not imply complete loss of mitochondrial function. BC cells can redistribute pyruvate, glutamine and fatty acids between glycolysis and OXPHOS according to oxygen availability, substrate supply and treatment pressure [6,21,51]. This plasticity can emerge rapidly after cisplatin exposure. Real-time metabolic imaging showed that a subset of cells switches to high OXPHOS before stable resistant clones develop, and the complex I inhibitor phenformin attenuates this short-term tolerant state [6]. Long-term resistance further depends on mitochondrial translation and quality control. Circular RNA FOXK2 (circFOXK2) acts as a molecular scaffold that strengthens the interaction between heat shock protein 90 beta family member 1 (HSP90beta) and translational activator of cytochrome c oxidase I (TACO1) and promotes TACO1 mitochondrial translocation. TACO1 then increases mitochondrially encoded cytochrome c oxidase I (MTCO1) translation, complex IV activity and OXPHOS. The moderate mitochondrial ROS generated through this programme helps maintain tumour stemness and cisplatin tolerance [101]. In parallel, calcium-binding protein 39 (CAB39) activates liver kinase B1 (LKB1)–AMP-activated protein kinase (AMPK) signalling and promotes microtubule-associated protein 1 light chain 3 (LC3)-related autophagy, removing cisplatin-damaged mitochondria and limiting excessive ROS [102]. Serine and arginine rich splicing factor 1 (SRSF1)-mediated alternative splicing of *HIF1A* increases BCL2 interacting protein 3 (BNIP3)-dependent mitophagy and likewise reduces cisplatin sensitivity [103]. The first pathway increases electron transport and ATP generation, while the latter two preserve mitochondrial quality; together, they constrain ROS to a range compatible with signalling but insufficient to trigger cell death. Patient-relevant models also support coupling between substrate supply and the respiratory chain. NADH: ubiquinone oxidoreductase subunit B8 (*NDUFB8*) knockdown reduces complex I function, ATP, GLS1 and proliferation [104], whereas *PIK3CA* E545K increases both complex I activity and glutamine carbon flux [51]. Mesenchymal stem cells can additionally regulate mitochondrial dynamics and promote tumour progression through kynurenine–AMPK signalling [105]. Mitochondrial adaptation does not invariably increase OXPHOS. Growth arrest and DNA damage-inducible beta (*GADD45B*) downregulation can switch BC from OXPHOS to glycolysis [106]; voltage-dependent anion channel 1 (*VDAC1*) silencing reduces GLUT1, HK, citrate synthase, complex IV and ATP synthase [107]; and TRIM21-mediated degradation of YME1-like 1 ATPase (YME1L1) disrupts mitochondrial proteostasis and suppresses energy production [108]. Accordingly, increases or decreases in oxygen consumption cannot be interpreted without reference to disease stage, molecular background and treatment pressure [21,106,107,108,109,110]. The corresponding features of mitochondrial metabolism are summarised in Supplementary Figure S1D.

### 3.5. Metabolite-Mediated Immunosuppression

BC-derived metabolites reshape local immunity through nutrient competition, receptor signalling and epigenetic modification, with the most direct evidence relating to lactate. BC cells convert pyruvate to lactate through LDHA and export lactate with protons through the MCT1/MCT4-CD147 complex. This relieves intracellular acid stress but simultaneously creates a low-glucose, low-pH local environment [78,79,111]. In *KDM6A*-deficient BC models, reduced tumour lactate lowers H3K9/H3K18 lactylation and *FOXP3*, TGF-β and PD-1 expression in Treg cells, improving the response to anti-PD-1 therapy [31]. Lactate can also activate STAT3 and induce lipocalin 2 (LCN2); reducing lactate or silencing *LCN2* suppresses epithelial–mesenchymal transition, spheroid formation and gemcitabine resistance [112]. The adenosine pathway converts the ATP released during tissue injury into receptor-mediated immunosuppression. Extracellular ATP is hydrolysed first by CD39 to adenosine monophosphate (AMP) and then by CD73 to adenosine. Adenosine engages A2A/A2B receptors, increases cyclic adenosine monophosphate (cAMP) in immune cells, weakens CD8+ T-cell and natural killer (NK)-cell function and supports Treg maintenance. CD73 deficiency increases CD8+ T-cell cytotoxicity and antitumour activity, supporting a causal role for this metabolite-receptor chain [113]. In BC specimens, infiltration by CD73-positive cytotoxic T cells and Treg cells is associated with invasion and poor prognosis, and some cells in highly invasive tumours co-express PD-1 [114]. Paired peripheral-blood and tumour analyses further show that CD39/CD73-positive T cells accompany Treg cells, M2-like macrophages and low-effector T-cell states, and that some circulating subsets reflect the intratumour immune composition [115]. CD39/CD73 expression indicates the capacity to generate adenosine, but actual exposure also depends on ATP release, adenosine deaminase, nucleoside transport and diffusion distance; purine-metabolism kinetics likewise vary among BC cells [114,115,116] (Figure 2B).

Tryptophan and asparagine metabolism influence immunotherapy at the level of innate immune sensing. IDO1/TDO-mediated tryptophan depletion activates GCN2 and inhibits mTOR, while accumulated kynurenine binds AhR and promotes Treg and myeloid immunosuppression [98,117]. In BC, AhR can recruit STING to the CUL4B/RBX1 E3 ligase complex and promote its ubiquitination and degradation, weakening chemotherapy-induced type I interferon responses. AhR blockade or STING restoration enhances chemotherapy-associated immune activation [100]. Asparagine acts through a distinct RNA-sensing pathway. ASNS-derived asparagine binds RIG-I and promotes CBL-mediated K48 ubiquitination and degradation. Restricting ASNS activity or using L-asparaginase can restore RIG-I-IRF3-type I interferon signalling, increase CD8+ T-cell infiltration and enhance anti-PD-1 activity [97]. In lipid metabolism, the PPARγ-driven luminal state promotes immune exclusion by suppressing NF-κB-related inflammation [62], whereas TRIM28-stabilised SREBF1 enhances programmed death-ligand 1 (PD-L1) N-glycosylation and membrane localisation through mannosyl-glycoprotein N-acetylglucosaminyltransferase 4A (MGAT4A) [118]. These mechanisms nominate testable biomarkers for pairing metabolic therapies with immunotherapy, including lactate–Treg lactylation, ASNS–RIG-I-type I interferon, AhR–STING and SREBF1–PD-L1 glycosylation. Conversely, adding metabolic inhibitors indiscriminately to the PD-1/PD-L1 blockade without evidence of pathway activity obscures pharmacodynamic attribution and may compromise the metabolic requirements of effector T cells [31,97,100,119,120,121,122,123,124]. Together, the glucose, glutamine and fatty-acid networks and their metabolite-mediated immune effects form an interconnected carbon and immunoregulatory system (Figure 2A,B).

Across these studies, non-coding RNAs regulate metabolic enzyme expression, protein stability, mitochondrial translation and metabolite-dependent immunity. Table 1 compares the reported molecular targets, mechanisms and biological outcomes. Most links remain based on preclinical bladder cancer models; the proposed AC107027.3–miR-27A-3p–FASN pathway is computational and requires functional validation [48,76,90,101,125,126,127,128].

## 4. Metabolic Adaptation and Treatment Resistance

Although cisplatin- and gemcitabine-based chemotherapy and ICIs have substantially improved outcomes for some patients with BC, primary and acquired resistance prevents most patients with advanced disease from achieving durable benefit [3,4]. Recent studies indicate that, beyond genetic mutation and clonal selection, metabolic reprogramming provides tumour cells with an earlier and more flexible adaptive mechanism [129,130,131]. Unlike genetic alterations, metabolic adaptations can be initiated within hours of drug exposure and do not require new mutations, allowing BC cells to gain an immediate survival advantage. Chemoresistance is therefore a treatment-driven evolutionary process: early phases are dominated by non-genetic plasticity and metabolic reorganisation, whereas subsequent signalling consolidation and microenvironmental remodelling stabilise the resistant phenotype [131,132]. Metabolic reprogramming supplies energy and biosynthetic precursors to resistant cells and also alters redox balance, DNA-repair efficiency and the immune microenvironment, making it central to both chemotherapy and immunotherapy resistance.

### 4.1. Metabolic Reprogramming in Chemoresistance

#### 4.1.1. Mitochondrial Reprogramming and Cisplatin Resistance

Cisplatin cytotoxicity arises mainly from platinum-DNA adducts that stall DNA replication and induce double-strand breaks. Repairing this damage requires substantial ATP, and rapid upregulation of mitochondrial OXPHOS is an early adaptive strategy in BC cells. Real-time metabolic imaging identified a key phenomenon: during the initial phase of cisplatin exposure, before stable resistant clones formed, a subset of T24 cells rapidly adopted a high-OXPHOS state. The complex I inhibitor phenformin attenuated this short-term tolerant phenotype. Rapidly increased mitochondrial respiration therefore acts as an early metabolic buffer against platinum damage rather than a compensation acquired only after long-term adaptation [4]. In established resistance, the circFOXK2–HSP90β–TACO1 axis enhances mitochondrial translation, complex IV activity and OXPHOS, thereby sustaining tumour stemness and cisplatin tolerance [129].

Resistant BC cells also preserve mitochondrial quality by increasing autophagy. CAB39 activates the LKB1–AMPK–LC3 pathway, promotes removal of cisplatin-damaged mitochondria and limits excessive ROS, thereby maintaining energy supply while avoiding oxidative injury [130]. SRSF1-mediated alternative splicing of *HIF1A* increases BNIP3-dependent mitophagy and reduces cisplatin sensitivity [131]. Enhanced electron transport, autophagic clearance of damaged organelles and mitochondrial quality control collectively constrain ROS to the narrow range that supports signalling without triggering cell death, establishing a coordinated strategy that couples energy production to organelle preservation [129,130,131].

The molecular basis of mitochondrial adaptation varies across genetic backgrounds. *NDUFB8* knockdown reduces complex I function, ATP production and GLS1 expression, indicating close coupling between electron transport and glutamine metabolism [104]. *PIK3CA* E545K simultaneously increases complex I activity and glutamine carbon flux, making glutamine an important substrate for OXPHOS [51]. Mitochondrial adaptation does not, however, increase OXPHOS in every BC context. *GADD45B* downregulation can shift BC from OXPHOS to glycolysis [106]; *VDAC1* silencing reduces GLUT1, HK, citrate synthase and ATP synthase expression [107]; and TRIM21-mediated YME1L1 degradation disrupts mitochondrial proteostasis and suppresses energy generation [108]. These divergent findings underscore that either increased or decreased OXPHOS must be interpreted in the context of disease stage, molecular background and treatment pressure. Mitochondrial plasticity itself, rather than a single respiratory state, is a core component of the adaptive repertoire of resistant cells.

#### 4.1.2. Glycolytic Reprogramming and Chemoresistance

In parallel with mitochondrial adaptation, resistant BC cells commonly increase glycolytic flux through several upstream signals. SRC is a key node connecting glycolytic reprogramming to cisplatin resistance. In cisplatin-resistant cells, *SRC* overexpression activates HK2 and increases glycolysis, particularly the PPP. The PPP generates ribose-5-phosphate for nucleotide synthesis and repair of platinum-DNA adducts while producing NADPH to neutralise cisplatin-induced ROS, thereby protecting tumour cells from DNA damage. *SRC* deletion or the SRC inhibitor eCF506 reverses these effects and increases cisplatin sensitivity in both cell-derived xenograft (CDX) and patient-derived xenograft (PDX) models [133]. This work links non-receptor tyrosine-kinase signalling, glucose-metabolic reprogramming and chemoresistance and provides a rationale for co-targeting SRC and glycolysis.

MYC-stabilising pathways also contribute to chemoresistance. In BC models, the DLGAP5–USP11–MYC loop promotes gemcitabine resistance, whereas USP43–c-MYC signalling increases LDHA-dependent glycolysis [19,24]. Redundant regulation of MYC stability may limit the effect of inhibiting a single deubiquitinase.

Cyclin-dependent kinase inhibitor 3 (CDKN3) defines another pathway linking glycolysis to chemoresistance. CDKN3 upregulates *LDHA*, enhances glycolysis and drives cisplatin resistance. *CDKN3* knockdown reverses both increased glycolysis and chemoresistance, whereas *LDHA* overexpression partially rescues the phenotype caused by *CDKN3* depletion [111]. This direct connection among a cell-cycle regulator, a glycolytic enzyme and chemoresistance identifies LDHA as a principal downstream effector of CDKN3 in resistant BC.

Enhanced glycolysis also involves diversion of metabolic intermediates and activation of bypass pathways. *PHGDH* upregulation in gemcitabine-resistant cells directs more 3-phosphoglycerate into serine and one-carbon metabolism, supplying nucleotide synthesis. In parallel, the gain of IDH2 function stabilises HIF-1α through reductive glutamine metabolism and stimulates aerobic glycolysis and the PPP [16,134]. Fumarate accumulation further stabilises HIF-1α by inhibiting PHD2, creating a metabolite-signalling positive feedback loop [16].

#### 4.1.3. Lipid Metabolic Reprogramming and Chemoresistance

Dysregulated lipid metabolism is another major contributor to chemoresistance in BC. Cisplatin-resistant cells display markedly increased lipogenic capacity: relative to sensitive cells, they consume more glucose and produce more glucose-derived acetate and fatty acids. Isotope tracing further identifies acyl-CoA synthetase short-chain family member 2 (ACSS2) as the key enzyme that supplies acetyl-CoA from endogenous, glucose-derived acetate in resistant cells, indicating a preference for glucose rather than exogenous acetate as lipogenic carbon [135]. Targeting the ACSS2-acetate-lipid axis may therefore help overcome cisplatin resistance. Gemcitabine resistance also involves lipid remodelling. An RNA sequencing-derived resistance-metabolism risk score associates high risk with more active lipid synthesis. *FASN* was identified as a representative driver of gemcitabine resistance, and the FASN inhibitor TVB-3166 reverses resistance in vitro and in vivo [35]. Consistently, FASN is a central regulator of fatty-acid metabolism in BC and correlates with immune-cell infiltration, while patients with low *FASN* expression respond better to ICI therapy [126].

Cholesterol remodelling also contributes to chemoresistance. Sterol O-acyltransferase 1 (SOAT1; historically termed acyl-CoA:cholesterol acyltransferase 1 or ACAT1) catalyses cholesterol esterification and is highly expressed in BC tissue, where it is positively associated with tumour grade. The SOAT inhibitor avasimibe activates PPARγ signalling, suppresses BC-cell proliferation and migration, induces G1 cell-cycle arrest and increases ROS [136]. Ox-LDL enhances tumour stemness through the CD36-JAK2–STAT3 axis, and serum ox-LDL predicts poor prognosis [83]. As a key cholesterol synthesis enzyme, 7-dehydrocholesterol reductase (DHCR7) is upregulated in BC and promotes metastasis through cyclic AMP–PKA–FAK signalling. DHCR7 inhibition reduces cholesterol synthesis and lipid-raft remodelling and suppresses metastasis [137]. These findings jointly connect cholesterol homeostasis to BC progression and treatment resistance.

#### 4.1.4. Epigenetic-Metabolic Interactions in Chemoresistance

Bidirectional regulation between epigenetic modification and metabolic reprogramming has emerged as an important determinant of chemoresistance. Histone lactylation is a recently recognised mechanism linking glycolysis to epigenetic control. Cisplatin-resistant BC cells display increased H3K18la, and targeted reduction of this modification restores cisplatin sensitivity. The H3K18la-regulated transcription factors YBX1 and Yin Yang 1 (YY1) contribute to resistance [138]. Lactylation also stabilises the m6A reader YTHDF3 and promotes m6A-dependent decay of *KDM6B* mRNA; KDM6B controls *CDKN1A* transcription through H3K27me3 demethylation. The resulting lactate–alanyl-tRNA synthetase 2 (AARS2)–YTHDF3–KDM6B–CDKN1A axis integrates metabolic reprogramming, m6A epitranscriptomic control and chromatin remodelling to drive cisplatin resistance [139]. YTH domain-containing protein 1 (YTHDC1) positively regulates *PTEN* expression in an m6A-dependent manner, and low YTHDC1 expression is associated with reduced cisplatin sensitivity in patients with BC, highlighting the role of m6A modification in the PTEN–PI3K–AKT pathway [140]. m5C modification also contributes to metabolic control of chemoresistance. HIF-1α induces ALYREF, which recognises m5C sites in *PKM2* mRNA and increases transcript stability, sustaining *PKM2* expression and glycolysis in resistant cells [55]. These findings support therapeutic targeting of epigenetic–metabolic interactions to overcome resistance.

#### 4.1.5. Dual Effects of Genomic Instability and Metabolic Remodelling

*KDM6A* loss illustrates the treatment-specific consequences of metabolic remodelling. It promotes cisplatin resistance through impaired DNA repair and extrachromosomal resistance loci, whereas reduced lactate production limits Treg-cell lactylation and improves anti-PD-1 activity in BC models [31]. The same alteration may therefore predict different responses to chemotherapy and immunotherapy.

### 4.2. Metabolic Barriers to Immunotherapy

ICIs are an important treatment option for patients with BC who are ineligible for cisplatin or whose disease progresses after platinum therapy [5,141]. Nevertheless, more than half of patients do not benefit from ICI monotherapy, and acquired resistance can develop even after an initial response [142]. Metabolic reprogramming is central to the establishment of an immunosuppressive tumour microenvironment (TME) and immunotherapy resistance in BC. The relevant mechanisms include lactate-driven immunosuppression, lipid-mediated immune escape and crosstalk between metabolites and immune checkpoints.

#### 4.2.1. Lactate-Driven Immunosuppression

BC cells convert pyruvate to lactate through LDHA and export lactate with protons through the MCT1/MCT4–CD147 complex, creating a low-glucose, low-pH metabolic microenvironment [78,79]. Lactate affects immune cells through several mechanisms. Its effects on Treg cells are particularly complex. In highly glycolytic tumour microenvironments, including *MYC*-amplified tumours, Treg cells actively import lactate through MCT1, promoting the nuclear translocation of nuclear factor of activated T cells 1 (NFAT1) and increasing PD-1 expression, whereas PD-1 expression in effector T cells is suppressed [143]. PD-1 blockade may therefore preferentially activate PD-1-high Treg cells rather than effector T cells, weakening therapeutic efficacy. Lactate also signals through G protein-coupled receptor 81 (GPR81) to induce FOXP3 and cytotoxic T-lymphocyte-associated protein 4 (CTLA-4) in Treg cells and promotes transcription of immunosuppressive genes, including interleukin 10 (IL-10) and TGF-β, through epigenetic modification.

In macrophages, lactate can promote M2 polarisation. Interleukin 4 (IL-4)-induced conversion of M0 to M2 macrophages is characterised by glucose- or lactate-dependent TCA-cycle metabolism that directly drives histone acetylation, M2-gene transcription and functional immunosuppression. Lactate-dependent M2 polarisation requires mitochondrial pyruvate uptake and ACLY activity; exogenous acetate rescues defective M2 polarisation caused by mitochondrial pyruvate carrier 1 (MPC1) inhibition or ACLY deficiency [144]. In BC, tumour-associated macrophages (TAMs) enhance PD-L1-mediated immune escape through nuclear translocation of a PKM2 dimer–STAT3 complex, a process driven in part by TGF-β secreted from M2-like TAMs [145].

Excessive glucose uptake by tumour cells lowers local glucose to very low concentrations, forcing CD8+ T cells to rely on fatty-acid oxidation. Their limited mitochondrial capacity ultimately leads to functional exhaustion [146]. In BC cells, circular RNA FAM13B (circFAM13B) suppresses glycolysis through the insulin-like growth factor 2 mRNA-binding protein 1 (IGF2BP1)–PKM2 pathway, reverses TME acidification and increases sensitivity to PD-1 antibodies [125]. This mechanism provides direct evidence that targeting glycolysis can sensitise BC to immunotherapy.

Histone lactylation provides an epigenetic link between lactate production and immune escape. BC models associate LDHA-dependent H3K18la or H4K5la with tumour growth and PD-L1 expression, whereas reduced lactate in *KDM6A*-deficient tumours limits Treg-cell lactylation and improves anti-PD-1 activity [31,52,147,148]. These findings remain preclinical.

#### 4.2.2. Lipid-Mediated Immune Escape

Dysregulated lipid metabolism contributes to immunotherapy resistance through several routes. Differential *FASN* expression is strongly associated with immune-cell infiltration, and patients with low *FASN* expression respond better to ICI therapy. SREBF1 is predicted to be the principal *FASN* transcription factor, while lncRNA *AC107027.3* may increase *FASN* through competitive binding to microRNA-27A-3p (miR-27A-3p), thereby regulating the immunotherapy response [126]. In BC, circular RNA ZNF609 (circZNF609) increases fatty-acid uptake through the IGF2BP2-CD36 pathway, depletes fatty acids from the TME and reduces immunotherapy sensitivity [127]. This finding directly links a circular RNA, fatty-acid metabolism and immune escape. Cholesterol also regulates T-cell function. Cholesterol accumulation in the TME induces ER stress and XBP1 activation and upregulates PD-1, CD244 (2B4), T-cell immunoglobulin and mucin domain-containing protein 3 (TIM-3) and lymphocyte-activation gene 3 (LAG-3) on CD8+ T cells, promoting exhaustion [149]. In BC, 25-hydroxycholesterol is increased and promotes proliferation and epithelial–mesenchymal transition [150]. Ox-LDL enhances tumour stemness through CD36-JAK2–STAT3, and serum ox-LDL predicts poor prognosis [83]. These lipid-related mechanisms provide a rationale for combining FASN inhibitors with ICIs.

#### 4.2.3. Metabolite-Immune-Checkpoint Crosstalk

Metabolic and stromal signals can directly regulate immune-checkpoint expression. Cancer-associated fibroblast (CAF)-derived C-X-C motif chemokine ligand 12 (CXCL12) links stromal signalling to immune escape in BC. CXCL12 activates JAK2–STAT3, upregulates the deubiquitinase cylindromatosis (CYLD) and causes sequestosome 1 (p62) accumulation; p62 then inhibits autophagic degradation of PD-L1 and enhances immune escape [151]. This cascade connects a fibroblast-derived factor, proteostasis and PD-L1 abundance. Ras GTPase-activating protein-binding protein 1 (G3BP1) and splicing factor SLU7 form a complex that stabilises closed-loop mRNA structures of class IA PI3Ks, increasing translation and stability, activating PI3K–AKT and downregulating the major histocompatibility complex class I (MHC-I). Epigallocatechin gallate (EGCG), which targets G3BP1, impairs immune escape and sensitises tumours to anti-PD-1 therapy [152]. TRIM28-mediated SUMOylation of SREBF1 drives PD-L1 N-glycosylation and membrane localisation, directly coupling lipid regulation to immune-checkpoint surface stability [118]. PKM2 also promotes immune escape by increasing PD-L1 expression, with TAM-derived TGF-β acting as an important upstream driver [145].

#### 4.2.4. Metabolite Sensing and Innate Immune Regulation

Amino-acid metabolites influence immunotherapy by altering innate immune sensing. ASNS-derived asparagine binds RIG-I and promotes CBL E3-ligase-mediated K48 ubiquitination and RIG-I degradation, weakening the RIG-I–MAVS–IRF3 axis and type I interferon output. L-asparaginase synergises with anti-PD-1 therapy in subcutaneous and orthotopic models, and high *ASNS* expression is associated with poor ICI responses [97]. Tryptophan metabolism acts through the kynurenine–AhR axis. IDO1/TDO-mediated tryptophan depletion activates GCN2 nutrient stress, while accumulated kynurenine binds AhR to promote Treg and myeloid immunosuppression. In BC, AhR also recruits STING to the CUL4B/RBX1 E3 ligase complex and promotes its ubiquitination and degradation, thereby weakening chemotherapy-induced type I interferon responses [100]. The adenosine pathway converts ATP released during tissue injury into receptor-mediated immunosuppression. Extracellular ATP is hydrolysed by CD39 to AMP and then by CD73 to adenosine. Engagement of A2A/A2B receptors increases cAMP in immune cells, weakens CD8+ T-cell and NK-cell activity and supports Treg maintenance. In BC specimens, infiltration by CD73-positive cytotoxic T cells and Treg cells is associated with tumour invasion and poor prognosis [114,115].

**Figure 3:**
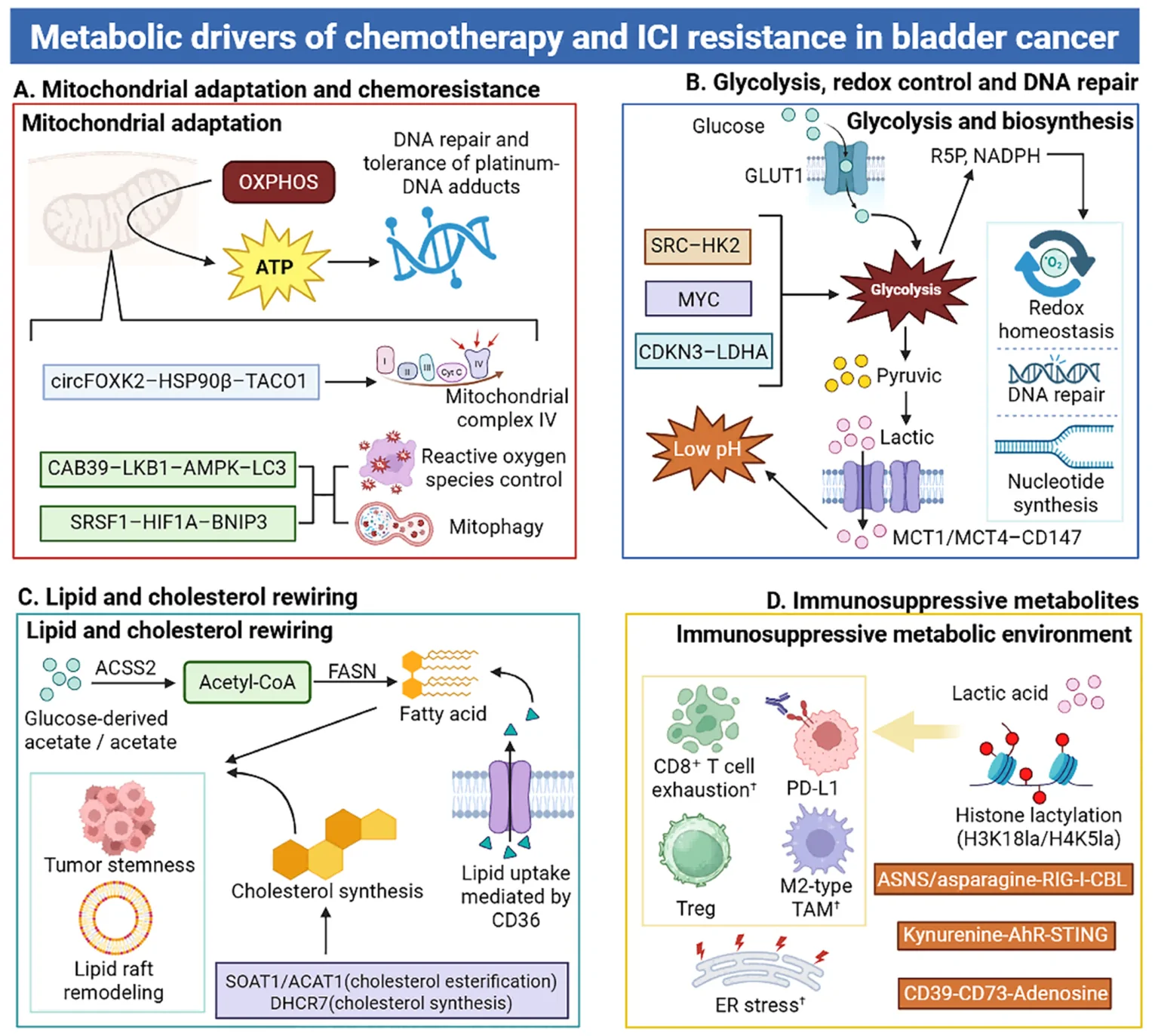
Metabolic reprogramming coordinately drives chemotherapy and immune-checkpoint-inhibitor resistance in bladder cancer. Mitochondrial adaptation increases oxidative phosphorylation (OXPHOS) and ATP production to support repair of platinum-DNA adducts. The circFOXK2–HSP90beta–TACO1 axis promotes MTCO1 translation and complex IV activity, whereas CAB39–LKB1–AMPK–LC3- and SRSF1–HIF1A–BNIP3-dependent mitophagy removes damaged mitochondria and maintains reactive oxygen species within a signalling-compatible range. SRC–HK2, MYC and CDKN3–LDHA signalling enhance glycolysis and divert carbon into the pentose phosphate, serine and one-carbon pathways. Ribose-5-phosphate and NADPH thereby sustain nucleotide synthesis, DNA repair and redox homeostasis. Lactate export through MCT1/MCT4-CD147 establishes an acidic microenvironment. The ACSS2-acetyl-CoA–FASN axis, SOAT1-dependent cholesterol synthesis and CD36-dependent lipid uptake promote fatty-acid synthesis, lipid-raft remodelling and tumour stemness. Lactate and histone lactylation (H3K18la/H4K5la) reinforce Treg, M2-like tumour-associated-macrophage and PD-L1 programmes while promoting CD8+ T-cell exhaustion. Cholesterol–ER stress–XBP1, ASNS–RIG-I, kynurenine–AhR-STING and CD39–CD73–adenosine pathways further suppress antitumour immunity.

Collectively, these studies define a metabolic basis for immunotherapy resistance in BC. Lactate and kynurenine suppress effector T cells and expand Treg cells through receptor-mediated and epigenetic mechanisms; lipid metabolism alters T-cell membrane composition and signalling; and asparagine and tryptophan metabolism reduce type I interferon output by changing innate immune sensing. These adaptations weaken ICI efficacy but also identify testable biomarkers and therapeutic windows for co-targeting metabolic–immune axes. Additional mechanisms linking metabolic reprogramming to treatment resistance, including mitochondrial adaptation, lipid remodelling and immunometabolic crosstalk, are summarised in Table 2. Together, these programmes coordinately drive resistance to chemotherapy and the immune-checkpoint blockade (Figure 3).

## 5. Therapeutic Strategies Targeting Metabolic Pathways

Metabolism-directed therapy aims to exploit tumour dependencies but is constrained by network compensation, intratumour heterogeneity and metabolic pathways shared with normal and immune cells [11,157,158,159]. The interventions discussed below are classified as BC-specific preclinical approaches, experimental evidence from other cancer types, or clinical-stage agents evaluated outside BC. In the following subsections, each agent is annotated according to whether the supporting evidence is derived from BC-specific models, cross-cancer preclinical models, or clinical trials performed outside BC. Clinical-trial identifiers, reported outcomes and major toxicities are provided when available, whereas compounds without clinical evaluation are described as experimental.

The potential role of metabolism-directed treatment differs across clinical settings. In NMIBC, BCG and intravesical chemotherapy provide the immediate treatment context; repeated access to the bladder may permit local metabolic intervention and urinary pharmacodynamic monitoring, although no metabolism-directed agent has established intravesical efficacy [2]. In localised MIBC, metabolic strategies are most plausibly positioned as sensitizers to cisplatin-based neoadjuvant therapy, given BC-model evidence linking OXPHOS, mitochondrial quality control, the PPP, serine and one-carbon metabolism, and FASN to chemoresistance [6,16,35,101,103]. In advanced or metastatic urothelial carcinoma, enfortumab vedotin plus pembrolizumab, platinum chemotherapy, ICIs and erdafitinib for susceptible *FGFR3*-altered disease define the systemic treatment landscape [160]. The FGFR3–SREBP1–SCD1 axis and the preclinical synergy between erdafitinib and PHGDH inhibition support metabolic co-targeting in *FGFR3*-altered tumours [15,16], whereas lactate-, FASN-, ASNS-, kynurenine- and adenosine-directed approaches may inform combinations with ICIs [97,100,114,126]. Direct evidence that metabolic intervention improves responses to antibody–drug conjugates or intravesical therapy is currently lacking, and these combinations remain investigational. Because enfortumab vedotin depends on Nectin-4-mediated endocytosis and intracellular MMAE release, metabolic changes that alter membrane composition or trafficking may affect ADC processing. In BC models, α2,6-sialylation reduced Nectin-4 abundance and EV endocytosis, whereas desialylation enhanced EV activity; whether lipogenesis itself alters EV uptake or clinical response remains unknown [161]. Throughout this section, findings obtained in non-urothelial tumour types are identified as cross-cancer evidence and are used only to support biological rationale. They do not constitute direct evidence of efficacy in bladder cancer, and any therapeutic conclusions based on such evidence remain investigational until validated in BC-specific models or clinical trials.

### 5.1. Targeting Glycolysis

The dependence of BC cells on aerobic glycolysis makes key glycolytic enzymes rational therapeutic targets. HK2, LDHA and PKM2 are dysregulated in BC and are associated with progression, chemoresistance and poor prognosis [22,23].

#### 5.1.1. Hexokinase-Directed Strategies

BC cell-line and chicken chorioallantoic-membrane studies show that 2-DG suppresses tumour-associated phenotypes and restores cisplatin sensitivity, although its low potency and off-target effects limit clinical development [22,162]. Oridonin targets HK1 rather than HK2; in BC models, it inhibits glycolysis, reduces lactate-associated PD-L1 expression and enhances the effect of PD-L1 blockade [163]. CircST6GALNAC6 suppresses glycolysis by reducing HK1 stability through the PRKN–HK1 axis [76].

#### 5.1.2. LDHA Inhibitors

LDHA catalyses the final glycolytic step, reducing pyruvate to lactate. It also maintains glycolytic continuity through NAD+ regeneration and supports lactate export. Elevated serum lactate dehydrogenase (LDH) is strongly associated with poor prognosis in BC; meta-analysis links high LDH to shorter overall, cancer-specific and disease-free survival in urothelial carcinoma [164,165]. Antitumour effects of LDHA inhibition have been demonstrated in several solid-tumour models. In melanoma, anti-PD-1 treatment of *LDHA*-deficient tumours increases NK-cell and CD8+ T-cell infiltration and production of interferon gamma (IFN-gamma) and granzyme B, reduces Treg infiltration and markedly suppresses tumour growth [166]. In a humanised lung-cancer model, the LDHA inhibitor oxamate enhances anti-PD-1 efficacy [167]. These findings were obtained in melanoma and lung-cancer models and provide cross-cancer rationale rather than direct evidence in BC. Their relevance to BC requires validation in immunocompetent BC models and clinical studies. Cinnamaldehyde induces apoptosis and reduces glucose consumption and lactate production in 5637 BC cells by inhibiting the Erb-B2 receptor tyrosine kinase 2 (ErbB2)–heat shock factor 1 (HSF1)–LDHA pathway [168]. Silibinin downregulates PD-L1 in nasopharyngeal carcinoma cells by disrupting HIF-1α–LDHA-mediated glucose metabolism [169]. Cinnamaldehyde therefore provides BC cell-line evidence, whereas the findings for silibinin remain cross-cancer evidence and do not demonstrate therapeutic activity in BC.

#### 5.1.3. PKM2 Modulators

PKM2 has been investigated as a therapeutic target because its metabolic and nuclear functions support BC growth and treatment resistance [55,71,72,170]. In BC models, mannose alters PKM2 activity and post-translational modification, induces NLRP1–caspase-1–GSDMD-dependent pyroptosis and enhances ICI efficacy, whereas oxymatrine increases cisplatin sensitivity [171,172]. Responses to 3-BrPA differ between T24 and RT4 cells, indicating genotype-dependent activity [173]. Dual PKM2/FASN inhibition with SRT3025-loaded liposomes enhances oxaliplatin activity, but the evidence remains preclinical [174].

### 5.2. Targeting Lipid Metabolism

BC cells increase de novo fatty-acid and cholesterol synthesis to meet the membrane-biosynthesis demands of rapid proliferation. Several key lipid-metabolic enzymes have therefore emerged as candidate therapeutic targets [56,82].

#### 5.2.1. FASN Inhibitors

BC-specific studies associate FASN with poor prognosis, gemcitabine resistance and acquired resistance to PD-1 blockade; TVB-3166 and orlistat reverse these resistance phenotypes in BC cell and animal models [35,126,175]. Denifanstat has not been evaluated clinically in BC. In a phase I study of advanced solid tumours (NCT02223247), denifanstat monotherapy produced stable disease in 42% of evaluable patients but no complete or partial responses. In combination with a taxane, the partial-response rate was 11% and the disease-control rate was 70% [176]. Dose-limiting toxicities mainly involved reversible skin and ocular effects; alopecia, palmar–plantar erythrodysesthesia and fatigue were common, and one treatment-related fatal pneumonitis occurred in the combination group [176]. These cross-cancer findings establish target engagement and safety parameters but do not demonstrate efficacy in BC.

#### 5.2.2. Cholesterol-Metabolism Targets

SOAT1 catalyses cholesterol esterification and is highly expressed in BC, where it correlates positively with tumour grade. The SOAT inhibitor avasimibe activates PPARγ signalling, inhibits BC-cell proliferation and migration, induces G1 arrest and increases ROS [136]. Simvastatin likewise activates PPARγ, induces G0/G1 arrest and suppresses epithelial–mesenchymal transition and AKT–GSK3beta signalling [177]. Mevalonate-pathway inhibition can also reduce BC metastasis by regulating RhoB protein stability and integrin beta1 localisation [33]. Metformin targets clusterin to regulate the SREBP-1c–FASN axis and suppress lipid synthesis and growth in BC cells [178]. In colorectal cancer and melanoma, metformin has shown immunomodulatory and anticancer activity [179,180]; however, its efficacy in patients with BC without diabetes requires prospective clinical evaluation.

#### 5.2.3. Gut Microbiota-Lipid Metabolism Axis

Recent work indicates that gut-microbial metabolites can influence BC progression through lipid metabolism. Indole-3-acetic acid (3-IAA) derived from Parabacteroides distasonis activates AhR, downregulates *FASN* transcription and lowers the monounsaturated-to-polyunsaturated fatty-acid ratio, thereby increasing ferroptosis sensitivity in BC cells. P. distasonis abundance is markedly reduced in patients with BC, and low abundance is associated with poor prognosis [155]. This finding connects the gut microbiota, lipid metabolism and ferroptosis and suggests that microbiome modulation may offer a new route to targeting lipid metabolism in BC.

### 5.3. Targeting Amino-Acid Metabolism

#### 5.3.1. Glutamine Metabolism

Telaglenastat has not been evaluated clinically in BC. In the randomised phase II CANTATA trial in advanced renal-cell carcinoma (NCT03428217), telaglenastat plus cabozantinib did not improve median progression-free survival compared with placebo plus cabozantinib (9.2 versus 9.3 months) [181]. Grade 3–4 adverse-event rates were similar between the groups, with hypertension and diarrhoea among the most frequent severe events. Preclinical studies in non-BC mouse models suggest that glutamine antagonism may affect tumour and effector T cells differently [182], but this therapeutic window requires confirmation in immunocompetent BC models.

#### 5.3.2. Proline Metabolism

PYCR1 is a key proline-synthesis enzyme that reduces P5C to proline while consuming NAD(P)H. In BC, PYCR1 promotes tumour growth through AKT-Wnt–β-catenin signalling, and SENP3-mediated deSUMOylation of STAT3 increases *PYCR1* transcription [94,95]. Hypoxia-induced PYCR1 also increases lactate and H3K18la, upregulates *SLC6A14* and glutamine uptake and establishes a metabolic–epigenetic feedback loop [58]. Its high expression and association with poor prognosis make *PYCR1* a potential metabolic target in BC. However, PYCR1 is also expressed in CAFs and normal connective tissue, and loss-of-function mutations cause cutis laxa; isoform-selective inhibitors with favourable safety profiles are therefore needed [96].

#### 5.3.3. Arginine Deprivation

ASS1 catalyses regeneration of arginine from citrulline. BC cells with epigenetically silenced *ASS1* cannot efficiently use citrulline to synthesise arginine and consequently depend on exogenous arginine. ADI-PEG20 depletes circulating arginine and preferentially suppresses the growth of *ASS1*-low BC [93]. Compensatory glutamine, serine and mitochondrial metabolism induced by arginine deprivation may, however, limit the efficacy of monotherapy [92]. This evidence is derived mainly from cross-cancer studies and requires confirmation in BC models.

### 5.4. Metabolic-Immunotherapy Combination Strategies

Metabolic reprogramming not only drives tumour progression but also profoundly shapes immune-cell function in the TME. Combining metabolism-directed agents with ICIs has therefore become a frontier in precision treatment for BC. Tumour-cell glycolysis consumes large quantities of glucose and produces lactate, creating nutrient deprivation and acidosis that suppress CD8+ T-cell proliferation and effector function [183,184]. LDHA or lactate-transport inhibitors can lower TME lactate and restore CD8+ T-cell mitochondrial function and cytotoxicity [166,185]. FASN inhibitors or cholesterol modulators can alter the lipid composition of the TME and reduce immunosuppressive activity in Treg cells and M2-like macrophages [155,175]. Natural products such as oridonin combine HK1 targeting with PD-L1 inhibition, illustrating the potential for a single compound to engage metabolic and immune targets [163].

In BC models, circFAM13B suppresses glycolysis through the IGF2BP1–PKM2 pathway, reduces TME acidification and increases sensitivity to PD-1 blockade [125]. Oridonin also enhances CD8+ T-cell cytotoxicity against BC cells by inhibiting HK1-dependent glycolysis and lactate-associated PD-L1 expression [163]. By contrast, evidence for the immunometabolic effects of metformin, silibinin and LDHA inhibition in combination with ICIs derives mainly from colorectal cancer, melanoma, lung cancer or nasopharyngeal carcinoma [166,167,169,179,180]. These cross-cancer findings provide a rationale for investigation but do not demonstrate therapeutic efficacy in BC. The cited natural-product studies should therefore be regarded as pharmacological proof of concept; their selectivity, potency, formulation-dependent bioavailability, clinically achievable exposure and safety in combination with standard BC treatment remain unestablished.

Despite its conceptual appeal, metabolic-immunotherapy combination treatment faces several translational barriers. Metabolic agents may impair effector T-cell metabolism and normal tissues that share glucose, glutamine and lipid pathways with tumour cells. Systemic administration may therefore narrow the therapeutic window. The reversible skin and ocular toxicities observed with denifanstat and the high adverse-event burden of combination regimens illustrate the need to optimise dose, schedule and route of administration while confirming pathway inhibition within the tumour [176,181]. Although glutamine antagonism can enhance T-cell function under selected conditions [182], excessive inhibition of glycolysis or glutamine metabolism may compromise T-cell proliferation and effector differentiation. Patient-selection biomarkers remain poorly defined, and pretreatment tissue metabolomics, metabolic imaging or liquid biopsy may be required to identify functional tumour dependencies. Treatment sequence and local delivery also require systematic evaluation [186,187].

A critical challenge for combining glycolysis inhibitors with ICIs is the metabolic competition between tumour cells and activated effector T cells. Cytotoxic lymphocytes rely on aerobic glycolysis for proliferation and production of effectors such as IFN-γ, raising the concern that systemic glycolysis inhibition may impair antitumour immunity. However, several lines of evidence suggest that a therapeutic window can be achieved. Tumour cells exhibit higher basal glycolytic rates and LDH expression than tumour-infiltrating T cells, and preclinical studies show that tumour-specific glycolysis inhibition can relieve metabolic suppression while preserving T-cell function. In bladder cancer models, combining LDHA inhibitors with anti-PD-1 therapy significantly reduced tumour volume compared with anti-PD-1 alone, and in melanoma models, LDHA inhibition increased CD8+ T-cell infiltration and restored IFN-γ secretion. Intermittent rather than continuous dosing may further preserve immune-cell fitness: low-dose, short-term exposure to 2-DG can increase IFN-γ secretion in activated T cells, whereas prolonged exposure suppresses T-cell reactivity. Tumour-targeted delivery systems, including nanoparticles and antibody–drug conjugates, may also limit systemic exposure. The therapeutic window and optimal dosing schedule for glycolysis inhibitors in combination with ICIs therefore require prospective evaluation in early-phase clinical trials rather than being inferred from preclinical efficacy alone [166,185].

Intravesical delivery may limit systemic exposure in NMIBC, but it introduces formulation-specific constraints. Continuous urine production dilutes instilled agents, and voiding shortens mucosal contact time. Urinary pH and solute composition can alter drug stability and carrier-release kinetics; acidic urine destabilises some agents, including apaziquone. The glycosaminoglycan/mucus layer, urothelial plaques and tight junctions further restrict tissue penetration. Candidate metabolism-directed agents will therefore require urine-stable formulations that prolong bladder residence and improve mucosal transport, such as mucoadhesive carriers, hydrogels or sustained-release devices. These platforms remain investigational and do not yet establish clinical efficacy for intravesical metabolic therapy in BC [188,189].

Metabolism-directed therapies in BC remain predominantly preclinical. Their development will require biomarker-defined dependencies, evidence of target inhibition and combinations that preserve antitumour immune function. Additional targets are summarised in Table 3.

## 6. Metabolic Biomarkers

Metabolomics measures small-molecule metabolites in biological samples and can provide a biochemical readout of the combined effects of genetic background, epigenetic regulation, the microenvironment and treatment [194,195,196,197]. However, steady-state metabolite abundance does not by itself establish pathway flux or clinical utility. Urine concentrates many metabolites and permits repeated non-invasive sampling, whereas tissue metabolomics can capture biochemical differences among tumour regions [198,199]. Advances in high-resolution mass spectrometry (HRMS), nuclear magnetic resonance (NMR) and standardised bioinformatic workflows have expanded the use of metabolomics for early detection, molecular classification, recurrence surveillance and response prediction in BC [200,201,202]. Considerable barriers remain between biomarker discovery and clinical application, notably, confidence in metabolite identification, standardisation of specimen collection and insufficient validation in independent cohorts [203].

### 6.1. Metabolomics for Bladder Cancer Diagnosis

Metabolomics is being investigated as a source of candidate diagnostic markers in BC, but no metabolite panel described here is ready for routine clinical use. Urine captures metabolites, cellular debris and secreted small molecules released by bladder tumour cells, and its completely non-invasive, repeatable collection makes it an attractive liquid-biopsy matrix [200]. Untargeted metabolomic analysis of 200 human urine samples by ultra-high-performance liquid chromatography–ultra-high-resolution mass spectrometry (UHPLC–UHRMS), integrating univariable and multivariable statistics with external validation, identified several metabolites that distinguished BC from healthy controls [200]. Urine from 100 patients with BC and 100 controls was also analysed by NMR and two high-resolution nanoparticle-assisted laser desorption/ionisation mass-spectrometry (LDI-MS) platforms. Five NMR-detected metabolites and 25 LDI-MS-detected compounds, principally peptides and lipids, were identified as potential BC indicators. Receiver operating characteristic (ROC) analysis showed strong predictive performance for all three metabolomic datasets, with areas under the curve (AUCs) exceeding 0.87 [201]. A separate study used C18 silica and hydrophilic interaction liquid chromatography (HILIC) columns with UHPLC–UHRMS to profile polar and non-polar metabolites in tissue from 99 patients with BC and identified 25 candidate discriminatory metabolites [199].

Several studies have identified specific metabolite panels. A discovery cohort of 332 samples, including 66 BC-positive samples, yielded 25 candidate biomarkers that were tested in an independent cohort. Palmitoyl sphingomyelin, lactate, adenosine and succinate showed the strongest ability to distinguish BC from non-cancer controls, with lipid-related metabolites being particularly informative [198]. Plasma metabolomics also has diagnostic potential. A pseudo-targeted gas chromatography–mass spectrometry (GC–MS) approach found that metabolites related to the PPP, nucleotide synthesis and fatty-acid synthesis were significantly increased in plasma from patients with high-grade BC relative to healthy controls, consistent with PPP activation supporting lipid and nucleotide biosynthesis and ROS detoxification during BC-cell proliferation [204]. Liquid chromatography–tandem mass spectrometry (LC–MS/MS) profiling showed that a four-amino-acid panel comprising ornithine, methionine, arginine and glutamate distinguished incident BC cases from controls in that cohort with an AUC of 0.84 [205]. Metabolomics may also distinguish BC from renal cell carcinoma (RCC). In an untargeted ultra-high-performance liquid chromatography-mass spectrometry (UPLC-MS) urine study of 403 participants, separate panels distinguished BC from RCC in patients without haematuria (AUC = 0.829) and with haematuria (AUC = 0.913) [206].

Tissue metabolomics further reveals stage-related differences in BC. Integrated GC–MS and LC–MS platforms identified significant differences between NMIBC and MIBC in glucose, TCA-cycle, lipid, amino-acid and nucleotide pathways. Increased TCA-cycle intermediates, amino acids and dipeptides in advanced disease were consistent with increased anaplerosis to sustain energy production. Compared with NMIBC, MIBC showed stronger cyclooxygenase and lipoxygenase signalling, increased haem catabolism and altered NAD+ synthesis [60]. Lipidomic analysis further showed progressive decreases in phosphatidylserine, phosphatidylethanolamine and phosphocholine and an increase in diacylglycerol with advancing stage. Diacylglycerol and lysophosphatidylethanolamine were particularly elevated in tumours with lymphovascular invasion and nodal involvement, respectively. Transcriptomic and protein-expression analyses showed that phosphatidylserine synthase 1, which converts diacylglycerol to phosphatidylserine, progressively decreased with advancing stage [207].

Reported diagnostic AUCs should be interpreted in relation to cohort design and validation. Wittmann et al. tested candidate metabolites in an independent urine cohort that included haematuria and surveillance controls [198], and Nizioł et al. included patients with benign urological conditions such as urinary stones [199]. Other studies relied primarily on comparisons with healthy controls [201,204,205]. Few panels have been evaluated concurrently against urinary tract infection, inflammation, renal dysfunction and other genitourinary malignancies, although one study directly compared BC with renal-cell carcinoma in patients stratified by haematuria [206]. Head-to-head comparisons with urine cytology remain uncommon, and cystoscopy generally served as the diagnostic reference rather than a comparator. These panels should therefore be considered candidate adjuncts to cytology and cystoscopy until blinded prospective multicentre validation demonstrates calibration, clinical utility and added diagnostic value.

Cross-centre reproducibility remains a major barrier to urinary metabolomics. Collection timing, hydration and fasting status, processing delay, storage temperature, freeze–thaw cycles and the method used to correct urine dilution can alter measured concentrations independently of tumour biology. Multicentre studies therefore require prespecified protocols for sample collection, processing and storage, together with common quality control materials, instrument calibration and data normalisation procedures. Until these procedures and prospective external validation are in place, reported AUCs should be regarded as cohort-specific discovery performance rather than evidence of clinical readiness [208].

### 6.2. Metabolic Biomarkers of Treatment Response and Prognosis

Metabolic biomarkers also show promise for predicting prognosis and treatment response. A systematic review and meta-analysis of 15 studies involving 5,491 patients with NMIBC evaluated associations between pretreatment metabolites and clinical outcomes. Pooled hazard ratios linked metabolite measures to progression-free survival (HR = 4.48, 95% confidence interval (CI) 1.70–11.80), recurrence-free survival (HR = 2.85, 95% CI 1.91–4.26) and overall survival (HR = 1.78, 95% CI 1.07–2.98), supporting their potential as independent prognostic factors [209]. In postoperative surveillance, longitudinal urinary metabolomics identified significant differences in hippurate, glutarate and uridine between preoperative samples and those collected two weeks after surgery, suggesting potential to distinguish disease from health. Targeted quantitative metabolomics using linear mixed-effects models further showed that 2-methylthioadenosine decreased 24 h after surgery, whereas inosine increased, providing dynamic markers for postoperative monitoring [210,211]. Quantitative analysis of urinary nucleosides and deoxynucleosides across seven time points, before surgery and at 24 h, 2 weeks, and 3, 6, 9 and 12 months after surgery, characterised longitudinal excretion patterns relevant to recurrence surveillance. Lower natural carbon-13 abundance in histologically normal urothelium was significantly associated with shorter disease-free survival, suggesting that isotope analysis of normal tissue may help predict recurrence [212].

Expression of specific metabolic enzymes is also closely associated with prognosis. High HK2, an independent prognostic factor in BC, correlates with shorter disease-free and overall survival [22]. Elevated serum LDH is associated with poorer overall (HR = 1.61), cancer-specific (HR = 1.41) and disease-free (HR = 1.64) survival in urothelial carcinoma, and an evidence-based analysis of 2,182 patients confirmed its association with overall survival in BC (HR = 1.86) [164,165]. Serum FASN and LDH are increased in BC, with higher levels in stage III–IV than in stage I–II disease, supporting their potential utility in diagnosis and clinical management [213].

Metabolism-related gene signatures should currently be regarded as retrospective, hypothesis-generating models. The fatty-acid, glutamine and glycolysis-related signatures described here were derived mainly from public transcriptomic datasets [126,214,215,216]. Some were evaluated in additional datasets, including IMvigor210, but none has undergone prospective validation as a prespecified clinical classifier or demonstrated incremental value beyond established factors such as stage, grade, nodal status and performance status. An association with outcome in an ICI-treated cohort does not establish predictive value without an appropriate treatment-comparator cohort. Drug sensitivity estimates derived from transcriptomic algorithms and compounds nominated by molecular docking are computational hypotheses and should not be interpreted as evidence of therapeutic response without pharmacological and clinical validation.

Genotype may provide an initial layer of patient stratification for metabolic–immunotherapy combinations, although neither *KDM6A* loss nor *FGFR3* alteration is clinically validated for this purpose. *KDM6A* loss reduced tumour lactate and H3K9/H3K18 lactylation in Treg cells and improved anti-PD-1 activity in preclinical BC models, despite increasing cisplatin resistance through impaired DNA repair [31]. *FGFR3*-altered tumours can depend on PI3K–mTORC1–SREBP1–SCD1-mediated lipid desaturation, but these tumours remain heterogeneous in lipid homeostasis and apoptotic sensitivity [12,13,14,15]. *KDM6A* and *FGFR3* status should therefore be interpreted together with functional measures such as lactate output, SCD1 activity, tumour lipid composition and spatial immune-cell profiles. Such composite biomarkers may support biomarker-defined evaluation of metabolic agents combined with an immune-checkpoint blockade.

Among imaging biomarkers, fluorine-18 fluorodeoxyglucose positron-emission tomography/magnetic-resonance imaging (18F-FDG PET/MRI) performs well in BC staging and assessment of response to neoadjuvant therapy, and change in mean standardised uptake value (DeltaSUVmean) is a candidate response parameter [217]. The method of 18F-FDG positron-emission tomography/computed tomography (PET/CT) is also clinically useful for staging high-risk NMIBC before radical cystectomy. Among 92 patients, PET/CT detected metastases in 15%, changed stage in 12% and altered treatment in 10%; sensitivity (36% versus 12%), specificity (93% versus 97%) and accuracy (77% versus 74%) were compared with contrast-enhanced CT [218] 4. The role of PET in BC management is expanding, and novel tracers, including gallium-68- or fluorine-18-labelled fibroblast-activation-protein inhibitors (FAPIs), are entering clinical investigation [219]. Deuterium metabolic imaging may complement conventional imaging by detecting treatment-related changes in glucose-to-lactate conversion before anatomical response, although evidence in BC remains limited to cell and murine models [80]. Translation to pelvic imaging is constrained by low ^2^H sensitivity and limited spatial resolution at clinical field strengths and will require optimised coils, standardised tracer dosing and acquisition, reproducible quantification, and control of motion and field inhomogeneity [220,221]. Progressive bladder filling and any urine-derived ^2^H signal may also cause partial-volume contamination; this bladder-specific effect has not yet been quantified in patients.

Collectively, metabolomics provides multidimensional sources of biomarkers for diagnosis, prognostication and treatment-response monitoring in BC. Additional metabolomic biomarker studies, including urinary volatilomics, tissue metabolite profiling, and exosomal markers, are summarised in Table 4. Urinary metabolite panels, tissue lipid profiles, serum enzymes and metabolic imaging parameters each have distinct advantages and limitations. Systemic inflammatory indices may complement these metabolic biomarkers. In a retrospective cohort of 285 patients with NMIBC, elevated platelet-to-lymphocyte ratio and systemic inflammation response index were associated with recurrence, while higher systemic inflammation response index, pan-immune-inflammation value and platelet-to-lymphocyte ratio were associated with lymphovascular invasion or nodal metastasis [222]. These findings support further evaluation of combined metabolic and immune-inflammatory biomarker models, although external prospective validation is required. Future work should prioritise integration of multimodal biomarker panels and prospective clinical validation. Maturing spatial and single-cell metabolomics technologies should further resolve intratumour metabolic heterogeneity and enable more precise stratification and individualised treatment.

## 7. Conclusions

BC-specific studies identify several context-dependent metabolic vulnerabilities. These include SCD1-dependent lipid desaturation in *FGFR3*-altered tumours, arginine dependence associated with *ASS1* deficiency, amino-acid pathways linking ASNS–RIG-I and AhR–STING signalling to immune responses, and OXPHOS, serine and one-carbon metabolism, and FASN in chemotherapy resistance [6,15,16,93,97,100]. Most therapeutic evidence remains preclinical. Moreover, results from trials conducted in other cancer types show that target expression or in vitro sensitivity does not ensure pathway inhibition or clinical benefit [156,176,181]. Candidate targets should therefore be prioritised according to BC-specific functional evidence, treatment setting and an achievable therapeutic window.

Clinical translation will require patient selection based on combined molecular and functional biomarkers. *FGFR3* or *KDM6A* status may define relevant biological contexts but should be integrated with isotope tracing, tissue or urinary metabolomics, spatial immune profiling and on-treatment pharmacodynamic measurements [31,231,232,233]. Patient-derived organoids and immunocompetent orthotopic models can be used to assess metabolic dependency and immune effects before clinical evaluation [234,235]. Early trials should incorporate paired tissue, urine or imaging measurements, prespecified evidence of target inhibition, and dose modification rules that account for toxicity in normal and immune cells. Intravesical delivery may be suitable in NMIBC when local exposure and washout barriers are addressed [236], whereas systemic combinations in MIBC or metastatic disease require evidence that antitumour immunity is preserved. The central priority is to match a measurable metabolic dependency to the appropriate patient, disease stage and treatment.

## Figures and Tables

**Table 1:** Non-coding RNAs implicated in bladder cancer metabolic reprogramming.

Non-Coding RNA	Metabolic Target	Primary Molecular Mechanism	Functional Biological Outcome	References
*SLC16A1-AS1*	SLC16A1/MCT1; glycolysis, OXPHOS and fatty-acid oxidation	E2F1-responsive lncRNA and E2F1-binding partner; increases transporter and PPARα-related gene expression	Multi-substrate use; tumour growth and invasion	[48]
*UCA1*	GPT2; glutamine anaplerosis	Binds hnRNP I/L and increases GPT2 expression	Greater glutamate-to-α-KG conversion and TCA replenishment	[90]
*circST6GALNAC6*	HK1; glycolysis	Promotes PRKN-dependent HK1 destabilisation	Lower glycolysis and tumour growth	[76]
*circFOXK2*	TACO1–MTCO1; mitochondrial OXPHOS	Scaffolds HSP90β–TACO1 interaction and TACO1 mitochondrial translocation	Complex IV activity, stemness and cisplatin tolerance	[101]
*circFAM13B*	IGF2BP1–PKM2; glycolysis	Suppresses PKM2-linked glycolytic programme	Less acidification; improved anti-PD-1 response in BC models	[125]
*AC107027.3/miR-27A-3p*	FASN; fatty-acid synthesis	Proposed competing endogenous RNA interaction; increased FASN expression	Putative association with immunotherapy response; functional validation needed	[126]
*circZNF609*	IGF2BP2–CD36; fatty-acid uptake	Increases CD36-linked lipid uptake	Lower TME fatty-acid availability; reduced immunotherapy sensitivity	[127]
*miR-146a-5p*	ARID1A/PRKAA2; STAT3–mTOR signalling	CAF-derived exosomal miRNA targets ARID1A and PRKAA2	Stemness and gemcitabine/cisplatin resistance	[128]

**Table 2:** Impact of metabolic reprogramming on therapeutic resistance in bladder cancer. Mechanisms are condensed from the evidence discussed in Section 4.

Treatment	Key Metabolic Alteration	Mechanism	Impact on Therapy	Evidence Source and Disease Context	Refs
Cisplatin	Rapid OXPHOS upregulation	Early complex I–OXPHOS surge; increased ATP buffering.	Provides early metabolic buffer against cisplatin cytotoxicity; complex I inhibitor phenformin abolishes this protective effect	BC cell lines	[6]
Cisplatin	PDK4-mediated metabolic checkpoint	PDK4–PDH control of pyruvate oxidation; acute inhibition differs from chronic knockout.	Acute PDK4 inhibition with DCA or siRNA sensitises bladder cancer cells to cisplatin and suppresses xenograft growth. In contrast, chronic PDK4 deficiency in the BBN model increases tumour volume and stage, indicating that acute inhibition and chronic loss are not equivalent. This differential effect supports intermittent rather than continuous PDK4 inhibition.	BC cell lines and xenografts; BBN-induced bladder cancer mouse model	[74]
Cisplatin/Gemcitabine	PGC-1α-driven mitochondrial biogenesis	PGC-1α–NRF1–TFAM mitochondrial biogenesis and OXPHOS.	Nanoparticle-delivered si-PGC-1α targeting mitochondrial metabolism inhibits metastasis, revealing PGC-1α dependency as a metabolic vulnerability	BC cell lines + mouse metastasis model	[153]
Gemcitabine/Cisplatin	IDH2-mediated reductive glutamine metabolism	IDH2-dependent glutamine reductive carboxylation; citrate and NADPH/GSH supply.	Confers gemcitabine resistance and cross-resistance to cisplatin; IDH2 represents a potential target for restoring chemosensitivity	Drug-resistant BC cell lines	[16]
Gemcitabine/Cisplatin	FASN-mediated de novo lipogenesis	FASN-driven de novo palmitate synthesis.	TVB-3166 reverses drug resistance; patients with low *FASN* expression show better response to ICI therapy	Drug-resistant cell lines + xenografts + patient cohorts	[35]
Anti-PD-1/PD-L1	ASNS/asparagine metabolism	ASNS-derived asparagine drives CBL-mediated RIG-I degradation; type I IFN suppression.	High *ASNS* expression correlates with poor ICI response; L-asparaginase depleting asparagine restores type I IFN signalling and synergizes with anti-PD-1 to suppress tumour growth	BC cell lines + immune-competent mice + patient cohorts	[97]
Anti-PD-1/PD-L1	IDO1/TDO–kynurenine–AhR axis	Tryptophan depletion–GCN2 stress; kynurenine–AhR signalling and STING loss.	Chemotherapy-induced type I IFN is attenuated, reducing immunogenicity; AhR blockade or STING restoration enhances chemotherapy-related immune activation	Bladder cancer patient plasma + BC cell lines	[100]
Anti-PD-1/PD-L1	CD73/adenosine pathway	CD73-generated adenosine activates A2A/A2B–cAMP immunosuppression.	Adenosine impairs CD8^+^ T and NK cell effector function and favours Treg maintenance; CD73 inhibitor oleclumab plus durvalumab has entered clinical trials (NCT03773666)	Bladder cancer patient tissues + clinical trial	[114]
Anti-PD-1/PD-L1	CAF/exosomal miR-146a-5p/STAT3/mTOR	CAF exosomal miR-146a-5p targets ARID1A/PRKAA2; STAT3–mTOR activation.	Promotes cancer stemness and gemcitabine/cisplatin resistance; serum exosomal miR-146a-5p levels correlate with bladder cancer recurrence, serving as a liquid biopsy biomarker	BC cell lines + mouse models + patient serum	[128]
Anti-PD-1/PD-L1	*SLC14A1*^+^ CAF/WNT5A	IFN-induced SLC14A1^+^ CAF–WNT5A signalling activates tumour β-catenin.	*SLC14A1*^+^ CAF infiltration correlates with poor response to NAC and immunotherapy; targeting STAT1 or STING sensitises to chemotherapy	Bladder cancer single-cell sequencing + patient cohorts	[154]
Anti-PD-1/PD-L1	25-hydroxycholesterol/EMT	CH25H-derived 25-hydroxycholesterol alters LXR and lipid-raft signalling.	Elevated 25-HC in bladder cancer tissues correlates with poor prognosis; suggests statins or CH25H inhibitors may have therapeutic value	BC cell lines + patient tissues	[150]
Anti-PD-1/PD-L1	p53/macrophage lipid reprogramming	Aberrant p53 associates with lipid-catabolic SPP1^+^MARCO^+^ macrophages.	This TAM subset correlates with poor outcomes to platinum-based therapy and anti-PD-L1 therapy; targeting p53 mutation or TAM metabolism may improve therapeutic response	Bladder cancer patient tissues + immune-deficient mice	[49]
Anti-PD-1/PD-L1	3-IAA/AhR/FASN/ferroptosis	Microbial 3-IAA–AhR signalling suppresses SREBP1–FASN; ferroptosis sensitisation.	Gut microbiota–metabolism–immune crosstalk represents a therapeutic target; low P. distasonis abundance correlates with poor prognosis in bladder cancer	BC cell lines + organoids + orthotopic mouse models + patient cohorts	[155]
Anti-PD-1/PD-L1	IDO1	IDO1 blockade reduces tryptophan catabolism; clinical benefit remains unproven.	Phase III ECHO-301 trial failed to demonstrate survival benefit—target expression does not equal pathway flux inhibition, highlighting the need for pharmacodynamic biomarker validation in combination therapy	Melanoma (cross-cancer cautionary example)	[156]

Abbreviations: 3-IAA, indole-3-acetic acid; AhR, aryl hydrocarbon receptor; α-KG, α-ketoglutarate; BBN, N-butyl-N-(4-hydroxybutyl)nitrosamine; BC, bladder cancer; CAF, cancer-associated fibroblast; EMT, epithelial–mesenchymal transition; FASN, fatty acid synthase; GSH, glutathione; ICI, immune-checkpoint inhibitor; IDH2, isocitrate dehydrogenase 2; IDO1, indoleamine 2,3-dioxygenase 1; IFN, interferon; MUFA, monounsaturated fatty acid; NAC, neoadjuvant chemotherapy; NF-κB, nuclear factor kappa B; OXPHOS, oxidative phosphorylation; PD-1, programmed cell death protein 1; PD-L1, programmed death-ligand 1; PDK4, pyruvate dehydrogenase kinase 4; PGC-1α, peroxisome proliferator-activated receptor gamma coactivator 1-alpha; PUFA, polyunsaturated fatty acid; SLC14A1, solute carrier family 14 member 1; SREBP1, sterol regulatory element-binding protein 1; TAM, tumour-associated macrophage; TCA, tricarboxylic acid; TDO, tryptophan 2,3-dioxygenase; Treg, regulatory T cell.

**Table 3:** Targeted metabolic interventions in bladder cancer. The table summarises metabolic targets and intervention strategies that are not fully elaborated in Section 5.

Target	Intervention	Mechanism	Stage of Development	Key Finding	Evidence Source and Disease Context	Refs
SCD1	SCD1 inhibition (RNAi/pharmacological)	SCD1 converts saturated fatty acids to monounsaturated fatty acids; its inhibition disrupts membrane lipid homeostasis and induces ER stress	Preclinical	*SCD1* knockdown suppresses FGFR3-driven bladder tumour growth; SCD1 is downstream of FGFR3–PI3K–mTORC1–SREBP1 signalling	BC cell lines + xenografts	[15]
SQLE	SQLE inhibitor (FR194738)	SQLE catalyses the first oxygenation step in cholesterol biosynthesis; its inhibition reduces cholesterol production and suppresses invasive programmes	Preclinical (prostate cancer)	*PTEN*/p53 deficiency transcriptionally upregulates *SQLE* via SREBP2; *PTEN* loss enhances SQLE protein stability via PI3K/Akt/GSK3β; pharmacological SQLE blockade suppresses invasion	Prostate cancer (mechanism relevant to bladder cancer)	[190]
ASS1	ADI-PEG20 (arginine deiminase)	ASS1 is the key enzyme for regenerating arginine from citrulline; *ASS1*-epigenetically silenced bladder cancer cells cannot effectively utilise citrulline for arginine synthesis and are thus dependent on exogenous arginine	BC preclinical; exploratory patient PET imaging	ADI-PEG20 degrades circulating arginine, preferentially inhibiting the growth of *ASS1*-low bladder cancer; PET imaging enables longitudinal monitoring of ASS1 activity as a pharmacodynamic biomarker	BC cell lines and xenografts; exploratory PET imaging in patients, not a therapeutic trial	[93]
GFAT1/HBP	GFAT1 inhibition/sEV-targeted therapy	GFAT1 is the rate-limiting enzyme of the hexosamine biosynthesis pathway (HBP); HBP generates UDP-GlcNAc for O-GlcNAcylation, which regulates protein function and gene expression	Preclinical	Bladder cancer-derived small extracellular vesicles deliver GFAT1 to endothelial cells, reprogramming glucose metabolism toward HBP flux and enhancing SerRS O-GlcNAcylation, which promotes angiogenesis	BC cell lines + endothelial cells + mouse models	[191]
FASN	Orlistat	FASN catalyses the condensation of acetyl-CoA and malonyl-CoA to generate palmitate; FASN inhibition reduces lipid synthesis	Preclinical (combination with immunotherapy)	In bladder cancer syngeneic tumour models, orlistat reverses acquired resistance to anti-PD-1 therapy, suggesting synergistic potential of FASN inhibition combined with ICI	Bladder cancer syngeneic tumour models	[175]
DHCR7	DHCR7 inhibitor	DHCR7 is a key enzyme in cholesterol synthesis, catalysing the conversion of 7-dehydrocholesterol to cholesterol; its inhibition reduces cholesterol synthesis and lipid raft remodelling	Preclinical	DHCR7 is upregulated in bladder cancer tissues and promotes metastasis via the cAMP/PKA/FAK pathway; DHCR7 inhibition suppresses bladder cancer invasion and metastasis	BC cell lines + xenografts	[137]
OXPHOS	IACS-010759 (complex I inhibitor)	Inhibits mitochondrial complex I, blocking the electron transport chain and OXPHOS, reducing ATP production	Phase I, non-BC solid tumours (NCT03291938; completed)	Phase I testing showed a narrow therapeutic index, with elevated blood lactate and neurotoxicity limiting sustained target exposure. No recommended phase II dose was established, antitumour activity was limited, and the trials were discontinued	Advanced solid tumours and acute myeloid leukaemia; no BC-specific clinical evaluation	[192]
FASN	TVB-2640 (Denifanstat)	FASN catalyses de novo fatty acid synthesis; FASN inhibition reduces lipid synthesis and induces ferroptosis	Phase I, non-BC solid tumours (NCT02223247)	Monotherapy produced stable disease in 42% of evaluable patients without objective responses; combination with a taxane produced an 11% partial-response rate and 70% disease-control rate. Reversible skin and ocular toxicities were dose limiting; alopecia, palmar–plantar erythrodysesthesia and fatigue were common, and one fatal pneumonitis occurred in the combination cohort	Phase I non-BC solid tumours; BC evidence limited to preclinical resistance models	[176]
IDO1	Selective IDO1 inhibitor (Navoximod) + Atezolizumab	Inhibits IDO1-mediated conversion of tryptophan to kynurenine, blocking tryptophan depletion and kynurenine–AhR-mediated immunosuppression	Phase I, advanced solid tumours (NCT02471846)	Navoximod plus atezolizumab showed acceptable safety, but no clear evidence that adding navoximod improved efficacy. Common treatment-related adverse events included fatigue, rash and chromaturia	Advanced solid tumours, including a small urothelial-carcinoma subgroup; not a BC-specific trial	[193]
ACSL5	ACSL5 modulation/activation	ACSL5 activates long-chain fatty acids to acyl-CoAs, increasing acetyl-CoA production; acetyl-CoA promotes 53BP1 K1360 acetylation, recruiting p53–p21	Preclinical	*ACSL5* expression is regulated by DNMT1-mediated methylation; restoring *ACSL5* induces cellular senescence and tumour suppression in bladder cancer; fatty acid-derived acetyl-CoA enters protein acetylation to exert antitumour effects	BC cell lines + xenografts	[86]

Abbreviations: 53BP1, p53-binding protein 1; ACSL5, acyl-CoA synthetase long-chain family member 5; ADI-PEG20, pegylated arginine deiminase; ASS1, argininosuccinate synthetase 1; cAMP, cyclic adenosine monophosphate; DHCR7, 7-dehydrocholesterol reductase; DNMT1, DNA methyltransferase 1; ER, endoplasmic reticulum; FAK, focal adhesion kinase; FASN, fatty acid synthase; GFAT1, glutamine–fructose-6-phosphate aminotransferase 1; GSK3β, glycogen synthase kinase 3β; HBP, hexosamine biosynthesis pathway; ICI, immune-checkpoint inhibitor; IDO1, indoleamine 2,3-dioxygenase 1; mTORC1, mechanistic target of rapamycin complex 1; O-GlcNAc, O-linked β-N-acetylglucosamine; OXPHOS, oxidative phosphorylation; PET, positron emission tomography; PI3K, phosphoinositide 3-kinase; PKA, protein kinase A; PTEN, phosphatase and tensin homologue; SCD1, stearoyl-CoA desaturase 1; SerRS, seryl-tRNA synthetase; sEV, small extracellular vesicle; SQLE, squalene epoxidase; SREBP, sterol regulatory element-binding protein; UDP-GlcNAc, uridine diphosphate N-acetylglucosamine.

**Table 4:** Metabolic biomarkers in bladder cancer. The table summarises biomarker studies not fully elaborated in Section 6.

Biomarker/Technology	Sample Type	Clinical Application	Key Finding	Method	Refs
Gas sensor array	Urine	Diagnosis	Gas sensor array combined with pattern recognition distinguishes bladder cancer patients from healthy controls	Gas sensor array + chemometric pattern recognition	[223]
Urinary volatilomics	Urine	Diagnosis	Global urinary volatilomic profiling identifies bladder cancer-associated VOC signatures	Comprehensive two-dimensional gas chromatography–time-of-flight mass spectrometry (GC×GC-TOF-MS)	[224]
Urinary VOCs + sensors	Urine	Diagnosis	Scoping review of metabolomics and sensor-based approaches for non-invasive bladder cancer diagnosis	Metabolomics + sensor technologies (review)	[225]
Urinary VOCs	Urine	Diagnosis/surveillance	Urinary VOCs serve as novel diagnostic and surveillance biomarkers for bladder cancer	Gas chromatography–mass spectrometry (GC–MS)	[226]
Urinary metabolomics	Urine	Diagnosis/staging	Volatile metabolic biomarkers associated with bladder cancer staging were identified	Urinary metabolomics	[227]
Urinary metabolomics	Urine	Staging/grading	LC–MS-based urinary metabolomics reveals distinct metabolic profiles between NMIBC and MIBC	Liquid chromatography–mass spectrometry (LC–MS)	[228]
Tissue metabolite profiling	Tissue	Diagnosis/grading	C18 + HILIC dual-column system with ultra-high-resolution mass spectrometry identifies 25 diagnostic tissue metabolites, AUC > 0.966	Ultra-high-performance liquid chromatography–ultra-high-resolution mass spectrometry (UHPLC–UHRMS)	[199]
Urinary metabolite panel	Urine	Diagnosis/grading	Five NMR metabolites + 25 LDI-MS compounds distinguish bladder cancer from controls, AUC > 0.87; some correlate with grade and stage	Nuclear magnetic resonance (NMR) + laser desorption/ionisation mass spectrometry (LDI-MS)	[201]
Urinary metabolomics	Urine	Diagnosis	Untargeted urinary metabolomics with ultra-high-resolution mass spectrometry identifies differential metabolites between bladder cancer and controls	Ultra-high-performance liquid chromatography–ultra-high-resolution mass spectrometry (UHPLC–UHRMS)	[200]
Plasma metabolomics	Plasma	Diagnosis	Metabolites involved in the pentose phosphate pathway, nucleotide and fatty acid synthesis are elevated in high-grade bladder cancer patients	Pseudo-targeted gas chromatography–mass spectrometry (GC–MS) metabolomics	[204]
Exosomal metabolites	Urine/serum	Diagnosis	Urinary and serum exosomes serve as novel biomarkers for bladder cancer detection	Exosome isolation + metabolite detection	[229]
Urinary exosomal proteins	Urine	Diagnosis/staging	Protein profile of urinary extracellular vesicles is a marker of malignancy and correlates with muscle invasiveness in bladder cancer	Urinary extracellular vesicle (EV) proteomic profiling	[230]
Lipidomics	Tissue	Staging/prognosis	Bladder cancer tissues show elevated phospholipids and fatty acids and reduced triglycerides; 59 lipid-associated genes correlate with patient survival	Lipidomics (LC–MS/MS)	[207]
Urinary time-series metabolomics	Urine	Post-surgical monitoring/recurrence prediction	Longitudinal urinary profiling at pre-surgery, day 1 post-surgery, and 2 weeks post-surgery reveals significant differences in hippuric acid, glutaric acid, and uridine	High-performance liquid chromatography–time-of-flight mass spectrometry (HPLC-TOF/MS) + gas chromatography–triple quadrupole mass spectrometry (GC-QqQ/MS)	[210]
^13^C/^15^N natural isotope abundance	Tissue (normal urothelium)	Recurrence prediction	^13^C and ^15^N natural isotope abundances are decreased in normal urothelium of bladder cancer patients; lower ^13^C abundance correlates with shorter disease-free survival	Isotope ratio mass spectrometry (IRMS)	[212]
Plasma amino-acid panel	Plasma	Diagnosis	A four-amino-acid panel (ornithine, methionine, arginine, glutamate) diagnoses newly diagnosed bladder cancer, AUC = 0.84	Liquid chromatography–tandem mass spectrometry (LC–MS/MS)	[205]

Reported performance estimates are study-specific and are not directly comparable across platforms or cohorts. External validation, clinically relevant control groups and direct comparison with urine cytology were not available for every study. Abbreviations: AUC, area under the receiver operating characteristic curve; EV, extracellular vesicle; GC–MS, gas chromatography–mass spectrometry; GC–QqQ/MS, gas chromatography–triple quadrupole mass spectrometry; GC×GC–TOF–MS, comprehensive two-dimensional gas chromatography–time-of-flight mass spectrometry; HILIC, hydrophilic interaction liquid chromatography; HPLC–TOF/MS, high-performance liquid chromatography–time-of-flight mass spectrometry; IRMS, isotope ratio mass spectrometry; LC–MS, liquid chromatography–mass spectrometry; LC–MS/MS, liquid chromatography–tandem mass spectrometry; LDI-MS, laser desorption/ionisation mass spectrometry; MIBC, muscle-invasive bladder cancer; NMIBC, non-muscle-invasive bladder cancer; NMR, nuclear magnetic resonance; UHPLC–UHRMS, ultra-high-performance liquid chromatography–ultra-high-resolution mass spectrometry; VOCs, volatile organic compounds.

## Data Availability

Data sharing not applicable to this article as no datasets were generated or analysed during the current study.

## Supplementary Materials

The following supporting information can be downloaded at: https://www.mdpi.com/article/10.3390/cancers18193202/s1, Figure S1: Schematic summary of metabolism-related therapeutic vulnerabilities in bladder cancer. The figure summarizes four major metabolic programs discussed in the re-vised manuscript: glucose metabolism, lipid and cholesterol metabolism, amino-acid metabolism, and mitochondrial metabolism. Each panel highlights representative molecular drivers, altered metabolic pathways, functional outputs, and potential therapeutic targets relevant to bladder cancer progression, treatment resistance, and immune modulation. Abbreviations: OXPHOS, oxidative phosphorylation; PPP, pentose phosphate pathway; TCA, tricarboxylic acid cycle; ROS, reactive oxygen species.

## Abbreviations

2-DG	2-deoxy-D-glucose
3-BrPA	3-bromopyruvate
3-IAA	indole-3-acetic acid
18F-FDG	fluorine-18 fluorodeoxyglucose
53BP1	p53-binding protein 1
AARS2	alanyl-tRNA synthetase 2
ACAT1	acetyl-CoA acetyltransferase 1
ACC	acetyl-CoA carboxylase
ACLY	ATP citrate lyase
ACSL5	acyl-CoA synthetase long-chain family member 5
ACSS2	acyl-CoA synthetase short-chain family member 2
ADI-PEG20	pegylated arginine deiminase
AhR	aryl hydrocarbon receptor
AKT	protein kinase B
α-KG	α-ketoglutarate
α-SMA	α-smooth muscle actin
ALYREF	Aly/REF export factor
AMP	adenosine monophosphate
AMPK	AMP-activated protein kinase
APC	adenomatous polyposis coli
ASNS	asparagine synthetase
ASS1	argininosuccinate synthase 1
ATP	adenosine triphosphate
AUC	area under the curve
AXIN	axis inhibition protein
BBN	N-butyl-N-(4-hydroxybutyl) nitrosamine
BC	bladder cancer
BCG	bacillus Calmette-Guerin
β-TrCP	β-transducin repeat-containing protein
BNIP3	BCL2 interacting protein 3
CAB39	calcium-binding protein 39
CAF	cancer-associated fibroblast
cAMP	cyclic adenosine monophosphate
CBL	Cbl proto-oncogene
CD147	cluster of differentiation 147
CD36	cluster of differentiation 36
CD39	cluster of differentiation 39
CD73	cluster of differentiation 73
CD8	cluster of differentiation 8
CDCA3	cell division cycle-associated protein 3
CDKN1A	cyclin-dependent kinase inhibitor 1A
CDKN3	cyclin-dependent kinase inhibitor 3
CDX	cell-derived xenograft
CI	confidence interval
circRNA	circular RNA
CK1	casein kinase 1
COL1A1	collagen type I alpha 1 chain
COMPASS	complex of proteins associated with Set1
CPT1A	carnitine palmitoyltransferase 1A
CRISPR	clustered regularly interspaced short palindromic repeats
CT	computed tomography
CTGF	connective tissue growth factor
CTLA-4	cytotoxic T-lymphocyte-associated protein 4
CUL4B/RBX1	cullin 4B/RING-box protein 1
CUT&Tag	cleavage under targets and tagmentation
CXCL12	C-X-C motif chemokine ligand 12
CYLD	cylindromatosis
DeltaSUVmean	change in mean standardised uptake value
DHCR7	7-dehydrocholesterol reductase
DLGAP5	discs large-associated protein 5
DNA	deoxyribonucleic acid
DNMT1	DNA methyltransferase 1
E2F	E2F transcription factor family
ENO1	enolase 1
EP300	E1A-binding protein p300
ER	endoplasmic reticulum
FAK	focal adhesion kinase
FAPI	fibroblast-activation-protein inhibitor
FASN	fatty acid synthase
FBXW7	F-box and WD repeat domain-containing 7
FGFR3	fibroblast growth factor receptor 3
FOXP3	forkhead box P3
G3BP1	Ras GTPase-activating protein-binding protein 1
GABPA	GA-binding protein transcription factor subunit alpha
GADD45B	growth arrest and DNA damage-inducible beta
GC–MS	gas chromatography–mass spectrometry
GCN2	general control non-derepressible 2
GLOBOCAN	Global Cancer Observatory
GLS	glutaminase
GLS1	glutaminase 1
GLUD	glutamate dehydrogenase
GLUT1	glucose transporter 1
GPT2	glutamic-pyruvic transaminase 2
GPX4	glutathione peroxidase 4
GSDMD	gasdermin D
GSH	glutathione
GSK3β	glycogen synthase kinase 3 beta
H3K18la	histone H3 lysine 18 lactylation
H3K27me3	histone H3 lysine 27 trimethylation
H3K9la	histone H3 lysine 9 lactylation
H4K5la	histone H4 lysine 5 lactylation
HIF-1α	hypoxia-inducible factor 1 alpha
HILIC	hydrophilic interaction liquid chromatography
HK1	hexokinase 1
HK2	hexokinase 2
hnRNP I/L	heterogeneous nuclear ribonucleoproteins I/L
HR	hazard ratio
HRMS	high-resolution mass spectrometry
HSF1	heat shock factor 1
HSL	hormone-sensitive lipase
HSP90β	heat shock protein 90 beta family member 1
ICI	immune-checkpoint inhibitor
IDH	isocitrate dehydrogenase
IDO1	indoleamine 2,3-dioxygenase 1
IFN-γ	interferon gamma
IGF2BP	insulin-like growth factor 2 mRNA-binding protein
IL	interleukin
IRF3	interferon regulatory factor 3
JAK2	Janus kinase 2
JmjC	Jumonji C domain
JMJD1A	Jumonji domain-containing 1A
KDM6A	lysine demethylase 6A
KDM6B	lysine demethylase 6B
LAG-3	lymphocyte-activation gene 3
LC–MS/MS	liquid chromatography–tandem mass spectrometry
LC3	microtubule-associated protein 1 light chain 3
LCN2	lipocalin 2
LDH	lactate dehydrogenase
LDHA	lactate dehydrogenase A
LDI-MS	laser desorption/ionisation mass spectrometry
LDL	low-density lipoprotein
LIPT1	lipoyltransferase 1
LKB1	liver kinase B1
lncRNA	long non-coding RNA
LRP5/6	low-density lipoprotein receptor-related protein 5/6
LRP8	low-density lipoprotein receptor-related protein 8
m5C	5-methylcytosine
m6A	N6-methyladenosine
MAPK	mitogen-activated protein kinase
MAVS	mitochondrial antiviral-signalling protein
MAX	MYC-associated factor X
MCT1	monocarboxylate transporter 1
MCT4	monocarboxylate transporter 4
MGAT4A	mannosyl-glycoprotein N-acetylglucosaminyltransferase 4A
MHC-I	major histocompatibility complex class I
MIBC	muscle-invasive bladder cancer
miR-30e	microRNA-30e
MLL3/4	mixed-lineage leukaemia proteins 3 and 4
mRNA	messenger RNA
MTCO1	mitochondrially encoded cytochrome c oxidase I
mTOR	mammalian/mechanistic target of rapamycin
mTORC1	mechanistic target of rapamycin complex 1
MYC	MYC proto-oncogene, basic helix-loop-helix transcription factor
NAD+	oxidised nicotinamide adenine dinucleotide
NADPH	nicotinamide adenine dinucleotide phosphate
NDUFB8	NADH:ubiquinone oxidoreductase subunit B8
NF-κB	nuclear factor kappa B
NFAT1	nuclear factor of activated T cells 1
NK	natural killer
NLRP1	NLR family pyrin domain-containing 1
NMIBC	non-muscle-invasive bladder cancer
NMR	nuclear magnetic resonance
NOP2	nucleolar protein 2
ox-LDL	oxidised low-density lipoprotein
OXPHOS	oxidative phosphorylation
P4HA2	prolyl 4-hydroxylase subunit alpha 2
P5C	pyrroline-5-carboxylate
PD-1	programmed cell death protein 1
PD-L1	programmed death-ligand 1
PDH	pyruvate dehydrogenase
PDK	pyruvate dehydrogenase kinase
PDX	patient-derived xenograft
PET/CT	positron-emission tomography/computed tomography
PET/MRI	positron-emission tomography/magnetic-resonance imaging
PFK	phosphofructokinase
PFK1	phosphofructokinase 1
PGK1	phosphoglycerate kinase 1
PGM	phosphoglucomutase
PGM3	phosphoglucomutase 3
PHD2	prolyl hydroxylase domain-containing protein 2
PHGDH	phosphoglycerate dehydrogenase
PI3K	phosphoinositide 3-kinase
PIK3CA	phosphatidylinositol-4,5-bisphosphate 3-kinase catalytic subunit alpha
PKA	protein kinase A
PKM2	pyruvate kinase M2
PPARα	peroxisome proliferator-activated receptor alpha
PPARγ	peroxisome proliferator-activated receptor gamma
PPP	pentose phosphate pathway
PRKN	parkin RBR E3 ubiquitin protein ligase
PSMD11	proteasome 26S subunit, non-ATPase 11
PTEN	phosphatase and tensin homologue
PTHrP	parathyroid hormone-related protein
PYCR1	pyrroline-5-carboxylate reductase 1
RB1	RB transcriptional corepressor 1
RCC	renal cell carcinoma
RIG-I	retinoic acid-inducible gene I
RNA	ribonucleic acid
RNA-seq	RNA sequencing
ROC	receiver operating characteristic
ROS	reactive oxygen species
RUNX2	runt-related transcription factor 2
S6K1	ribosomal protein S6 kinase beta-1
SAM	S-adenosylmethionine
SCD1	stearoyl-CoA desaturase 1
SCO2	synthesis of cytochrome c oxidase 2
SENP3	SUMO-specific peptidase 3
SLC	solute carrier
SLC16A1-AS1	SLC16A1 antisense RNA 1
SOAT1	sterol O-acyltransferase 1
SRC	SRC proto-oncogene, non-receptor tyrosine kinase
SRC-3	steroid receptor coactivator 3
SREBP1	sterol regulatory element-binding protein 1
SRSF1	serine and arginine rich splicing factor 1
STAT3	signal transducer and activator of transcription 3
STING	stimulator of interferon genes
TACO1	translational activator of cytochrome c oxidase I
TAM	tumour-associated macrophage
TCA	tricarboxylic acid
TCF/LEF	T-cell factor/lymphoid enhancer-binding factor
TDO	tryptophan 2,3-dioxygenase
TET	ten-eleven translocation dioxygenase
TGF-β	transforming growth factor beta
TIGAR	TP53-induced glycolysis and apoptosis regulator
TIM-3	T-cell immunoglobulin and mucin domain-containing protein 3
TME	tumour microenvironment
TP53	tumour protein p53
Treg	regulatory T cell
TRIM28	tripartite motif-containing 28
UCA1	urothelial cancer associated 1
UHPLC–UHRMS	ultra-high-performance liquid chromatography–ultra-high-resolution mass spectrometry
UPLC–MS	ultra-performance liquid chromatography–mass spectrometry
USP43	ubiquitin-specific peptidase 43
VDAC1	voltage-dependent anion channel 1
XBP1	X-box-binding protein 1
YAP	Yes-associated protein
YBX1	Y-box binding protein 1
YME1L1	YME1-like 1 ATPase
YTHDC1	YTH domain-containing protein 1
YTHDF2	YTH N6-methyladenosine RNA binding protein 2
YTHDF3	YTH N6-methyladenosine RNA binding protein 3
YY1	Yin Yang 1

## References

[1] Sung H., Filho A.M., Laversanne M., Ferlay J., Siegel R.L., Soerjomataram I., Jemal A., Bray F. (2026). Global cancer statistics 2024: GLOBOCAN estimates of incidence and mortality worldwide for 34 cancers in 186 countries. CA Cancer J. Clin..

[2] Babjuk M., Burger M., Capoun O., Cohen D., Compérat E.M., Dominguez Escrig J.L., Gontero P., Liedberg F., Masson-Lecomte A., Mostafid A.H. (2022). European Association of Urology Guidelines on Non-muscle-invasive Bladder Cancer (Ta, T1, and Carcinoma In Situ). Eur. Urol..

[3] Grossman H.B., Natale R.B., Tangen C.M., Speights V.O., Vogelzang N.J., Trump D.L., DeVere White R.W., Sarosdy M.F., Wood D.P., Raghavan D. (2003). Neoadjuvant chemotherapy plus cystectomy compared with cystectomy alone for locally advanced bladder cancer. N. Engl. J. Med..

[4] Powles T., Park S.H., Voog E., Caserta C., Valderrama B.P., Gurney H., Kalofonos H., Radulović S., Demey W., Ullén A. (2020). Avelumab Maintenance Therapy for Advanced or Metastatic Urothelial Carcinoma. N. Engl. J. Med..

[5] Powles T., Valderrama B.P., Gupta S., Bedke J., Kikuchi E., Hoffman-Censits J., Iyer G., Vulsteke C., Park S.H., Shin S.J. (2024). Enfortumab Vedotin and Pembrolizumab in Untreated Advanced Urothelial Cancer. N. Engl. J. Med..

[6] Xu T., Junge J.A., Delfarah A., Lu Y.T., Arnesano C., Iqbal M., Delijani K., Hsieh T.C., Hodara E., Mehta H.H. (2022). Bladder cancer cells shift rapidly and spontaneously to cisplatin-resistant oxidative phosphorylation that is trackable in real time. Sci. Rep..

[7] Cumberbatch M.G.K., Jubber I., Black P.C., Esperto F., Figueroa J.D., Kamat A.M., Kiemeney L., Lotan Y., Pang K., Silverman D.T. (2018). Epidemiology of Bladder Cancer: A Systematic Review and Contemporary Update of Risk Factors in 2018. Eur. Urol..

[8] Robertson A.G., Kim J., Al-Ahmadie H., Bellmunt J., Guo G., Cherniack A.D., Hinoue T., Laird P.W., Hoadley K.A., Akbani R. (2017). Comprehensive Molecular Characterization of Muscle-Invasive Bladder Cancer. Cell.

[9] Kamoun A., de Reyniès A., Allory Y., Sjödahl G., Robertson A.G., Seiler R., Hoadley K.A., Groeneveld C.S., Al-Ahmadie H., Choi W. (2020). A Consensus Molecular Classification of Muscle-invasive Bladder Cancer. Eur. Urol..

[10] Knowles M.A., Hurst C.D. (2015). Molecular biology of bladder cancer: New insights into pathogenesis and clinical diversity. Nat. Rev. Cancer.

[11] Hanahan D. (2022). Hallmarks of Cancer: New Dimensions. Cancer Discov..

[12] Groeneveld C.S., Sanchez-Quiles V., Dufour F., Shi M., Dingli F., Nicolle R., Chapeaublanc E., Poullet P., Jeffery D., Krucker C. (2024). Proteogenomic Characterization of Bladder Cancer Reveals Sensitivity to Apoptosis Induced by Tumor Necrosis Factor-related Apoptosis-inducing Ligand in FGFR3-mutated Tumors. Eur. Urol..

[13] Burns J.E., Hurst C.D., Knowles M.A., Phillips R.M., Allison S.J. (2021). The Warburg effect as a therapeutic target for bladder cancers and intratumoral heterogeneity in associated molecular targets. Cancer Sci..

[14] Yao Z., Xu N., Shang G., Wang H., Tao H., Wang Y., Qin Z., Tan S., Feng J., Zhu J. (2023). Proteogenomics of different urothelial bladder cancer stages reveals distinct molecular features for papillary cancer and carcinoma in situ. Nat. Commun..

[15] Du X., Wang Q.R., Chan E., Merchant M., Liu J., French D., Ashkenazi A., Qing J. (2012). FGFR3 stimulates stearoyl CoA desaturase 1 activity to promote bladder tumor growth. Cancer Res..

[16] Kawahara I., Yoshino H., Fukumoto W., Arima J., Saito S., Li G., Fukuda I., Mitsuke A., Sakaguchi T., Inoguchi S. (2024). Targeting metabolic reprogramming to overcome drug resistance in advanced bladder cancer: Insights from gemcitabine- and cisplatin-resistant models. Mol. Oncol..

[17] King M.A., Ganley I.G., Flemington V. (2016). Inhibition of cholesterol metabolism underlies synergy between mTOR pathway inhibition and chloroquine in bladder cancer cells. Oncogene.

[18] Christoph F., Schmidt B., Schmitz-Dräger B.J., Schulz W.A. (1999). Over-expression and amplification of the c-myc gene in human urothelial carcinoma. Int. J. Cancer.

[19] Deng Z., Zhou F., Li M., Jin W., Yu J., Wang G., Qian K., Ju L., Zhang Y., Xiao Y. (2025). DLGAP5 enhances bladder cancer chemoresistance by regulating glycolysis through MYC stabilization. Theranostics.

[20] Liu S., Liu X., Liu G., Wang Z., Li C., Yu M., Yao J., Xing H., Zhu Y. (2026). Nuclear hexokinase 2 couples hyperglycemia to MYC-driven glycolytic and stemness programs in bladder cancer. Cell Death Dis..

[21] Afonso J., Barbosa-Matos C., Silvestre R., Pereira-Vieira J., Gonçalves S.M., Mendes-Alves C., Parpot P., Pinto J., Carapito Â., Guedes de Pinho P. (2024). Cisplatin-Resistant Urothelial Bladder Cancer Cells Undergo Metabolic Reprogramming beyond the Warburg Effect. Cancers.

[22] Afonso J., Gonçalves C., Costa M., Ferreira D., Santos L., Longatto-Filho A., Baltazar F. (2023). Glucose Metabolism Reprogramming in Bladder Cancer: Hexokinase 2 (HK2) as Prognostic Biomarker and Target for Bladder Cancer Therapy. Cancers.

[23] Pan M., Tao T., Kong D., Gong H. (2026). Aerobic glycolysis in bladder cancer: Research advances and targeted therapy potential. Front. Oncol..

[24] Li M., Yu J., Ju L., Wang Y., Jin W., Zhang R., Xiang W., Ji M., Du W., Wang G. (2024). USP43 stabilizes c-Myc to promote glycolysis and metastasis in bladder cancer. Cell Death Dis..

[25] Shen D., Yu X., Fan X., Liang Y., Lu D., Ke Z., Wang L., Xiang P., Xiao J. (2025). CDCA3-MYC positive feedback loop promotes bladder cancer progression via ENO1-mediated glycolysis. J. Exp. Clin. Cancer Res. CR.

[26] Özden-Yılmaz G., Savas B., Bursalı A., Eray A., Arıbaş A., Senturk S., Karaca E., Karakülah G., Erkek-Ozhan S. (2023). Differential Occupancy and Regulatory Interactions of KDM6A in Bladder Cell Lines. Cells.

[27] Chang S., Yim S., Park H. (2019). The cancer driver genes IDH1/2, JARID1C/KDM5C, and UTX/KDM6A: Crosstalk between histone demethylation and hypoxic reprogramming in cancer metabolism. Exp. Mol. Med..

[28] Loras A., Segovia C., Ruiz-Cerdá J.L. (2021). Epigenomic and Metabolomic Integration Reveals Dynamic Metabolic Regulation in Bladder Cancer. Cancers.

[29] Yang J., Xu J., Wang W., Zhang B., Yu X., Shi S. (2023). Epigenetic regulation in the tumor microenvironment: Molecular mechanisms and therapeutic targets. Signal Transduct. Target. Ther..

[30] Martinez V.G., Munera-Maravilla E., Bernardini A., Rubio C., Suarez-Cabrera C., Segovia C., Lodewijk I., Dueñas M., Martínez-Fernández M., Paramio J.M. (2019). Epigenetics of Bladder Cancer: Where Biomarkers and Therapeutic Targets Meet. Front. Genet..

[31] Singh P., D’Rozario R., Chakraborty B., Meher S., Raychaudhuri D., Tannir A.J., Li Y., Majumdar A., Hawkins J., Xiong Y. (2026). Loss of KDM6A-mediated genomic instability and metabolic reprogramming regulates response to therapeutic perturbations in bladder cancer. Nat. Commun..

[32] Liu L., Cui J., Zhao Y., Liu X., Chen L., Xia Y., Wang Y., Chen S., Sun S., Shi B. (2021). KDM6A-ARHGDIB axis blocks metastasis of bladder cancer by inhibiting Rac1. Mol. Cancer.

[33] Wang G., Peng T., Chen L., Xiong K., Ju L., Qian K., Zhang Y., Xiao Y., Wang X. (2024). Mevalonate pathway inhibition reduces bladder cancer metastasis by modulating RhoB protein stability and integrin β1 localization. Commun. Biol..

[34] Pelaz S.G., Tabernero A. (2022). Src: Coordinating metabolism in cancer. Oncogene.

[35] Zhou L., Du K., Dai Y., Zeng Y., Luo Y., Ren M., Pan W., Liu Y., Zhang L., Zhu R. (2024). Metabolic reprogramming based on RNA sequencing of gemcitabine-resistant cells reveals the FASN gene as a therapeutic for bladder cancer. J. Transl. Med..

[36] Hart M., Concordet J.P., Lassot I., Albert I., del los Santos R., Durand H., Perret C., Rubinfeld B., Margottin F., Benarous R. (1999). The F-box protein beta-TrCP associates with phosphorylated beta-catenin and regulates its activity in the cell. Curr. Biol. CB.

[37] Zeng X., Tamai K., Doble B., Li S., Huang H., Habas R., Okamura H., Woodgett J., He X. (2005). A dual-kinase mechanism for Wnt co-receptor phosphorylation and activation. Nature.

[38] Li Y., Zheng Y., Izumi K., Ishiguro H., Ye B., Li F., Miyamoto H. (2013). Androgen activates β-catenin signaling in bladder cancer cells. Endocr.-Relat. Cancer.

[39] Chen W., Zhou J., Wu K., Huang J., Ding Y., Yun E.J., Wang B., Ding C., Hernandez E., Santoyo J. (2016). Targeting XBP1-mediated β-catenin expression associated with bladder cancer with newly synthetic Oridonin analogues. Oncotarget.

[40] Zhao Y., Shi G., Huang X., Zhang Z., Liao K., Xiong H., Feng Z., Mao S., Zhang X. (2025). LRP8 inhibits bladder cancer cell ferroptosis by activating the Wnt/β-catenin-SCD1 positive feedback loop. Hum. Mol. Genet..

[41] Dupont S., Morsut L., Aragona M., Enzo E., Giulitti S., Cordenonsi M., Zanconato F., Le Digabel J., Forcato M., Bicciato S. (2011). Role of YAP/TAZ in mechanotransduction. Nature.

[42] Calvo F., Ege N., Grande-Garcia A., Hooper S., Jenkins R.P., Chaudhry S.I., Harrington K., Williamson P., Moeendarbary E., Charras G. (2013). Mechanotransduction and YAP-dependent matrix remodelling is required for the generation and maintenance of cancer-associated fibroblasts. Nat. Cell Biol..

[43] Tao Y., Huang J., Hou J., Hu Z., Zhang T., Mo Z., Zeng K., Wu J., Wang D., Qiu J. (2025). Matrix stiffness maintains bladder cancer stemness via integrin-nuclear skeleton axis. Cell Death Dis..

[44] Vasseur S., Guillaumond F. (2022). Lipids in cancer: A global view of the contribution of lipid pathways to metastatic formation and treatment resistance. Oncogenesis.

[45] Zhang C., Zhu N., Li H., Gong Y., Gu J., Shi Y., Liao D., Wang W., Dai A., Qin L. (2022). New dawn for cancer cell death: Emerging role of lipid metabolism. Mol. Metab..

[46] Matoba S., Kang J.G., Patino W.D., Wragg A., Boehm M., Gavrilova O., Hurley P.J., Bunz F., Hwang P.M. (2006). P53 regulates mitochondrial respiration. Science.

[47] Bensaad K., Tsuruta A., Selak M.A., Vidal M.N., Nakano K., Bartrons R., Gottlieb E., Vousden K.H. (2006). TIGAR, a p53-inducible regulator of glycolysis and apoptosis. Cell.

[48] Logotheti S., Marquardt S., Gupta S.K., Richter C., Edelhäuser B.A.H., Engelmann D., Brenmoehl J., Söhnchen C., Murr N., Alpers M. (2020). LncRNA-SLC16A1-AS1 induces metabolic reprogramming during Bladder Cancer progression as target and co-activator of E2F1. Theranostics.

[49] Ding Y., Jin K., Su X., Zhang L., Liu Z., Sun J., Wu Y., Shi J., Zeng H., Liu H. (2026). Aberrant p53 fuels macrophage immunoregulatory polarization and refractory clinical outcome in urothelial carcinoma. J. Immunother. Cancer.

[50] Düvel K., Yecies J.L., Menon S., Raman P., Lipovsky A.I., Souza A.L., Triantafellow E., Ma Q., Gorski R., Cleaver S. (2010). Activation of a metabolic gene regulatory network downstream of mTOR complex 1. Mol. Cell.

[51] Kami Reddy K.R., Putluri V., Piyarathna D.W.B., Park J.H., Arun A.K., Jorvekar S.B., Dou Y., Hassan M.K., Amara C.S., Kamal A.H.M. (2026). Oncogenic PIK3CA reprograms glutamine metabolism to drive bladder cancer progression. Cell Commun. Signal.

[52] Zhang D., Tang Z., Huang H., Zhou G., Cui C., Weng Y., Liu W., Kim S., Lee S., Perez-Neut M. (2019). Metabolic regulation of gene expression by histone lactylation. Nature.

[53] Chen Y., Xu J., Liu X., Guo L., Yi P., Cheng C. (2023). Potential therapies targeting nuclear metabolic regulation in cancer. MedComm.

[54] Wan W., Peng K., Li M., Qin L., Tong Z., Yan J., Shen B., Yu C. (2017). Histone demethylase JMJD1A promotes urinary bladder cancer progression by enhancing glycolysis through coactivation of hypoxia inducible factor 1α. Oncogene.

[55] Wang J.Z., Zhu W., Han J., Yang X., Zhou R., Lu H.C., Yu H., Yuan W.B., Li P.C., Tao J. (2021). The role of the HIF-1α/ALYREF/PKM2 axis in glycolysis and tumorigenesis of bladder cancer. Cancer Commun..

[56] Cheng C., Gao J., Wang R., Wu Y., Wang Y., Jin B., Fu G. (2026). NOP2-mediated 5-methylcytosine Regulates Lipid Metabolism Reprogramming to Prime Tumors for Ferroptosis in Bladder Cancer Progression. Int. J. Biol. Sci..

[57] Wang Z., Zhou Z., Yang S., Zhao Z., Li X., Liu X., Cheng G., Xu R., Li Q., Xing D. (2026). Glycolysis-mediated H3K18la modifications drive aggressive bladder cancer through metabolic and epigenetic reprogramming. Front. Immunol..

[58] Li Z., Jiang Q., Yang Q., Zhou Y., Wang J. (2025). Hypoxia-induced PYCR1 regulates glycolysis and histone lactylation to promote bladder cancer progression and metastasis via SLC6A14/Glutamine metabolism. Cancer Biol. Ther..

[59] Dinh T.J., Rogg M., Cosenza-Contreras M., Li M., Zirngibl M., Pinter N., Kurowski K., Hause F., Pauli L., Imberg F. (2025). Proteomic analysis of non-muscle invasive and muscle invasive bladder cancer highlights distinct subgroups with metabolic, matrisomal, and immune hallmarks and emphasizes importance of the stromal compartment. J. Pathol..

[60] Sahu D., Lotan Y., Wittmann B., Neri B., Hansel D.E. (2017). Metabolomics analysis reveals distinct profiles of nonmuscle-invasive and muscle-invasive bladder cancer. Cancer Med..

[61] Zhang Y., Zhu B., Cai Y., Zhu S., Zhao H., Ying X., Jiang C., Zeng J. (2022). Alteration in glycolytic/cholesterogenic gene expression is associated with bladder cancer prognosis and immune cell infiltration. BMC Cancer.

[62] Tate T., Xiang T., Wobker S.E., Zhou M., Chen X., Kim H., Batourina E., Lin C.S., Kim W.Y., Lu C. (2021). Pparg signaling controls bladder cancer subtype and immune exclusion. Nat. Commun..

[63] Ramos J.R., Pabijan J., Garcia R., Lekka M. (2014). The softening of human bladder cancer cells happens at an early stage of the malignancy process. Beilstein J. Nanotechnol..

[64] Ghasemi H., Mousavibahar S.H., Hashemnia M., Karimi J., Khodadadi I., Mirzaei F., Tavilani H. (2020). Tissue stiffness contributes to YAP activation in bladder cancer patients undergoing transurethral resection. Ann. N. Y. Acad. Sci..

[65] Ma M., Li J., Li X., Jing M., Wang L., Jiang Y., Yang Z., He J., Wang M., Liu H. (2025). Piezo1/ITGB1 Synergizes With Ca(2+)/YAP Signaling to Propel Bladder Carcinoma Progression via a Stiffness-Dependent Positive Feedback Loop. Cancer Med..

[66] Dai M., Yuan X., Sun C., Han R., Björkholm M., Kong F., Zhao S., Xu D. (2025). The ETS transcription factor GABPA inhibits bladder cancer aggressiveness by repressing extracellular matrix deposition and mechanotransduction signaling. Cell Death Dis..

[67] Metwally S., Gil D., Śmiałek-Bartyzel J., Pabijan J., Wątor G., Gnanachandran K., Berardi M., Kozłowski Ł., Navdeep, Lekka M. (2026). Matrix Mechanics Governs Mechano-Metabolic Adaptation across Cancer Grades in Bladder Spheroids. ACS Appl. Mater. Interfaces.

[68] Dong X., Chen Z., Chen Y., Xiao Z., Zhang J., Li Y., Du H. (2026). Mechanical stress, metabolic reprogramming and stromal remodeling: An emerging paradigm in the bladder cancer mechano-micro-environment. Front. Genet..

[69] Metwally S., Śmiałek-Bartyzel J., Pabijan J., Lekka M. (2025). Structural and Mechanical Characterization of Collagen-Hyaluronan Hydrogels Used to Study Cancer Cell Invasion through the Bladder Wall. ACS Biomater. Sci. Eng..

[70] Jang C., Chen L., Rabinowitz J.D. (2018). Metabolomics and Isotope Tracing. Cell.

[71] Xia Y., Wang X., Liu Y., Shapiro E., Lepor H., Tang M.S., Sun T.T., Wu X.R. (2022). PKM2 Is Essential for Bladder Cancer Growth and Maintenance. Cancer Res..

[72] Christofk H.R., Vander Heiden M.G., Harris M.H., Ramanathan A., Gerszten R.E., Wei R., Fleming M.D., Schreiber S.L., Cantley L.C. (2008). The M2 splice isoform of pyruvate kinase is important for cancer metabolism and tumour growth. Nature.

[73] Woolbright B.L., Choudhary D., Mikhalyuk A., Trammel C., Shanmugam S., Abbott E., Pilbeam C.C., Taylor J.A. (2018). The Role of Pyruvate Dehydrogenase Kinase-4 (PDK4) in Bladder Cancer and Chemoresistance. Mol. Cancer Ther..

[74] Woolbright B.L., Rajendran G., Abbott E., Martin A., Didde R., Dennis K., Harris R.A., Taylor J.A. (2023). Pyruvate Dehydrogenase Kinase 4 Deficiency Increases Tumorigenesis in a Murine Model of Bladder Cancer. Cancers.

[75] Zhao W., Chang C., Cui Y., Zhao X., Yang J., Shen L., Zhou J., Hou Z., Zhang Z., Ye C. (2014). Steroid receptor coactivator-3 regulates glucose metabolism in bladder cancer cells through coactivation of hypoxia inducible factor 1α. J. Biol. Chem..

[76] Zuo Y., Ren D., He H., Huang C., Zhu X. (2025). CircST6GALNAC6 Inhibits Glycolysis of Bladder Cancer by Regulating PRKN/HK1 Signaling Pathway. Mol. Carcinog..

[77] Cheng Y., Chen T., Zheng G., Song Z., Zhang G., Xiao S., Rao X., Zeng T. (2026). PSMD11 stabilizes PGM3 by antagonizing Parkin to promote bladder cancer progression through energy metabolism reprogramming. Cell Death Dis..

[78] Kirk P., Wilson M.C., Heddle C., Brown M.H., Barclay A.N., Halestrap A.P. (2000). CD147 is tightly associated with lactate transporters MCT1 and MCT4 and facilitates their cell surface expression. EMBO J..

[79] Choi J.W., Kim Y., Lee J.H., Kim Y.S. (2014). Prognostic significance of lactate/proton symporters MCT1, MCT4, and their chaperone CD147 expressions in urothelial carcinoma of the bladder. Urology.

[80] Kong L., Yang G., Weng B., Wen Z., Kaggie J.D., Cai Q., Wan Q., Zheng C., Du F., Li Y. (2026). Early Detection of Chemotherapy-Induced Glucose Metabolic Alterations in Bladder Carcinoma Using Deuterium ((2)H) Metabolic Imaging: In Vitro and in Vivo Assessments. Adv. Sci..

[81] Tao T., Su Q., Xu S., Deng J., Zhou S., Zhuang Y., Huang Y., He C., He S., Peng M. (2019). Down-regulation of PKM2 decreases FASN expression in bladder cancer cells through AKT/mTOR/SREBP-1c axis. J. Cell. Physiol..

[82] Zeng F., Lan Y., Wang N., Huang X., Zhou Q., Wang Y. (2022). Ferroptosis: A new therapeutic target for bladder cancer. Front. Pharmacol..

[83] Yang L., Sun J., Li M., Long Y., Zhang D., Guo H., Huang R., Yan J. (2021). Oxidized Low-Density Lipoprotein Links Hypercholesterolemia and Bladder Cancer Aggressiveness by Promoting Cancer Stemness. Cancer Res..

[84] Fredericks W.J., McGarvey T., Wang H., Lal P., Puthiyaveettil R., Tomaszewski J., Sepulveda J., Labelle E., Weiss J.S., Nickerson M.L. (2011). The bladder tumor suppressor protein TERE1 (UBIAD1) modulates cell cholesterol: Implications for tumor progression. DNA Cell Biol..

[85] Gao M., Li C., Li W., Xie J., Long J., Luo M., Hu J., Lai C., Lan T., Zhu D. (2026). White-to-brown adipose switching promotes bladder cancer progression. Neoplasia.

[86] Luo Y., Zeng Y., Liu Y., Liu S., Dai Y., Pan W., Zhang L., Zhu R., Feng D., Du K. (2025). ACSL5 regulated acetyl-CoA to promote bladder cancer cellular senescence via 53BP1 acetylation. Oncogene.

[87] Du K., Luo Y., Zhang L., Zeng Y., Dai Y., Ren M., Pan W., Liu Y., Tian F., Zhou L. (2024). m(6)A modification of lipoyltransferase 1 inhibits bladder cancer progression by activating cuproptosis. Oncogene.

[88] Mullen A.R., Wheaton W.W., Jin E.S., Chen P.H., Sullivan L.B., Cheng T., Yang Y., Linehan W.M., Chandel N.S., DeBerardinis R.J. (2011). Reductive carboxylation supports growth in tumour cells with defective mitochondria. Nature.

[89] Sun N., Liang Y., Chen Y., Wang L., Li D., Liang Z., Sun L., Wang Y., Niu H. (2019). Glutamine affects T24 bladder cancer cell proliferation by activating STAT3 through ROS and glutaminolysis. Int. J. Mol. Med..

[90] Zhao H., Wu W., Li X., Chen W. (2022). Long noncoding RNA UCA1 promotes glutamine-driven anaplerosis of bladder cancer by interacting with hnRNP I/L to upregulate GPT2 expression. Transl. Oncol..

[91] Huang Z., Liu B., Li X., Jin C., Hu Q., Zhao Z., Sun Y., Wang Q. (2025). RUNX2 enhances bladder cancer progression by promoting glutamine metabolism. Neoplasia.

[92] Muhammad N., Lee H.M., Kim J. (2020). Oncology Therapeutics Targeting the Metabolism of Amino Acids. Cells.

[93] Allen M.D., Luong P., Hudson C., Leyton J., Delage B., Ghazaly E., Cutts R., Yuan M., Syed N., Lo Nigro C. (2014). Prognostic and therapeutic impact of argininosuccinate synthetase 1 control in bladder cancer as monitored longitudinally by PET imaging. Cancer Res..

[94] Du S., Sui Y., Ren W., Zhou J., Du C. (2021). PYCR1 promotes bladder cancer by affecting the Akt/Wnt/β-catenin signaling. J. Bioenerg. Biomembr..

[95] Li Z., Liu J., Fu H., Li Y., Liu Q., Song W., Zeng M. (2022). SENP3 affects the expression of PYCR1 to promote bladder cancer proliferation and EMT transformation by deSUMOylation of STAT3. Aging.

[96] Kay E.J., Paterson K., Riera-Domingo C., Sumpton D., Däbritz J.H.M., Tardito S., Boldrini C., Hernandez-Fernaud J.R., Athineos D., Dhayade S. (2022). Cancer-associated fibroblasts require proline synthesis by PYCR1 for the deposition of pro-tumorigenic extracellular matrix. Nat. Metab..

[97] Wei W., Li H., Tian S., Zhang C., Liu J., Tao W., Cai T., Dong Y., Wang C., Lu D. (2025). Asparagine drives immune evasion in bladder cancer via RIG-I stability and type I IFN signaling. J. Clin. Investig..

[98] Lee S.H., Mahendran R., Tham S.M., Thamboo T.P., Chionh B.J., Lim Y.X., Tsang W.C., Wu Q.H., Chia J.Y., Tay M.H.W. (2021). Tryptophan-kynurenine ratio as a biomarker of bladder cancer. BJU Int..

[99] Badawy A.A., Guillemin G. (2019). The Plasma [Kynurenine]/[Tryptophan] Ratio and Indoleamine 2,3-Dioxygenase: Time for Appraisal. Int. J. Tryptophan Res. IJTR.

[100] Ma Z., Li Z., Mao Y., Ye J., Liu Z., Wang Y., Wei C., Cui J., Liu Z., Liang X. (2023). AhR diminishes the efficacy of chemotherapy via suppressing STING dependent type-I interferon in bladder cancer. Nat. Commun..

[101] Deng M., Zhou Z., Chen J., Li X., Liu Z., Ye J., Wei W., Wang N., Peng Y., Luo X. (2025). Enhanced Oxidative Phosphorylation Driven by TACO1 Mitochondrial Translocation Promotes Stemness and Cisplatin Resistance in Bladder Cancer. Adv. Sci..

[102] Gao D., Wang R., Gong Y., Yu X., Niu Q., Yang E., Fan G., Ma J., Chen C., Tao Y. (2023). CAB39 promotes cisplatin resistance in bladder cancer via the LKB1-AMPK-LC3 pathway. Free Radic. Biol. Med..

[103] Wu Q., Yu H., Sun H., Lv J., Zhuang J., Cai L., Jiang L., Chen Y., Tao Y., Bai K. (2025). SRSF1-mediated alternative splicing regulates bladder cancer progression and cisplatin sensitivity through HIF1A/BNIP3/mitophagy axis. J. Transl. Med..

[104] Kami Reddy K.R., Piyarathna D.W.B., Park J.H., Putluri V., Amara C.S., Kamal A.H.M., Xu J., Kraushaar D., Huang S., Jung S.Y. (2024). Mitochondrial reprogramming by activating OXPHOS via glutamine metabolism in African American patients with bladder cancer. JCI Insight.

[105] Yang E., Jing S., Wang F., Wang H., Fu S., Yang L., Tian J., Golijanin D.J., El-Deiry W.S., Cheng L. (2024). Mesenchymal stem cells in tumor microenvironment: Drivers of bladder cancer progression through mitochondrial dynamics and energy production. Cell Death Dis..

[106] Wang K.Y., Wang K.J., Shen L.L., Wang X.H. (2024). The down-regulation of GADD45B leads to a conversion of cellular oxidative phosphorylation to glycolysis and promotes the progression of bladder cancer. Heliyon.

[107] Alhozeel B., Pandey S.K., Shteinfer-Kuzmine A., Santhanam M., Shoshan-Barmatz V. (2024). Silencing the Mitochondrial Gatekeeper VDAC1 as a Potential Treatment for Bladder Cancer. Cells.

[108] Sun L., Yao Y., Gong S., Chen Z., Xie W., Wang L., Liu X. (2026). YME1L1 degradation by TRIM21 inhibits bladder cancer proliferation, metastasis, and mitochondrial function. Oncogene.

[109] Bondaruk J., Jaksik R., Wang Z., Cogdell D., Lee S., Chen Y., Dinh K.N., Majewski T., Zhang L., Cao S. (2022). The origin of bladder cancer from mucosal field effects. iScience.

[110] Majewski T., Yao H., Bondaruk J., Chung W., Lee S., Lee J.G., Zhang S., Cogdell D., Yang G., Choi W. (2019). Whole-Organ Genomic Characterization of Mucosal Field Effects Initiating Bladder Carcinogenesis. Cell Rep..

[111] Li M., Che N., Jin Y., Li J., Yang W. (2022). CDKN3 Overcomes Bladder Cancer Cisplatin Resistance via LDHA-Dependent Glycolysis Reprogramming. OncoTargets Ther..

[112] Yu S.Y., Li H.Y., Wang C.Y., Wang W.J., Hsu S.H., Hsiang M.C., Yee K.X., Lee K.H., Lee Y.C. (2025). Tumor-derived lactate fuels the STAT3-LCN2 pathway to promote bladder cancer malignancy and chemoresistance. Sci. Rep..

[113] Briceño P., Rivas-Yañez E., Rosemblatt M.V., Parra-Tello B., Farías P., Vargas L., Simon V., Cárdenas C., Lladser A., Salazar-Onfray F. (2021). CD73 Ectonucleotidase Restrains CD8+ T Cell Metabolic Fitness and Anti-tumoral Activity. Front. Cell Dev. Biol..

[114] Izawa M., Tanaka N., Murakami T., Anno T., Teranishi Y., Takamatsu K., Mikami S., Kakimi K., Imamura T., Matsumoto K. (2023). Single-Cell Phenotyping of CD73 Expression Reveals the Diversity of the Tumor Immune Microenvironment and Reflects the Prognosis of Bladder Cancer. Lab. Investig. A J. Tech. Methods Pathol..

[115] Furriel F., Laranjeira P., Pereira M., Silva S., Silva I., Fontinha G., Sousa V., Gomes C., Parada B., Paiva A. (2026). CD39/CD73-mediated immunosuppression and tumor aggressiveness in bladder cancer. Cancer Immunol. Immunother. CII.

[116] Hesse J., Steckel B., Dieterich P., Aydin S., Deussen A., Schrader J. (2024). Intercellular crosstalk shapes purinergic metabolism and signaling in cancer cells. Cell Rep..

[117] Santos H., Matheus L.H.G., Silva A., Dalmazzo S.V., Santos A.A., Santos L., Souza D.M., Reis S.T., Nascimento I.P., Dellê H. (2022). Indoleamine 2,3-Dioxygenase-1 Expression is Changed During Bladder Cancer Cell Invasion. Int. J. Tryptophan Res. IJTR.

[118] Chen R., Su Y., Chen J., Zhu S., Chen X., Su Y. (2026). TRIM28-mediated SUMOylation of SREBF1 drives PD-L1 N-glycosylation and immune evasion in bladder cancer. Cell Death Dis..

[119] Scholtes M.P., de Jong F.C., Zuiverloon T.C.M., Theodorescu D. (2021). Role of Bladder Cancer Metabolic Reprogramming in the Effectiveness of Immunotherapy. Cancers.

[120] Watson M.J., Delgoffe G.M. (2022). Fighting in a wasteland: Deleterious metabolites and antitumor immunity. J. Clin. Investig..

[121] Wang Y., Wang Y., Ren Y., Zhang Q., Yi P., Cheng C. (2022). Metabolic modulation of immune checkpoints and novel therapeutic strategies in cancer. Semin. Cancer Biol..

[122] Strauss L., Guarneri V., Gennari A., Sica A. (2021). Implications of metabolism-driven myeloid dysfunctions in cancer therapy. Cell. Mol. Immunol..

[123] Shi R., Tang Y.Q., Miao H. (2020). Metabolism in tumor microenvironment: Implications for cancer immunotherapy. MedComm.

[124] Günther J., Däbritz J., Wirthgen E. (2019). Limitations and Off-Target Effects of Tryptophan-Related IDO Inhibitors in Cancer Treatment. Front. Immunol..

[125] Lv J., Li K., Yu H., Han J., Zhuang J., Yu R., Cheng Y., Song Q., Bai K., Cao Q. (2023). HNRNPL induced circFAM13B increased bladder cancer immunotherapy sensitivity via inhibiting glycolysis through IGF2BP1/PKM2 pathway. J. Exp. Clin. Cancer Res. CR.

[126] Xiong Q., Feng D., Wang Z., Ying Y., Xu C., Wei Q., Zeng S., Yang L. (2022). Fatty Acid Synthase Is the Key Regulator of Fatty Acid Metabolism and Is Related to Immunotherapy in Bladder Cancer. Front. Immunol..

[127] Li K., Lv J., Wang J., Wei Y., Zhang Y., Lin J., Zhu Q. (2024). CircZNF609 inhibited bladder cancer immunotherapy sensitivity via enhancing fatty acid uptake through IGF2BP2/CD36 pathway. Int. Immunopharmacol..

[128] Zhuang J., Shen L., Li M., Sun J., Hao J., Li J., Zhu Z., Ge S., Zhang D., Guo H. (2023). Cancer-Associated Fibroblast-Derived miR-146a-5p Generates a Niche That Promotes Bladder Cancer Stemness and Chemoresistance. Cancer Res..

[129] Russo M., Chen M., Mariella E., Peng H., Rehman S.K., Sancho E., Sogari A., Toh T.S., Balaban N.Q., Batlle E. (2024). Cancer drug-tolerant persister cells: From biological questions to clinical opportunities. Nat. Rev. Cancer.

[130] Marine J.C., Dawson S.J., Dawson M.A. (2020). Non-genetic mechanisms of therapeutic resistance in cancer. Nat. Rev. Cancer.

[131] Baek S.W., Yoon S.Y., Kim S.K., Leem S.H. (2026). Therapy-Driven Molecular Evolution of Bladder Cancer: Roles of Cellular Plasticity and Tumor Microenvironment. Int. J. Mol. Sci..

[132] Jin M., Cai Z., Bu M., Liu J., Zhang X. (2026). From molecular networks to translational intervention: Current progress in the mechanisms of gemcitabine resistance in bladder cancer. Front. Immunol..

[133] Gong Y., Gao D., Shi Y., Fan G., Yu X., Yang E., Cheng H., Tian J., Ding H., Liu S. (2025). SRC enhanced cisplatin resistance in bladder cancer by reprogramming glycolysis and pentose phosphate pathway. Commun. Biol..

[134] Shigeta K., Hasegawa M., Hishiki T., Naito Y., Baba Y., Mikami S., Matsumoto K., Mizuno R., Miyajima A., Kikuchi E. (2023). IDH2 stabilizes HIF-1α-induced metabolic reprogramming and promotes chemoresistance in urothelial cancer. EMBO J..

[135] Wen H., Lee S., Zhu W.G., Lee O.J., Yun S.J., Kim J., Park S. (2019). Glucose-derived acetate and ACSS2 as key players in cisplatin resistance in bladder cancer. Biochim. Biophys. Acta. Mol. Cell Biol. Lipids.

[136] Peng T., Xiong K., He Z., Cheng S., Chen S., Chen S., Yang Z., Xiang W., Ju L., Zhang Y. (2024). Acyl-coenzyme A: Cholesterol acyltransferase inhibitor avasimibe suppresses tumorigenesis and induces G1-phase cell-cycle arrest by activating PPARγ signaling pathway in bladder cancer. J. Cancer.

[137] Zeng Y., Luo Y., Zhao K., Liu S., Wu K., Wu Y., Du K., Pan W., Dai Y., Liu Y. (2024). M6A-Mediated Induction of 7-Dehydrocholesterol Reductase Stimulates Cholesterol Synthesis and cAMP Signaling to Promote Bladder Cancer Metastasis. Cancer Res..

[138] Li F., Zhang H., Huang Y., Li D., Zheng Z., Xie K., Cao C., Wang Q., Zhao X., Huang Z. (2024). Single-cell transcriptome analysis reveals the association between histone lactylation and cisplatin resistance in bladder cancer. Drug Resist. Updat. Rev. Comment. Antimicrob. Anticancer Chemother..

[139] Yu K., Sun J., Zhang J., Shi Y., Wang J., Wu Y., Lu D., Niu X., Li Y., Huang S. (2026). Lactylation enhances YTHDF3 stability to promote cisplatin resistance via m6A-dependent KDM6B decay in bladder cancer. Cancer Lett..

[140] Su Y., Wang B., Huang J., Huang M., Lin T. (2023). YTHDC1 positively regulates PTEN expression and plays a critical role in cisplatin resistance of bladder cancer. Cell Prolif..

[141] Witjes J.A., Bruins H.M., Cathomas R., Compérat E.M., Cowan N.C., Gakis G., Hernández V., Linares Espinós E., Lorch A., Neuzillet Y. (2021). European Association of Urology Guidelines on Muscle-invasive and Metastatic Bladder Cancer: Summary of the 2020 Guidelines. Eur. Urol..

[142] Sharma P., Goswami S., Raychaudhuri D., Siddiqui B.A., Singh P., Nagarajan A., Liu J., Subudhi S.K., Poon C., Gant K.L. (2023). Immune checkpoint therapy-current perspectives and future directions. Cell.

[143] Kumagai S., Koyama S., Itahashi K., Tanegashima T., Lin Y.T., Togashi Y., Kamada T., Irie T., Okumura G., Kono H. (2022). Lactic acid promotes PD-1 expression in regulatory T cells in highly glycolytic tumor microenvironments. Cancer Cell.

[144] Noe J.T., Rendon B.E., Geller A.E., Conroy L.R., Morrissey S.M., Young L.E.A., Bruntz R.C., Kim E.J., Wise-Mitchell A., Barbosa de Souza Rizzo M. (2021). Lactate supports a metabolic-epigenetic link in macrophage polarization. Sci. Adv..

[145] Yu Y., Liang Y., Xie F., Zhang Z., Zhang P., Zhao X., Zhang Z., Liang Z., Li D., Wang L. (2024). Tumor-associated macrophage enhances PD-L1-mediated immune escape of bladder cancer through PKM2 dimer-STAT3 complex nuclear translocation. Cancer Lett..

[146] Scharping N.E., Menk A.V., Moreci R.S., Whetstone R.D., Dadey R.E., Watkins S.C., Ferris R.L., Delgoffe G.M. (2016). The Tumor Microenvironment Represses T Cell Mitochondrial Biogenesis to Drive Intratumoral T Cell Metabolic Insufficiency and Dysfunction. Immunity.

[147] Tang S., Liu Y., Jin B., Zhang T., Wang X., Zhao J., Xu Z., Zhao W., Bian H., Li K. (2026). LDHA facilitates immune escape in bladder cancer through H4K5 lactylation-dependent upregulation of PD-L1. Biochem. Pharmacol..

[148] Xie B., Lin J., Chen X., Zhou X., Zhang Y., Fan M., Xiang J., He N., Hu Z., Wang F. (2023). CircXRN2 suppresses tumor progression driven by histone lactylation through activating the Hippo pathway in human bladder cancer. Mol. Cancer.

[149] Ma X., Bi E., Lu Y., Su P., Huang C., Liu L., Wang Q., Yang M., Kalady M.F., Qian J. (2019). Cholesterol Induces CD8(+) T Cell Exhaustion in the Tumor Microenvironment. Cell Metab..

[150] Wang C., He H., Fang W. (2020). Oncogenic roles of the cholesterol metabolite 25-hydroxycholesterol in bladder cancer. Oncol. Lett..

[151] Zhang Z., Yu Y., Zhang Z., Li D., Liang Z., Wang L., Chen Y., Liang Y., Niu H. (2023). Cancer-associated fibroblasts-derived CXCL12 enhances immune escape of bladder cancer through inhibiting P62-mediated autophagic degradation of PDL1. J. Exp. Clin. Cancer Res. CR.

[152] Zheng X., Chen J., Deng M., Ning K., Peng Y., Liu Z., Li X., Zhou Z., Tang H., Li Y. (2024). G3BP1 and SLU7 Jointly Promote Immune Evasion by Downregulating MHC-I via PI3K/Akt Activation in Bladder Cancer. Adv. Sci..

[153] Liu Z., Xin P., Wu W., Jin M., Du Y., Jiang Y., Liu T., Zhang H. (2025). Nanoparticle-mediated targeting of PGC-1α reveals critical metabolic pathways in bladder cancer metastasis. Commun. Biol..

[154] Ma Z., Li X., Mao Y., Wei C., Huang Z., Li G., Yin J., Liang X., Liu Z. (2022). Interferon-dependent SLC14A1(+) cancer-associated fibroblasts promote cancer stemness via WNT5A in bladder cancer. Cancer Cell.

[155] Li W., Shangguan W., Huang W., Zhao J., Zhu Y., Xie M., Yu Y., Yang Q., Zheng J., Yang L. (2025). Gut Parabacteroides distasonis-derived Indole-3-Acetic Acid Promotes Phospholipid Remodeling and Enhances Ferroptosis Sensitivity via the AhR-FASN Axis in Bladder Cancer. Adv. Sci..

[156] Long G.V., Dummer R., Hamid O., Gajewski T.F., Caglevic C., Dalle S., Arance A., Carlino M.S., Grob J.J., Kim T.M. (2019). Epacadostat plus pembrolizumab versus placebo plus pembrolizumab in patients with unresectable or metastatic melanoma (ECHO-301/KEYNOTE-252): A phase 3, randomised, double-blind study. Lancet. Oncol..

[157] Stine Z.E., Schug Z.T., Salvino J.M., Dang C.V. (2022). Targeting cancer metabolism in the era of precision oncology. Nat. Rev. Drug Discov..

[158] Fendt S.M., Frezza C., Erez A. (2020). Targeting Metabolic Plasticity and Flexibility Dynamics for Cancer Therapy. Cancer Discov..

[159] Martínez-Reyes I., Chandel N.S. (2021). Cancer metabolism: Looking forward. Nat. Rev. Cancer.

[160] Loriot Y., Matsubara N., Park S.H., Huddart R.A., Burgess E.F., Houede N., Banek S., Guadalupi V., Ku J.H., Valderrama B.P. (2023). Erdafitinib or Chemotherapy in Advanced or Metastatic Urothelial Carcinoma. N. Engl. J. Med..

[161] Wu J., Cheng B., Song Y., Cheng J., Gao H., Luo X., Huang M., Wang F., Du Y., Qin C. (2026). Targeting Sialylation Enhances the Therapeutic Efficacy of the Nectin-4-Targeted Antibody-Drug Conjugate Enfortumab Vedotin in Bladder Cancer. Cancer Res..

[162] Chelakkot C., Chelakkot V.S., Shin Y., Song K. (2023). Modulating Glycolysis to Improve Cancer Therapy. Int. J. Mol. Sci..

[163] Liu S., Wang X., Sun X., Wei B., Jiang Z., Ouyang Y., Ozaki T., Yu M., Liu Y., Zhang R. (2024). Oridonin inhibits bladder cancer survival and immune escape by covalently targeting HK1. Phytomed. Int. J. Phyther. Phytopharm..

[164] Wu M., Lin P., Xu L., Yu Z., Chen Q., Gu H., Liu C. (2020). Prognostic Role of Serum Lactate Dehydrogenase in Patients With Urothelial Carcinoma: A Systematic Review and Meta-Analysis. Front. Oncol..

[165] Wei X., Chai Y., Li Z., Che X., Zhang Y., Zhou Z., Wang X. (2023). Up-regulated serum lactate dehydrogenase could become a poor prognostic marker in patients with bladder cancer by an evidence-based analysis of 2,182 patients. Front. Oncol..

[166] Daneshmandi S., Wegiel B., Seth P. (2019). Blockade of Lactate Dehydrogenase-A (LDH-A) Improves Efficacy of Anti-Programmed Cell Death-1 (PD-1) Therapy in Melanoma. Cancers.

[167] Qiao T., Xiong Y., Feng Y., Guo W., Zhou Y., Zhao J., Jiang T., Shi C., Han Y. (2021). Inhibition of LDH-A by Oxamate Enhances the Efficacy of Anti-PD-1 Treatment in an NSCLC Humanized Mouse Model. Front. Oncol..

[168] Aminzadeh Z., Ziamajidi N., Abbasalipourkabir R. (2022). Anticancer Effects of Cinnamaldehyde Through Inhibition of ErbB2/HSF1/LDHA Pathway in 5637 Cell Line of Bladder Cancer. Anti-Cancer Agents Med. Chem..

[169] Sellam L.S., Zappasodi R., Chettibi F., Djennaoui D., Yahi-Ait Mesbah N., Amir-Tidadini Z.C., Touil-Boukoffa C., Ouahioune W., Merghoub T., Bourouba M. (2020). Silibinin down-regulates PD-L1 expression in nasopharyngeal carcinoma by interfering with tumor cell glycolytic metabolism. Arch. Biochem. Biophys..

[170] Yang W., Zheng Y., Xia Y., Ji H., Chen X., Guo F., Lyssiotis C.A., Aldape K., Cantley L.C., Lu Z. (2012). ERK1/2-dependent phosphorylation and nuclear translocation of PKM2 promotes the Warburg effect. Nat. Cell Biol..

[171] Jin H., Wu P., Lv C., Zhang S., Zhang Y., Li C., Gao R., Shan G., Bi H., Chang H. (2025). Mannose inhibits PKM2 lactylation to induce pyroptosis in bladder cancer and activate antitumor immune responses. Commun. Biol..

[172] Zhang H., Li J. (2026). Oxymatrine Inhibits PKM2-Mediated Glycolysis and Enhances Cisplatin Sensitivity in Bladder Cancer. Tohoku J. Exp. Med..

[173] Konstantakou E.G., Voutsinas G.E., Velentzas A.D., Basogianni A.S., Paronis E., Balafas E., Kostomitsopoulos N., Syrigos K.N., Anastasiadou E., Stravopodis D.J. (2015). 3-BrPA eliminates human bladder cancer cells with highly oncogenic signatures via engagement of specific death programs and perturbation of multiple signaling and metabolic determinants. Mol. Cancer.

[174] Wang X., Li S., Li Z., Lin Z., Wang Z. (2025). SRT3025-loaded cell membrane hybrid liposomes (3025@ML) enhanced anti-tumor activity of Oxaliplatin via inhibiting pyruvate kinase M2 and fatty acid synthase. Lipids Health Dis..

[175] Denis M., Grasselly C., Choffour P.A., Wierinckx A., Mathé D., Chettab K., Tourette A., Talhi N., Bourguignon A., Birzele F. (2022). In Vivo Syngeneic Tumor Models with Acquired Resistance to Anti-PD-1/PD-L1 Therapies. Cancer Immunol. Res..

[176] Falchook G., Infante J., Arkenau H.T., Patel M.R., Dean E., Borazanci E., Brenner A., Cook N., Lopez J., Pant S. (2021). First-in-human study of the safety, pharmacokinetics, and pharmacodynamics of first-in-class fatty acid synthase inhibitor TVB-2640 alone and with a taxane in advanced tumors. eClinicalMedicine.

[177] Wang G., Cao R., Wang Y., Qian G., Dan H.C., Jiang W., Ju L., Wu M., Xiao Y., Wang X. (2016). Simvastatin induces cell cycle arrest and inhibits proliferation of bladder cancer cells via PPARγ signalling pathway. Sci. Rep..

[178] Deng J., Peng M., Zhou S., Xiao D., Hu X., Xu S., Wu J., Yang X. (2021). Metformin targets Clusterin to control lipogenesis and inhibit the growth of bladder cancer cells through SREBP-1c/FASN axis. Signal Transduct. Target. Ther..

[179] Saito A., Kitayama J., Horie H., Koinuma K., Ohzawa H., Yamaguchi H., Kawahira H., Mimura T., Lefor A.K., Sata N. (2020). Metformin changes the immune microenvironment of colorectal cancer in patients with type 2 diabetes mellitus. Cancer Sci..

[180] Afzal M.Z., Mercado R.R., Shirai K. (2018). Efficacy of metformin in combination with immune checkpoint inhibitors (anti-PD-1/anti-CTLA-4) in metastatic malignant melanoma. J. Immunother. Cancer.

[181] Tannir N.M., Agarwal N., Porta C., Lawrence N.J., Motzer R., McGregor B., Lee R.J., Jain R.K., Davis N., Appleman L.J. (2022). Efficacy and Safety of Telaglenastat Plus Cabozantinib vs Placebo Plus Cabozantinib in Patients With Advanced Renal Cell Carcinoma: The CANTATA Randomized Clinical Trial. JAMA Oncol..

[182] Leone R.D., Zhao L., Englert J.M., Sun I.M., Oh M.H., Sun I.H., Arwood M.L., Bettencourt I.A., Patel C.H., Wen J. (2019). Glutamine blockade induces divergent metabolic programs to overcome tumor immune evasion. Science.

[183] Watson M.J., Vignali P.D.A., Mullett S.J., Overacre-Delgoffe A.E., Peralta R.M., Grebinoski S., Menk A.V., Rittenhouse N.L., DePeaux K., Whetstone R.D. (2021). Metabolic support of tumour-infiltrating regulatory T cells by lactic acid. Nature.

[184] Wang J.X., Choi S.Y.C., Niu X., Kang N., Xue H., Killam J., Wang Y. (2020). Lactic Acid and an Acidic Tumor Microenvironment suppress Anticancer Immunity. Int. J. Mol. Sci..

[185] Renner K., Bruss C., Schnell A., Koehl G., Becker H.M., Fante M., Menevse A.N., Kauer N., Blazquez R., Hacker L. (2019). Restricting Glycolysis Preserves T Cell Effector Functions and Augments Checkpoint Therapy. Cell Rep..

[186] Zou W., Green D.R. (2023). Beggars banquet: Metabolism in the tumor immune microenvironment and cancer therapy. Cell Metab..

[187] Li Z., Wang Q., Huang X., Yang M., Zhou S., Li Z., Fang Z., Tang Y., Chen Q., Hou H. (2023). Lactate in the tumor microenvironment: A rising star for targeted tumor therapy. Front. Nutr..

[188] Li Z.A., Wen K.C., Liu J.H., Zhang C., Zhang F., Li F.Q. (2024). Strategies for intravesical drug delivery: From bladder physiological barriers and potential transport mechanisms. Acta Pharm. Sin. B.

[189] Vainchtein L.D., Rosing H., Mirejovsky D., Lenaz L., Schellens J.H., Beijnen J.H. (2007). Stability experiments in human urine with EO9 (apaziquone): A novel anticancer agent for the intravesical treatment of bladder cancer. J. Pharm. Biomed. Anal..

[190] Shangguan X., Ma Z., Yu M., Ding J., Xue W., Qi J. (2022). Squalene Epoxidase Metabolic Dependency Is a Targetable Vulnerability in Castration-Resistant Prostate Cancer. Cancer Res..

[191] Li X., Peng X., Zhang C., Bai X., Li Y., Chen G., Guo H., He W., Zhou X., Gou X. (2022). Bladder Cancer-Derived Small Extracellular Vesicles Promote Tumor Angiogenesis by Inducing HBP-Related Metabolic Reprogramming and SerRS O-GlcNAcylation in Endothelial Cells. Adv. Sci..

[192] Molina J.R., Sun Y., Protopopova M., Gera S., Bandi M., Bristow C., McAfoos T., Morlacchi P., Ackroyd J., Agip A.A. (2018). An inhibitor of oxidative phosphorylation exploits cancer vulnerability. Nat. Med..

[193] Jung K.H., LoRusso P., Burris H., Gordon M., Bang Y.J., Hellmann M.D., Cervantes A., Ochoa de Olza M., Marabelle A., Hodi F.S. (2019). Phase I Study of the Indoleamine 2,3-Dioxygenase 1 (IDO1) Inhibitor Navoximod (GDC-0919) Administered with PD-L1 Inhibitor (Atezolizumab) in Advanced Solid Tumors. Clin. Cancer Res. An. Off. J. Am. Assoc. Cancer Res..

[194] Fiehn O. (2002). Metabolomics—The link between genotypes and phenotypes. Plant Mol. Biol..

[195] Chen Y., Li E.M., Xu L.Y. (2022). Guide to Metabolomics Analysis: A Bioinformatics Workflow. Metabolites.

[196] Wishart D.S., Cheng L.L., Copié V., Edison A.S., Eghbalnia H.R., Hoch J.C., Gouveia G.J., Pathmasiri W., Powers R., Schock T.B. (2022). NMR and Metabolomics-A Roadmap for the Future. Metabolites.

[197] Guo J., Yu H., Xing S., Huan T. (2022). Addressing big data challenges in mass spectrometry-based metabolomics. Chem. Commun..

[198] Wittmann B.M., Stirdivant S.M., Mitchell M.W., Wulff J.E., McDunn J.E., Li Z., Dennis-Barrie A., Neri B.P., Milburn M.V., Lotan Y. (2014). Bladder cancer biomarker discovery using global metabolomic profiling of urine. PLoS ONE.

[199] Nizioł J., Ossoliński K., Płaza-Altamer A., Kołodziej A., Ossolińska A., Ossoliński T., Krupa Z., Ruman T. (2024). Untargeted metabolomics of bladder tissue using liquid chromatography and quadrupole time-of-flight mass spectrometry for cancer biomarker detection. J. Pharm. Biomed. Anal..

[200] Nizioł J., Ossoliński K., Płaza-Altamer A., Kołodziej A., Ossolińska A., Ossoliński T., Nieczaj A., Ruman T. (2023). Untargeted urinary metabolomics for bladder cancer biomarker screening with ultrahigh-resolution mass spectrometry. Sci. Rep..

[201] Ossoliński K., Ruman T., Copié V., Tripet B.P., Kołodziej A., Płaza-Altamer A., Ossolińska A., Ossoliński T., Nieczaj A., Nizioł J. (2023). Targeted and untargeted urinary metabolic profiling of bladder cancer. J. Pharm. Biomed. Anal..

[202] Razavi S., Khan A., Fu D.X., Mayer D., McConkey D., Putluri N., Siddiqui M.M. (2025). Metabolic landscape in bladder cancer. Curr. Opin. Oncol..

[203] Sumner L.W., Amberg A., Barrett D., Beale M.H., Beger R., Daykin C.A., Fan T.W., Fiehn O., Goodacre R., Griffin J.L. (2007). Proposed minimum reporting standards for chemical analysis Chemical Analysis Working Group (CAWG) Metabolomics Standards Initiative (MSI). Metabolomics Off. J. Metabolomic Soc..

[204] Zhou Y., Song R., Zhang Z., Lu X., Zeng Z., Hu C., Liu X., Li Y., Hou J., Sun Y. (2016). The development of plasma pseudotargeted GC-MS metabolic profiling and its application in bladder cancer. Anal. Bioanal. Chem..

[205] Liu Z., Teng C., Wan W., Wu F., Wu C., Ji W., Shan Y. (2024). A panel of four plasma amino acids is a promising biomarker for newly diagnosed bladder cancer. Clin. Nutr..

[206] Wang Z., Liu X., Liu X., Sun H., Guo Z., Zheng G., Zhang Y., Sun W. (2019). UPLC-MS based urine untargeted metabolomic analyses to differentiate bladder cancer from renal cell carcinoma. BMC Cancer.

[207] Piyarathna D.W.B., Rajendiran T.M., Putluri V., Vantaku V., Soni T., von Rundstedt F.C., Donepudi S.R., Jin F., Maity S., Ambati C.R. (2018). Distinct Lipidomic Landscapes Associated with Clinical Stages of Urothelial Cancer of the Bladder. Eur. Urol. Focus.

[208] González-Domínguez R., González-Domínguez Á., Sayago A., Fernández-Recamales Á. (2020). Recommendations and Best Practices for Standardizing the Pre-Analytical Processing of Blood and Urine Samples in Metabolomics. Metabolites.

[209] Singh V., Singh M.K., Kumar A., Sahu D.K., Jain M., Pandey A.K., Mantasha, Singh S., Verma A.K. (2025). Metabolomic Biomarkers for Prognosis in Non-Muscle Invasive Bladder Cancer: A Comprehensive Systematic Review and Meta-Analysis. Indian J. Clin. Biochem. IJCB.

[210] Jacyna J., Kordalewska M., Artymowicz M., Markuszewski M., Matuszewski M., Markuszewski M.J. (2022). Pre- and Post-Resection Urine Metabolic Profiles of Bladder Cancer Patients: Results of Preliminary Studies on Time Series Metabolomics Analysis. Cancers.

[211] Artymowicz M., Struck-Lewicka W., Wiczling P., Markuszewski M., Markuszewski M.J., Siluk D. (2023). Targeted quantitative metabolomics with a linear mixed-effect model for analysis of urinary nucleosides and deoxynucleosides from bladder cancer patients before and after tumor resection. Anal. Bioanal. Chem..

[212] Madej A., Forma E., Golberg M., Kamiński R., Paneth P., Kobos J., Różański W., Lipiński M. (2022). (13)C Natural Isotope Abundance in Urothelium as a New Marker in the Follow-Up of Patients with Bladder Cancer. Cancers.

[213] Fattahi M.J., Sedaghat F., Haghshenas M.R., Ariafar A., Shiravani Z., Malekzadeh M., Madani S. (2024). Two metabolic enzymes, LDH and FASN, serum levels in Bladder cancer patients. Casp. J. Intern. Med..

[214] Xu Y., Xia Z., Sun X., Wei B., Fu Y., Shi D., Zhu Y. (2023). Identification of a glutamine metabolism reprogramming signature for predicting prognosis, immunotherapy efficacy, and drug candidates in bladder cancer. Front. Immunol..

[215] Song T., He K., Ning J., Li W., Xu T., Yu W., Rao T., Cheng F. (2022). Evaluation of aliphatic acid metabolism in bladder cancer with the goal of guiding therapeutic treatment. Front. Oncol..

[216] Shen C., Suo Y., Guo J., Su W., Zhang Z., Yang S., Wu Z., Fan Z., Zhou X., Hu H. (2024). Development and validation of a glycolysis-associated gene signature for predicting the prognosis, immune landscape, and drug sensitivity in bladder cancer. Front. Immunol..

[217] Li T., You Q., Zhang S., Li R., Xie S., Li D., Ai S., Yang R., Guo H. (2024). Performance of (18)F-FDG PET/MRI and its parameters in staging and neoadjuvant therapy response evaluation in bladder cancer. iScience.

[218] van Ginkel N., van Gennep E.J., Oosterbaan L., Greidanus J., Boellaard T.N., Wondergem M., Vis A.N., de Reijke T.M., van Rhijn B.W.G., Mertens L.S. (2023). Added Clinical Value of (18)F-FDG-PET/CT to Stage Patients With High-Risk Non-Muscle Invasive Bladder Cancer Before Radical Cystectomy. Clin. Genitourin. Cancer.

[219] Zhang-Yin J., Girard A., Marchal E., Lebret T., Homo Seban M., Uhl M., Bertaux M. (2023). PET Imaging in Bladder Cancer: An Update and Future Direction. Pharmaceuticals.

[220] Pan F., Liu X., Wan J., Guo Y., Sun P., Zhang X., Wang J., Bao Q., Yang L. (2024). Advances and prospects in deuterium metabolic imaging (DMI): A systematic review of in vivo studies. Eur. Radiol. Exp..

[221] Wodtke P., McLean M.A., Horvat-Menih I., Birchall J.R., Zamora-Morales M.J., Grimmer A., Latimer E., Wylot M., Schulte R.F., Gallagher F.A. (2026). Deuterium Metabolic Imaging of the Human Abdomen at Clinical Field Strength. Invest Radiol..

[222] Bizzarri F.P., Campetella M., Russo P., Palermo G., Moosavi S.K., Rossi F., D’Amico L., Cretì A., Gavi F., Panio E. (2025). Prognostic Value of PLR, SIRI, PIV, SII, and NLR in Non-Muscle Invasive Bladder Cancer: Can Inflammatory Factors Influence Pathogenesis and Outcomes?. Cancers.

[223] Weber C.M., Cauchi M., Patel M., Bessant C., Turner C., Britton L.E., Willis C.M. (2011). Evaluation of a gas sensor array and pattern recognition for the identification of bladder cancer from urine headspace. Analyst.

[224] Myridakis A., Wen Q., Boshier P.R., Parker A.G., Belluomo I., Handakas E., Hanna G.B. (2023). Global Urinary Volatolomics with (GC×)GC-TOF-MS. Anal. Chem..

[225] Carapito Â., Roque A.C.A., Carvalho F., Pinto J., Guedes de Pinho P. (2024). Exploiting volatile fingerprints for bladder cancer diagnosis: A scoping review of metabolomics and sensor-based approaches. Talanta.

[226] Lett L., George M., Slater R., De Lacy Costello B., Ratcliffe N., García-Fiñana M., Lazarowicz H., Probert C. (2022). Investigation of urinary volatile organic compounds as novel diagnostic and surveillance biomarkers of bladder cancer. Br. J. Cancer.

[227] Pinto J., Carapito Â., Amaro F., Lima A.R., Carvalho-Maia C., Martins M.C., Jerónimo C., Henrique R., Bastos M.L., Guedes de Pinho P. (2021). Discovery of Volatile Biomarkers for Bladder Cancer Detection and Staging through Urine Metabolomics. Metabolites.

[228] Oto J., Fernández-Pardo Á., Roca M., Plana E., Cana F., Herranz R., Pérez-Ardavín J., Vera-Donoso C.D., Martínez-Sarmiento M., Medina P. (2022). LC-MS metabolomics of urine reveals distinct profiles for non-muscle-invasive and muscle-invasive bladder cancer. World J. Urol..

[229] Elsharkawi F., Elsabah M., Shabayek M., Khaled H. (2019). Urine and Serum Exosomes as Novel Biomarkers in Detection of Bladder Cancer. Asian Pac. J. Cancer Prev. APJCP.

[230] Steiner L., Eldh M., Offens A., Veerman R.E., Johansson M., Hemdan T., Netterling H., Huge Y., Abdul-Sattar Aljabery F., Alamdari F. (2025). Protein profile in urinary extracellular vesicles is a marker of malignancy and correlates with muscle invasiveness in urinary bladder cancer. Cancer Lett..

[231] Schwaiger-Haber M., Stancliffe E., Anbukumar D.S., Sells B., Yi J., Cho K., Adkins-Travis K., Chheda M.G., Shriver L.P., Patti G.J. (2023). Using mass spectrometry imaging to map fluxes quantitatively in the tumor ecosystem. Nat. Commun..

[232] Nunes J.B., Ijsselsteijn M.E., Abdelaal T., Ursem R., van der Ploeg M., Giera M., Everts B., Mahfouz A., Heijs B., de Miranda N. (2024). Integration of mass cytometry and mass spectrometry imaging for spatially resolved single-cell metabolic profiling. Nat. Methods.

[233] Lindskrog S.V., Schmøkel S.S., Nordentoft I., Lamy P., Knudsen M., Prip F., Strandgaard T., Jensen J.B., Dyrskjøt L. (2023). Single-nucleus and Spatially Resolved Intratumor Subtype Heterogeneity in Bladder Cancer. Eur. Urol. Open Sci..

[234] Minoli M., Cantore T., Hanhart D., Kiener M., Fedrizzi T., La Manna F., Karkampouna S., Chouvardas P., Genitsch V., Rodriguez-Calero A. (2023). Bladder cancer organoids as a functional system to model different disease stages and therapy response. Nat. Commun..

[235] Merrill N.M., Kaffenberger S.D., Bao L., Vandecan N., Goo L., Apfel A., Cheng X., Qin Z., Liu C.J., Bankhead A. (2024). Integrative Drug Screening and Multiomic Characterization of Patient-derived Bladder Cancer Organoids Reveal Novel Molecular Correlates of Gemcitabine Response. Eur. Urol..

[236] Daneshmand S., Kamat A.M., Shore N.D., Meeks J.J., Galsky M.D., Jacob J.M., van der Heijden M.S., Williams S.B., Powles T., Chang S.S. (2025). Development of TAR-200: A novel targeted releasing system designed to provide sustained delivery of gemcitabine for patients with bladder cancer. Urol. Oncol..

