# Supplementary material for: Metabolic Reprogramming in Bladder Cancer: Molecular Drivers, Immune Regulation and Therapeutic Opportunities
Source: Cancers (Basel). 2026 Oct 3;18(19):3202. doi: 10.3390/cancers18193202 (PMC13664454; doi:10.3390/cancers18193202)
Supplement: Supplementary file 1 [file cancers-18-03202-s001.zip › cancers-4541672-supplementary.pdf]

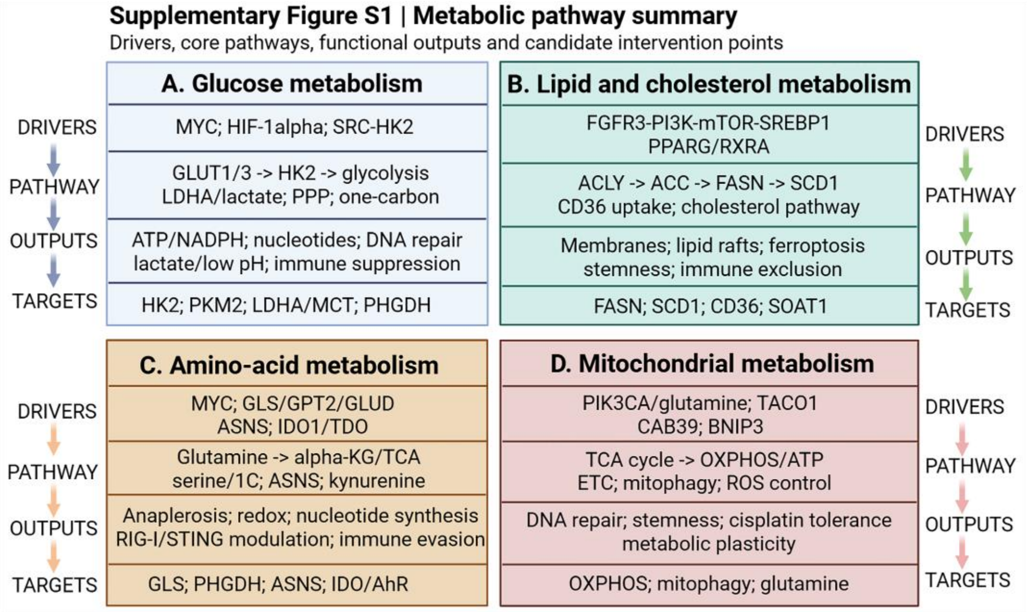

Supplementary Figure S1. Schematic summary of metabolism-related therapeutic vulnerabilities in bladder cancer. The figure summarizes four major metabolic programs discussed in the revised manuscript: glucose metabolism, lipid and cholesterol metabolism, amino-acid metabolism, and mitochondrial metabolism. Each panel highlights representative molecular drivers, altered metabolic pathways, functional outputs, and potential therapeutic targets relevant to bladder cancer progression, treatment resistance, and immune modulation. Abbreviations: OXPHOS, oxidative phosphorylation; PPP, pentose phosphate pathway; TCA, tricarboxylic acid cycle; ROS, reactive oxygen species.
